# Prevalence and antimicrobial resistance of *Campylobacter jejuni* and *Campylobacter coli* over time in Thailand under a One Health approach: A systematic review and meta-analysis

**DOI:** 10.1016/j.onehlt.2025.100965

**Published:** 2025-01-10

**Authors:** Doan Hoang Phu, Tuempong Wongtawan, Truong Thanh Nam, Dinh Bao Truong, Naparat Suttidate, Juan Carrique-Mas, Niwat Chansiripornchai, Conny Turni, Patrick J. Blackall, Thotsapol Thomrongsuwannakij

**Affiliations:** a Akkhraratchakumari Veterinary College, Walailak University, Nakhon Si Thammarat 80160, Thailand; b Faculty of Animal Science and Veterinary Medicine, Nong Lam University, Ho Chi Minh City 70000, Viet Nam; c Centre for One Health, Walailak University, Nakhon Si Thammarat 80160, Thailand; d Faculty of Public Health, Can Tho University of Medicine and Pharmacy, Can Tho 94000, Viet Nam; e Food and Agriculture Organization of the United Nations, Ha Noi 10000, Viet Nam; f Avian Health Research Unit, Department of Veterinary Medicine, Faculty of Veterinary Science, Chulalongkorn University, Bangkok, Thailand; g The University of Queensland, St. Lucia 4067, Australia

**Keywords:** Prevalence, Sequence types, Phenotypic resistance, Genotypic resistance, Review, Thai

## Abstract

*Campylobacter* spp. are major food-borne zoonotic pathogens impacting food safety worldwide. Thailand is one of the countries facing with a significant burden of *Campylobacter* infections and is recognized as a hotspot of AMR. Our study applied a systematic review and meta-analysis, using a One Health perspective, to investigate the prevalence and AMR of *Campylobacter jejuni* (*C. jejuni*) and *Campylobacter coli* (*C. coli*) over time in Thailand, from 1985 to 2023. Based on the PRISMA guidelines, a literature search using PubMed, ScienceDirect, and Google Scholar to identify the articles reporting prevalence, sequence types (STs), antimicrobial susceptibility, and resistance genes of *Campylobacter* spp. in humans, animals, food, and environmental samples was performed. Eighty-one articles were retrieved for systematic review, with 33 reporting *Campylobacter* spp. prevalence and 20 containing AMR data collected for meta-analysis. The highest prevalence of *C. jejuni* was found in chickens (43.6 %) and chicken products (31.4 %), followed by ducks (16.7 %), the general human population with diarrhea (15.9 %), children with diarrhea (10.7 %). *C. coli* was also prevalent in chickens (12.6 %) and chicken products (10.4 %). *C. jejuni* prevalence decreased by 14.8 % among children with diarrhea (*p* = 0.006), but increased by 16.7 % in chicken products (*p* = 0.007). Sixty-two STs were identified, with ST 574, ST 1075, ST 51 being the most prevalent STs recorded. Five STs, including ST 50, ST 51, ST 354, ST 464, and ST 574, were reported in both humans and chickens. The AMR levels were highest against quinolones, ranging 75.4 %–94.8 % in human-related categories and 71.6 %–88.7 % in chicken-related categories. Notably, ciprofloxacin-resistant and nalidixic acid-resistant *C. jejuni* strains collected from chickens increased by 11.9 % (*p* = 0.004) and 16.1 % (*p* = 0.027), respectively. Thirteen resistance genes/mutations were reported, with the phenotypic resistance linked to *gyrA* mutations and *tet*(O) genes. The high prevalence and increasing trend of AMR in *C. jejuni* and *C. coli* underscore the critical need for One Health surveillance to address the rising AMR challenge posed by these pathogens in Thailand.

## Highlights

• *C. jejuni* was prevalent in chickens/products, *C. coli* dominated farm environments in Thailand.
• *C. jejuni* prevalence dropped in children with diarrhea but rose in chicken products.
• Many shared STs of *C. jejuni* were found in both Thai humans and chickens.
• High and rising (fluoro)quinolone resistance of strains from humans and chickens was revealed.
• Strong pheno-genotype correlations were found for (fluoro)quinolone and tetracycline resistance.

## 1 Introduction

*Campylobacter* spp. are prominent zoonotic pathogens causing human gastrointestinal infections via foodborne transmission, contact with infected animals, person-to-person spread, and environmental reservoirs [1]. In spite of the impacts on human health and food safety, *Campylobacter* spp. have been neglected for the past decades, and the associated burden of infection is underestimated particularly in low- and middle-income countries (LMICs) [2]. Globally, *Campylobacter* spp. were responsible for 96 million cases of diarrheal illness, resulting in 37,600 deaths in 2010, with Africa and Southeast Asia known as the hotspots of *Campylobacter* infections [3,4]. The economic losses caused by *Campylobacter* infections are due to the burden of illness and the costs to the food animal industries, which include farm-level interventions and surveillance and regulatory measures aimed at ensuring food safety [5]. Human bacterial gastroenteritis is predominantly caused by two *Campylobacter* species, with *C. jejuni* responsible for 90 % of cases and *C. coli* accounting for the remaining ∼10 % [6]. Among animal species, chickens are the main reservoir and a primary source of *Campylobacter* spp. to humans [7]. Risk factors for *Campylobacter* infections include consuming under-processed meat, exposure to contaminated food, and direct contact with animals [8]. Individuals over 65 years and children under 5 years, face higher risks of severe campylobacteriosis [9,10].

Like other Southeast Asian countries, Thailand is affected by a substantial burden of *Campylobacter* infections. *Campylobacter* spp. were identified as the cause of 34.8 % of diarrhea cases (54/155), with *C. jejuni* accounting for 83.3 % and *C. coli* for 16.7 % in US military based in Thailand [11]. The prevalence of *Campylobacter* spp. was significantly higher in individuals with acute diarrhea, ranging from 4.2 to 10.1 times greater than those with enteric carriage [12,13]. However, a case-control study on under-five year old children in a remote area revealed a nonsignificant difference in the prevalence of *Campylobacter* spp. isolated from children with diarrhea (22 %) compared to those without diarrhea (25 %) [14]. Additionally, Thailand is known as a hotspot of antimicrobial resistance (AMR) in animals and food products, particularly in bacterial species that serve as indicators of resistance trends, including *Escherichia coli*, nontyphoidal *Salmonella* spp., *Campylobacter* spp., and *Staphylococcus aureus* [15]. The misuse and overuse of antimicrobials in animal production systems is one of the primary factors for the emergence of AMR [16,17]. For the treatment of campylobacteriosis, the medications of choice are macrolides and quinolones [18,19]. However, there is a rising global prevalence of *Campylobacter* spp. resistant to these antimicrobials [20]. This is a problematic situation for the Thai food animal industries. Despite being among the highest priority critically important antimicrobials classified by WHO [21], macrolides and fluoroquinolones were the most used agents in food-producing animals in Thailand [22].

Several reviews on the prevalence and AMR of *Campylobacter* spp. have been conducted in Thailand. These reviews have investigated the prevalence of *Campylobacter* isolates in either humans with diarrhea [23] or animals (i.e., pigs and chickens) [24]. A review of AMR trends covered the prevalence of *Campylobacter* over 15 years in Thailand (1981–1995), focusing exclusively on humans with diarrhea [25]. Another recent study investigated the AMR prevalence of *Campylobacter* spp. isolates from humans and animals across Southeast Asia countries, including Thailand. However, this study reviewed the literatures focusing on the AMR prevalence of *Campylobacter* spp. against a narrow scope of only fluoroquinolones and tetracyclines [26]. Therefore, the present review aimed to provide an updated investigation of the prevalence of *Campylobacter* spp. as well as their sequence types, phenotypic and genotypic AMR under a One Health approach, while examining the temporal trends in the prevalence and AMR issues of *Campylobacter* spp. in Thailand. This study provides a comprehensive summary of Thai studies on *Campylobacter* spp., serving as a cornerstone for further investigations and contributing to strategies aimed at reducing AMR in Thailand.

## 2 Materials and methods

### 2.1 Study protocol and search strategy

The literature on *Campylobacter* spp. in Thailand was reviewed in adherence to the PRISMA guideline, which includes a total of 27 checklist items [27] (Supplementary Table 1**)**. The keywords used for searching on PubMed were: (Campylobacter*[ti] OR Campylobacteriosis*[ti]) AND (Prevalence[tiab] OR Antimicrobial resistance*[tiab] OR Antimicrobial susceptibility [tiab] OR Phenotyp* OR Genotyp* OR Thailand OR Thai) NOT review [ptyp]; while the key words searched on ScienceDirect were [*Campylobacter* OR Campylobacteriosis] AND [Prevalence OR Antimicrobial resistance OR Phenotyp* OR Genotyp*] AND [Thailand OR Thai]. Moreover, manual-searching from the reference lists of selected studies were also performed on Google Scholar to have a higher number of eligible articles. The protocol for this review was not registered with the International Prospective Register of Systematic Reviews (PROSPERO) because our review focuses on the prevalence of *Campylobacter* spp. and the AMR situation over time under the One Health approach. The specific requirements for either human or animal research studies in PROSPERO do not align with the comprehensive scope of our review.

### 2.2 Inclusion and exclusion criteria for article selection

Article selection for the review was conducted based on three stages: title, abstract and full text. Initially, searches were performed on PubMed, ScienceDirect and Google Scholar, yielding a list of all titles of relevant articles. For inclusion, the articles were selected if their title and abstracts provided information on: (1) prevalence of *Campylobacter* isolates detected; (2) sequence types (STs); (3) antimicrobial susceptibility; and (4) antimicrobial resistance genes (ARGs)/mutations. During the full-text screening, articles included had to describe the prevalence of *Campylobacter* spp., and with the sample collection being from: (1) humans (carriage/ diarrhea); (2) animals (e.g., chicken, pig, ruminant)/ animal products (e.g., meat, egg, and milk) and their environments (e.g., litter, water, pests, feed); and (3) non-animal derived food samples (e.g., salad, fruit, vegetable). For exclusion, articles without any information on either the *Campylobater* spp. prevalence or STs or antimicrobial susceptibility or detection of ARGs/mutations were excluded. Review articles, book chapters, conference abstracts, letters, and articles written in other languages than English, and duplicates were also excluded.

To minimize the selection bias, the Joanna Briggs Institute (JBI) Critical Appraisal Checklist, specifically designed for studies reporting prevalence data, was applied to evaluate the quality of the included studies [28]. The checklist contains nine questions reviewed by two independent researchers (T.T.N and D.B.T), with response options of ‘yes’, ‘no’, ‘unclear’, and ‘not applicable’. Only studies that received a ‘yes’ for all questions were selected. In cases where discrepancies in study selection between the two reviewers occurred, a third reseacher (D.H·P) participated in the selection to resolve any disagreements regarding article inclusion. Studies were included based on a consensus of “yes” from at least two out of the three researchers (Supplementary Table 2).

### 2.3 Data extraction

From each study, the information was extracted as follows: (1) year of sample collection; (2) sources of samples collected (humans, animals, animal derived products, non-animal derived product, and environmental samples); (3) sample types (faeces, stool, rectal swabs, blood, floor swabs, farm waste, carcass, meat, milk, and environmental samples); (4) number of samples collected; (5) methods of *Campylobacter* spp. identification; (6) prevalence of *Campylobacter* spp.; (7) typing methods of *Campylobacter* spp.; (8) STs using multilocus sequence typing (MLST); (8) methods of antimicrobial susceptibility testing; (9) prevalence of phenotypic resistance; (10) genotypic resistance (ARGs and mutations).

In the case of an absence of an explicit date of sample collection, a date of two years before publication was assigned while for studies conducted over a time period, a mid-point was defined as described by a previous study [29]. For example, if a study spanned five years (2011–2015), the sample collection year was assigned as 2013. Human isolates were further categorized based on whether *Campylobacter* spp. were isolated from healthy subjects (i.e. enteric carriage), or from cases of enteric disease (i.e., diarrhea) by different age groups including <5 years old (children), >60 years old (elders), and from 5 to 60 years old (general population). Animal isolates were also classified based on the different animal species including chickens, ducks, pigs, aquatic animals, and ruminants. Isolates obtained from animal-derived products were categorized by the origin of animal species, including chicken products (chicken meat, eggs), duck products (duck meat, eggs), pork, ruminant products (beef, milk, lamb, goat meat). Isolates detected from salad, fruit, and vegetable samples were categorized as non-animal derived products. Isolates of samples collected at the animal house facilities (e.g., litter, feeders, footwear, water, and feed) were categorized as environmental isolates. Two authors (T.T.N and D.B.T) independently participated in data extraction and cross-checked the extracted data for consistency to mitigate information bias. In case of discrepancies during data extraction, a third reseacher (D.H·P) was responsible for making a final decision.

### 2.4 Data analyses

The extracted data was synthesized using descriptive analyses, including the calculation of proportions, mean, and associated standard error (SE). Only studies reporting the prevalence of *Campylobacter* spp. detection and/or phenotypic resistance data were included in the meta-analyses. Moreover, meta-analysis was performed from included studies with the prevalence data extracted from at least two articles by the sources of isolates categorized as follows: (1) humans with enteric carriage. (2) humans with diarrhea, (3) poultry/poultry meat; (4) pigs/pork; (5) aquatic animals/seafood; (6) ruminant/ ruminant products; (7) non-animal derived food samples, and (8) environment samples.

Heterogeneity across selected studies was assessed using the inverse variance index (*I*^*2*^), with *I*^*2*^ > 75 %, *p*-value <0.05 indicating significant heterogeneity, as described by previous studies [30,31]. Logit-transformed proportions were analyzed using a generalized linear mixed-effect model, including a random-effect model to identify the within- and between-study variances [32]. The results of the meta-analysis were presented using forest plots. Univariable meta-regression models were performed to investigate the trends in *Campylobacter* prevalence and phenotypic resistance. Additionally, sensitivity analyses were performed to assess the impact of influential studies with data extracted based on sampling year assumptions. The results of the main analysis which included all studies (both those with data collected using assumptions and those without) were compared with the results after removing influential studies.

Two methods were used to assess publication bias in the meta-analysis. Contour-enhanced funnel plots were used to visualize the asymmetrical patterns indicating publication bias. Subsequently, Egger's regression test was performed based on the linear regression model to identify the publication bias by testing for asymmetry in the funnel plots. The presence of publication bias was suggested in cases where the intercept (*βo*) deviated from zero, indicating funnel plot asymmetry [32].

All statistical analyses and figures were performed using R programming language [33], with the ‘meta’ package, and ‘metafor’ package used for meta-analysis and univariable meta-regression models [34,35]. Package tidyverse’ facilitated the evaluation of publication bias [36]. Also, package ‘ggplot2’ was used to visualize the study results [37]. Moreover, all STs reported were visualized by constructing a minimum spanning tree using the goeBURST algorithm in the PHYLOViZ software (http://www.phyloviz.net/).

## 3 Results

### 3.1 Article selection process

**Fig. 1:**
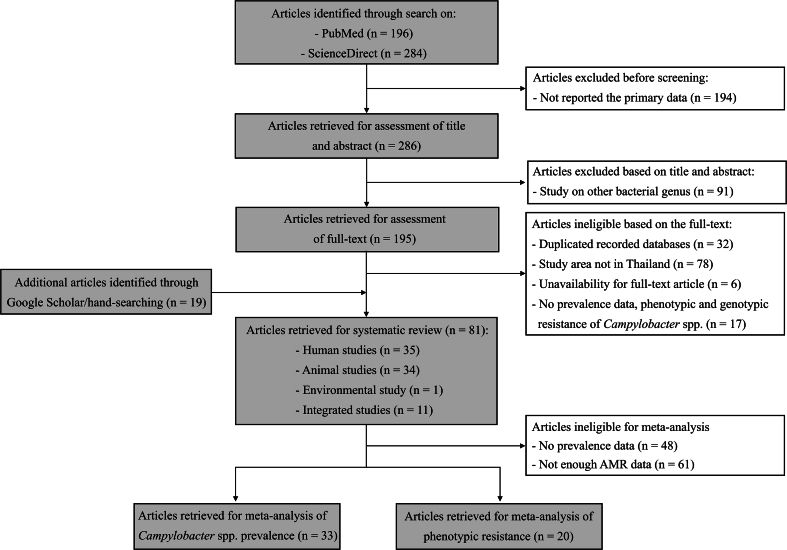
The PRISMA flow diagram of the study selection.

A total of 480 articles were identified during the initial search on PubMed and ScienceDirect. Of these papers, 286 articles that met the criteria for publishing primary data were selected. However, 91 articles were excluded due to the absence of *Campylobacter* data in their title and abstracts. Consequently, 195 papers remained for evaluation using the full-text of the publication. Subsequently, a further refinement process was undertaken by excluding 39 articles that were duplicated among the databases, 78 articles describing work conducted in countries other than Thailand, and 17 articles that lacked data on *Campylobacter* prevalence, sequence types (STs), or phenotypic and genotypic antimicrobial resistance. Full texts were unavailable for six articles (primarily published in the 1980s and 2000s) and did not contain contact information for a corresponding author. After hand-searching on Google Scholar, a further 19 eligible articles were included, resulting in a total of 81 articles for the systematic review. Additionally, to conduct meta-analyses, articles reporting the prevalence of *Campylobacter* isolates, including *C. jejuni* and/or *C. coli* (*n* = 33), and the phenotypic antimicrobial resistance (*n* = 20) were chosen for meta-analyses. The process of article selection is shown in Fig. 1.

### 3.2 Characteristics of selected studies

Of the 81 selected studies, 35 (43.2 %) included *Campylobacter* spp. isolated from humans: 13 studies focused on enteric diseases in children with diarrhea, 9 studies on enteric diseases in the general population, 4 studies on both enteric disease and carriage in the general population, 3 studies on both enteric disease and carriage in children, 2 studies on carriage in the general population, and 4 studies on humans without specifying the age group. A total of thirteen studies (16.0 %) investigated *Campylobacter* spp. in animals, specifically chickens (eight studies), ducks (two studies), pigs (one study), ruminants (one study), and a combination of pigs and ruminants (one study). Regarding animal products, thirteen studies (16.0 %) were conducted, in which most focused on samples collected from chicken products (ten studies), while others investigated *Campylobacter* spp. in mixed samples from chicken, pork, ruminants, and seafood. Only one study was categorized as non-animal-derived products. Additionally, nineteen studies were classified as integrated studies, as they collected samples from various sources, including humans, animals, animal products, and environmental samples.

**Fig. 2:**
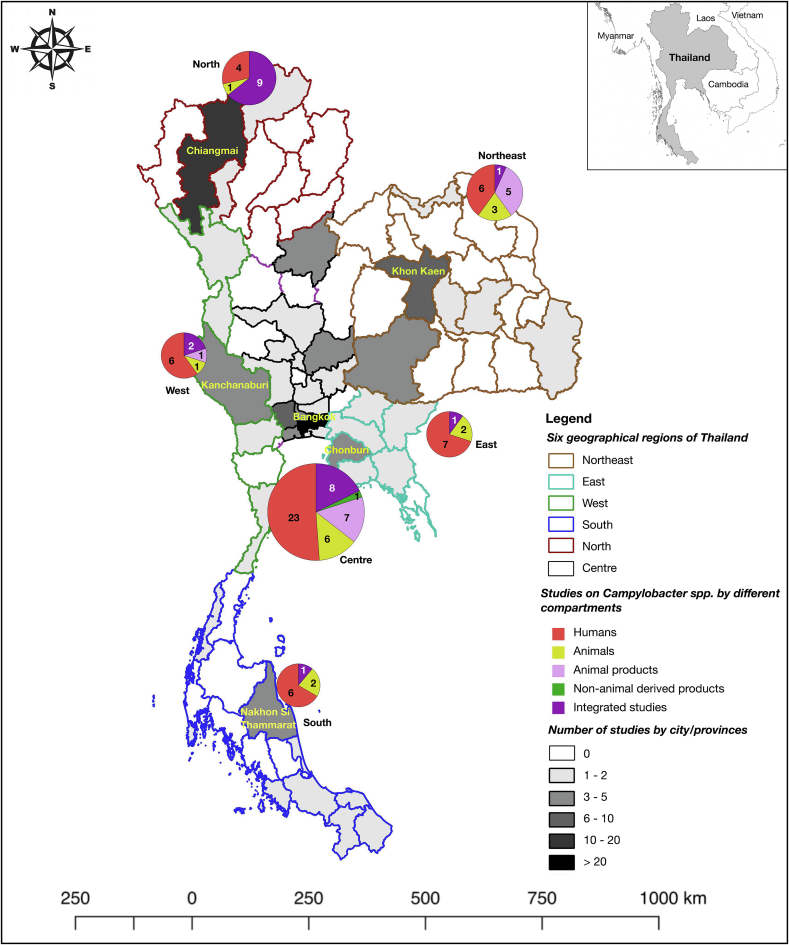
The study areas and number of selected studies on *Campylobacter* spp. across different sources of samples in Thailand. The study areas are as follows: Centre (based around Bangkok), North (based around Chiangmai), North-east (based around Khon Kaen), South (based around Nakhon Si Thammarat), West (based around Kanchanaburi), and East (based around Chonburi).

Most human studies were conducted from 1985 to 2005 (26/35 studies; 74.3 %), while studies on animals (13/13 studies; 100 %) and animal products (12/13 studies; 92.3 %) were performed from 2001 to 2023. Regarding the study area, most (32/81; 39.5 %) studies were conducted in the central region, in which 25/32 studies (78.1 %) were conducted in Bangkok. For the other regions, most studies were conducted in the following areas: North (centred around Chiangmai, 13 studies), North-east (centred around Khon Kaen, 8 studies), South (centred around Nakhon Si Thammarat, 5 studies), West (centred around Kanchanaburi, 5 studies), and East (centred around Chonburi, 4 studies). There were 14/81 studies conducted in multi-regions, including central and other parts of Thailand (Fig. 2).

**Table 1:** Characteristics of selected studies on *Campylobacter* spp. in Thailand.

Characteristics	Humans (*n* = 35) (43.2 %)	Animals (*n* = 13) (16.0 %)	Animal products (n = 13) (16.0 %)	Non-animal derived products (n = 1) (1.2 %)	Integrated studies (*n* = 19) (23.5 %)	Total (*n* = 81) (100 %)
**Sources of sample collection**						
^1^Children (diarrhea)	13 (16.0 %)	–	–	–	–	13 (16.0 %)
^2^Children (diarrhea & carriage)	3 (3.7 %)	–	–	–	–	3 (3.7 %)
^3^General population (diarrhea)	9 (11.1 %)	–	–	–	–	9 (11.1 %)
^4^General population (carriage)	2 (2.5 %)	–	–	–	–	2 (2.5 %)
^5^General population (diarrhea & carriage)	4 (4.9 %)	–	–	–	–	4 (4.9 %)
^6^Undefined age group human (diarrhea)	3 (3.7 %)	–	–	–	–	3 (3.7 %)
^7^Undefined age group (diarrhea & carriage)	1 (1.2 %)	–	–	–	–	1 (1.2 %)
^8^Chicken	–	8 (9.9 %)	–	–	–	8 (9.9 %)
^9^Duck	–	2 (2.5 %)	–	–	–	2 (2.5 %)
^10^Pig	–	1 (1.2 %)	–	–	–	1 (1.2 %)
^11^Ruminant	–	1 (1.2 %)	–	–	–	1 (1.2 %)
^12^Pig & Ruminant	–	1 (1.2 %)	–	–	–	1 (1.2 %)
^13^Chicken products	–	–	10 (12.3 %)	–	–	10 (12.3 %)
^14^Chicken, pork and ruminant products	–	–	1 (1.2 %)	–	–	1 (1.2 %)
^15^Chicken, pork, ruminant, seafood products	–	–	1 (1.2 %)	–	–	1 (1.2 %)
^16^Seafood	–	–	1 (1.2 %)	–	–	1 (1.2 %)
^17^Non-animal derived products	–	–	–	1 (1.2 %)	–	1 (1.2 %)
^18^Chicken - chicken products	–	–	–	–	4 (4.9 %)	4 (4.9 %)
^19^Children (diarrhea) - chicken	–	–	–	–	2 (2.5 %)	2 (2.5 %)
^20^Undefined age group human (diarrhea) - chicken	–	–	–	–	2 (2.5 %)	2 (2.5 %)
^21^Chicken - chicken products - environmental samples at chicken farm	–	–	–	–	2 (2.5 %)	2 (2.5 %)
^22^General population (carriage) – chicken – chicken meat	–	–	–	–	2 (2.5 %)	2 (2.5 %)
^23^Children (diarrhea) - children (carriage) - chicken - pig - ruminant - chicken meat – pork - ruminant products	–	–	–	–	2 (2.5 %)	2 (2.5 %)
^24^Children (diarrhea) - children (carriage) - chicken meat - pork - seafood	–	–	–	–	1 (1.2 %)	1 (1.2 %)
^25^Chicken - environmental samples at chicken farm	–	–	–	–	1 (1.2 %)	1 (1.2 %)
^26^Children (diarrhea) - undefined domestic animals	–	–	–	–	1 (1.2 %)	1 (1.2 %)
^27^Duck - environmental samples at duck farm	–	–	–	–	1 (1.2 %)	1 (1.2 %)
^28^Duck – duck meat - environmental samples at duck farm	–	–	–	–	1 (1.2 %)	1 (1.2 %)

**Time study conducted (year)**						
1985–1990	6 (7.4 %)	–	–	–	1 (1.2 %)	7 (8.6 %)
1991–1995	3 (3.7 %)	–	1 (1.2 %)	–		4 (4.9 %)
1996–2000	8 (9.9 %)	–	–	–	1 (1.2 %)	9 (11.1 %)
2001–2005	9 (11.1 %)	4 (4.9 %)	3 (3.7 %)	–	8 (9.9 %)	24 (29.6 %)
2006–2010	1 (1.2 %)	2 (2.5 %)	7 (8.6 %)	1 (1.2 %)	4 (4.9 %)	15 (18.5 %)
2011–2015	6 (7.4 %)	5 (6.2 %)	1 (1.2 %)	–	3 (3.7 %)	15 (18.5 %)
2016–2020	2 (2.5 %)	1 (1.2 %)	1 (1.2 %)	–	2 (2.5 %)	6 (7.4 %)
2021 - present	–	1 (1.2 %)	–	–	–	1 (1.2 %)

***Campylobacter* spp. identification method**						
Culture-based, biochemical test (Hippurate Hydrolysis Test)	15 (18.5 %)	3 (3.7 %)	5 (6.2 %)	1 (1.2 %)	4 (4.9 %)	28 (34.6 %)
PCR	9 (11.1 %)	8 (9.9 %)	8 (9.9 %)	–	13 (16.0 %)	38 (46.9 %)
Real-time PCR	2 (2.5 %)	–	–	–	–	2 (2.5 %)
WGS (Whole genome sequencing)	1 (1.2 %)	–	–	–	–	1 (1.2 %)
Not specified	8 (9.9 %)	2 (2.5 %)	–	–	2 (2.5 %)	12 (14.8 %)

**Typing methods**						
MLST (Multi-locus Sequence Typing)	1 (1.2 %)	1 (1.2 %)	1 (1.2 %)	–	3 (3.7 %)	6 (7.4 %)
MLST - *flaA* SVR (Short variable region)	–	–	–	–	2 (2.5 %)	2 (2.5 %)
PFGE (Pulsed-Field Gel Electrophoresis)	3 (3.7 %)	1 (1.2 %)	–	–	1 (1.2 %)	5 (6.2 %)
*flaA* SVR (Short variable region)	–	1 (1.2 %)	–	–	–	1 (1.2 %)
AFLP (Amplified Fragment Length Polymorphism)	–	–	–	–	1 (1.2 %)	1 (1.2 %)
*flaA* RFLP (Restriction Fragment Length Polymorphism)	–	–	–	–	1 (1.2 %)	1 (1.2 %)
Traditional method (Lior, Penner serotyping)	8 (9.9 %)	–	3 (3.7 %)	–	1 (1.2 %)	12 (14.8 %)
No application of typing methods	23 (28.4 %)	10 (12.3 %)	9 (11.1 %)	1 (1.2 %)	10 (12.3 %)	53 (65.4 %)

**Antimicrobial susceptibility testing methods**						
Disc diffusion	6 (7.4 %)	1 (1.2 %)	–	1 (1.2 %)	4 (4.9 %)	12 (14.8 %)
MIC (Agar dilution method)	3 (3.7 %)	3 (3.7 %)	2 (2.5 %)	–	4 (4.9 %)	12 (14.8 %)
MIC (Broth dilution method)	1 (1.2 %)	1 (1.2 %)	4 (4.9 %)	–	3 (3.7 %)	9 (11.1 %)
Disc diffusion - MIC (Agar dilution method)	2 (2.5 %)	–	–	–	–	2 (2.5 %)
Not applied	23 (28.4 %)	8 (9.9 %)	7 (8.6 %)	–	8 (9.9 %)	46 (56.8 %)

**Detection of ARGs and mutations**						
Yes	3 (3.7 %)	2 (2.5 %)	–	–	3 (3.7 %)	8 (9.9 %)
No	32 (39.5 %)	11 (13.6 %)	13 (16.0 %)	1 (1.2 %)	16 (19.8 %)	73 (90.1 %)

^1^ 13 studies [25,[38], [39], [40], [41], [42], [43], [44], [45], [46], [47], [48], [49]]; ^2^ 3 studies [12,14,50]; ^3^ 9 studies [[51], [52], [53], [54], [55], [56], [57], [58], [59]]; ^4^ 2 studies [60,61]; ^5^ 4 studies [11,[62], [63], [64]]; ^6^ 3 studies [[65], [66], [67]]; ^7^ 1 study [13]; ^8^ 8 studies [[68], [69], [70], [71], [72], [73], [74], [75]]; ^9^ 2 studies [76,77]; ^10^ 1 study [78]; ^11^ 1 study [79]; ^12^ 1 study [80]; ^13^ 10 studies [[81], [82], [83], [84], [85], [86], [87], [88], [89], [90]]; ^14^ 1 study [91]; ^15^ 1 study [92]; ^16^ 1 study [93]; ^17^ 1 study [94]; ^18^ 4 studies [[95], [96], [97], [98]]; ^19^ 2 studies [99,100]; ^20^ 2 studies [101,102]; ^21^ 2 studies [103,104]; ^22^ 2 studies [105,106]; ^23^ 2 studies [107,108]; ^24^ 1 study [109]; ^25^ 1 study [110]; ^26^ 1 study [111]; ^27^ 1 study [112]; ^28^ 1 study [113].

Regarding *Campylobacter* identification, PCR was the most common method, being used in 38/81 studies (46.9 %). Twenty-eight out of 81 studies (34.6 %) used typing methods, including multi-locus sequence typing (MLST) (eight studies, 9.9 %), and pulsed-field gel electrophoresis (PFGE) (five studies, 6.2 %), whereas most studies conducted before 2000 used traditional methods such as Lior and Penner serotyping. In 35/81 studies (43.2 %), *Campylobacter* isolates were investigated for their antimicrobial susceptibility. The disc diffusion test was used in 12 studies (14.8 %), while 21 studies (25.9 %) applied the minimum inhibitory concentration (MIC) method. Only 8/81 studies (9.9 %) performed genotypic testing to detect antimicrobial resistance genes (ARGs) and mutations. Full details are given in Table 1 and Supplementary Table 3.

### 3.3 Prevalence of Campylobacter spp.

Thirty-three studies (40.7 %) reporting the prevalence of *C. jejuni* and *C. coli* isolates were selected for meta-analysis. Only studies that reported data on *Campylobacter* prevalence covering at least two articles of each category were selected for meta-analysis. Among these, *C. jejuni* were reported in all studies, while *C. coli* reported in 25/33 studies (75.8 %).

**Fig. 3:**
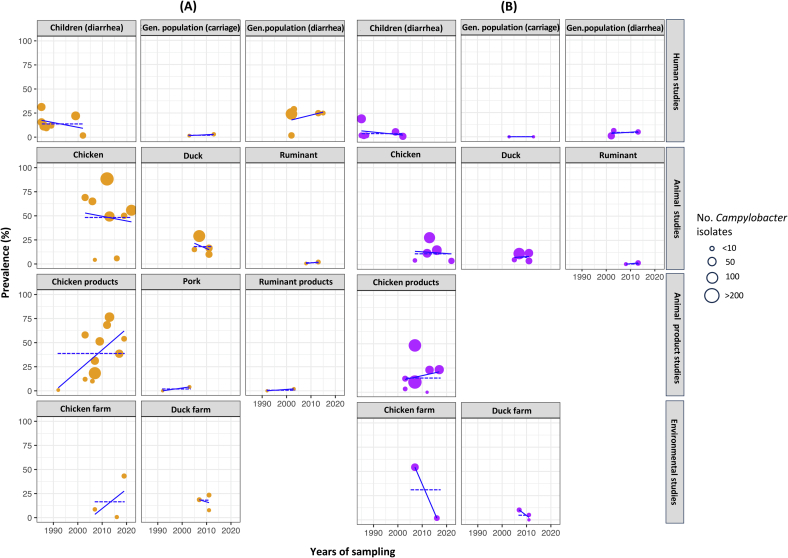
Crude prevalence of *Campylobacter* spp. detection in humans, animals, animal products and environment over time in Thailand. (A) *Campylobacter jejuni* (figures with orange scatters), (B) *Campylobacter coli* (figures with purple scatters). The solid lines correspond the linear regression directions. The dashed lines illustrate the average prevalence across studies. (For interpretation of the references to colour in this figure legend, the reader is referred to the web version of this article.)

Fifteen studies (18.5 %) on *C. jejuni* and twelve (14.8 %) on *C. coli* involved multiple sources of sample collection, yielding 84 prevalence estimates, 49 for *C. jejuni* and 35 for *C. coli*, respectively. Sensitivity analyses generally aligned with the main analyses, except in studies on children with diarrhea. Seven studies in this category initially showed no significant trends in *C. jejuni* prevalence over time. However, after removing an influential study, the trend became significant (Supplementary Table 4). Therefore, we excluded this study from our meta-analyses, resulting in six studies on *C. jejuni* in children with diarrhea, with a total of 48 prevalence estimates for *C. jejuni*. Consequently, the meta-analyses included *C. jejuni* studies from human (*n* = 13/48; 27.1 %), animal (*n* = 14/48; 29.2 %), animal products (*n* = 15/48; 31.3 %), and environmental sources (*n* = 6; 12.5 %). For *C. coli*, the studies comprised human (*n* = 11/35; 31.4 %), animal (n = 11/35; 31.4 %), animal products (*n* = 8/35; 22.9 %), and environmental sources (*n* = 5; 14.3 %). Table 2 and Fig. 3 illustrate the prevalence and meta-regression analyses of *Campylobacter* isolates across various sample sources over time in Thailand. Detailed prevalence data are provided in Supplementary Table 5**,** and forest plots are shown in Supplementary Fig. 1.

Among the various sources of sample collection in studies on *C. jejuni*, the highest prevalence was observed in chickens, with a pooled prevalence of 43.6 % (crude prevalence 48.4 %), followed by chicken products at 31.4 % (crude prevalence 38.1 %), ducks at 16.7 % (crude prevalence 17.6 %), the general population with diarrhea at 12.0 % (crude prevalence 21.0 %), and children with diarrhea at 12.0 % (crude prevalence 14.9 %). Other categories had low prevalence levels, all below 2.6 %. In environmental samples, the pooled prevalence of *C. jejuni* was 17.1 % (crude prevalence 16.6 %) at duck farms and 6.7 % (crude prevalence 17.5 %) at chicken farms. Similarly, the highest pooled prevalence was observed in samples related to chickens, with the *C. coli* prevalence levels being as follows: environmental samples from chicken farms 12.6 % (crude prevalence 17.5 %), chicken products 10.4 % (crude prevalence 13.3 %), and chicken 10.30 % (crude prevalence 8.9 %). Other categories were found with low prevalence levels, all below 7.4 %.

**Table 2:** Meta-regression analyses of *Campylobacter* spp. prevalence by different sources of sample collection over time in Thailand.

Categories	Meta-analyses					Meta-regression analyses of univariable (years)		
	No. prevalence estimates⁎	*I*^*2*^ (%)	Pooled prevalence (%)	95 % CI	p-value	*β*	95 % CI	p-value
***C. jejuni***								
**Humans**								
All human studies	13	98.2	10.4	5.3–19.2	< 0.001	−0.016	−0.08-0.05	0.593
Children (diarrhea)	6	98.2	10.7	4.2–24.8	< 0.001	−0.148	−0.22-0.07	0.006⁎
General population (diarrhea)	5	98.8	15.9	4.1–45.3	< 0.001	0.083	−0.19-0.36	0.405
General population (carriage)	2	0.0	2.6	0.0–83.9	0.560	0.065	nc	nc
**Animals**								
All animal studies	14	98.9	22.4	8.6–46.9	<0.001	0.034	−0.18-0.25	0.735
Chicken	8	98.6	43.6	16.3–75.3	<0.001	−0.015	−0.24-0.21	0.878
Duck	4	96.8	16.7	8.4–30.6	<0.001	−0.092	−0.46-0.27	0.394
Ruminant	2	40.2	1.7	0.0–36.9	0.196	0.270	nc	nc
**Animal products**								
All animal product studies	15	97.4	13.3	3.8–37.5	<0.001	0.249	0.14–0.35	<0.001
Chicken products	11	98.1	31.4	14.1–56.0	<0.001	0.167	0.06–0.28	0.007⁎
Pork	2	0.0	0.8	0.0–100.0	0.999	2.22	nc	nc
Ruminant products	2	0.0	0.5	0.0–100.0	0.999	2.16	nc	nc
**Environment**								
All environment studies	6	93.1	10.1	0.2–36.6	<0.001	−0.01	−0.41-0.39	0.953
Samples collected at chicken farm	3	97.0	6.7	0.1–91.9	<0.001	0.07	−2.80-2.94	0.809
Samples collected at duck farm	3	43.2	17.1	6.4–38.0	0.172	−0.04	−1.58-1.49	0.782
***C. coli***								
**Humans**								
All human studies	11	96.4	2.5	1.2–5.3	<0.001	−0.04	−0.12 − 0.04	0.288
Children (diarrhea)	6	97.8	3.3	1.2–9.0	<0.001	-0.04	−0.19-0.12	0.558
General population (diarrhea)	3	93.1	3.2	0.5–18.9	<0.001	0.077	−0.93-1.09	0.509
General population (carriage)	2	0.0	0.0	0.0–2.2	0.999	−0.097	nc	nc
**Animals**								
All animal studies	11	95.6	6.24	3.1–12.1	<0.001	0.029	−0.13-0.19	0.693
Chicken	5	96.0	10.3	3.7–25.3	<0.001	−0.039	−0.29-0.21	0.660
Duck	4	90.0	7.4	3.1–16.4	<0.001	−0.001	−0.49-0.49	0.992
Ruminant	2	18.3	1.4	0.0–40.1	0.269	0.233	nc	nc
**Animal products**								
Chicken products	8	97.5	10.4	3.2–28.9	<0.001	0.031	−0.24-0.30	0.786
**Environment**								
All environment studies	5	96.9	5.6	0.5–41.7	<0.001	−0.369	−0.85-0.11	0.093
Samples collected at chicken farm	2	99.2	12.6	0.0–100.0	<0.001	−0.467	nc	nc
Samples collected at duck farm	3	0.0	4.4	0.2–46.7	0.682	−0.390	−3.04-2.26	0.313

⁎ The number of prevalence estimates (*n* = 83) was from 33 studies selected for meta-analysis; *I*^*2*^: inverse variance index, *statistical significance, nc: not calculated.

The inverse variance index values were notably high for studies involving humans with diarrhea, chicken, chicken products, and ducks (all *I*^*2*^ > 90 %, *p* < 0.001), indicating substantial heterogeneity in the meta-analysis.

An assessment of *Campylobacter* prevalence trends across different sample sources from 1985 to 2002 revealed a significant decrease of 14.8 % (*p* = 0.006) in the prevalence of *C. jejuni* obtained from samples collected from children with diarrhea, while there was an increase of 16.7 % in the prevalence of *C. jejuni* in samples collected from chicken products (*p* = 0.007) during the years from 1992 to 2017. No significant trends, increasing or decreasing, were observed for other categories in both *C. jejuni* and *C. coli*.

### 3.4 Sequence type distribution of Campylobacter isolates

**Fig. 4:**
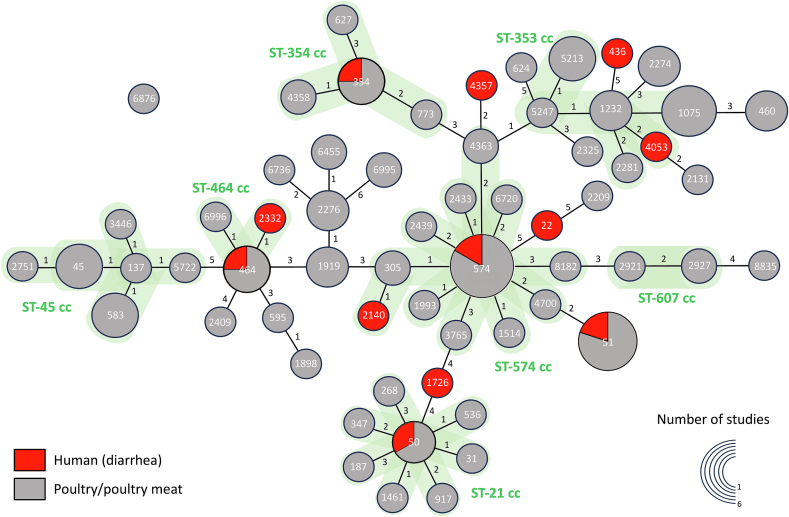
A minimum spanning tree of ST distributions of *Campylobacter jejuni* isolates from human with diarrhea and poultry/poultry meat in Thailand.

Eight studies provided data on the STs of *C. jejuni* in Thailand, which included six studies on poultry/poultry meat [72,74,89,96,97,103], one study on humans with diarrhea [67], and one study on both human with diarrhea and poultry/poultry meat [100]. None of the selected studies reported information about the STs of *C. coli*. Across the 62 STs identified, ST 574 emerged as the most prevalent (6/8 studies, 75.0 %), followed by ST 1075 and ST 51 (5/8 studies, 62.5 %), and ST 5213, ST 354, ST 45, ST 583, and ST 464 were documented in 4/8 studies (50.0 %). The ST-574 clonal complex (CC) has the highest number of STs (12 STs), followed by the ST-21 CC with 8 STs and the ST-45 CC with 6 STs. Eleven STs were undefined in any CCs. Seven STs were exclusively reported in humans, including ST 22, ST 436, ST 1726, ST 2140, ST 2332, ST 4053, and ST 4357. Notably, five STs, including ST 50, ST 51, ST 354, ST 464, and ST 574, were reported in both humans and poultry **(**Fig. 4 and Supplementary Table 6).

### 3.5 Phenotypic antimicrobial resistance

**Fig. 5:**
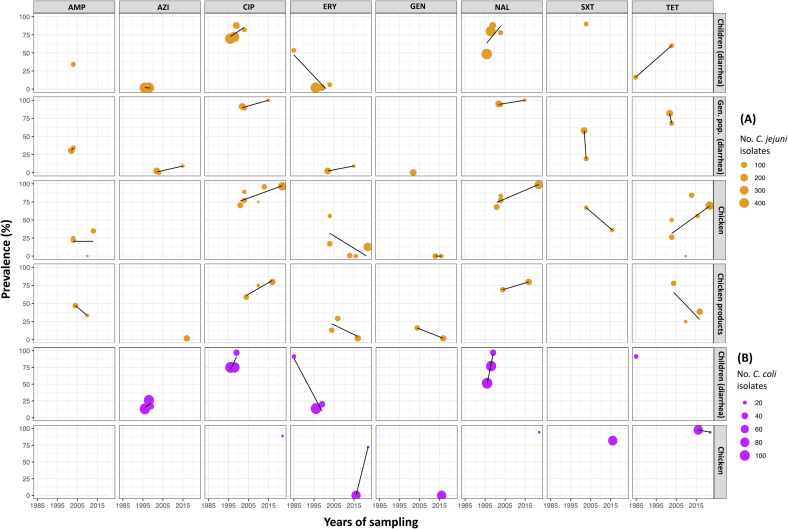
Crude prevalence of AMR in *C. jejuni* (A – in orange) *and C. coli* (B – in purple) isolates from different sources of sample collection over time in Thailand. AMP: Ampicillin; AZI: Azithromycin; CIP: Ciprofloxacin; ERY: Erythromycin; GEN: Gentamicin; NAL: Nalidixic acid; TET: Tetracycline; SXT: Sulfamethoxazole – Trimethoprim. The lines correspond the linear regression directions. (For interpretation of the references to colour in this figure legend, the reader is referred to the web version of this article.)

Only 20 studies (24.7 %) with *Campylobacter* isolates from sample sources obtained from children, general population with diarrhea, chicken and chicken products met the criteria for sufficient data to be used in meta-analyses (Fig. 5, Table 3 and Supplementary Table 7). The AMR prevalence of *Campylobacter* spp. against eight common antimicrobials, including ciprofloxacin (CIP), nalidixic acid (NAL), erythromycin (ERY), azithromycin (AZI), ampicillin (AMP), gentamicin (GEN), tetracycline (TET), and sulfamethoxazole-trimethoprim (SXT) were examined. Our sensitivity analyses found that removing influential studies resulted in non-significant outcomes in the meta-analyses due to the limited number of studies. Therefore, to preserve statistical power and significance, we included all studies (*n* = 20), both with and without assumptions, in our final analyses (Supplementary Table 8).

The highest AMR prevalence in *Campylobacter* spp. was observed for quinolone antimicrobials across all categories. In children with diarrhea, *C. jejuni* isolates were resistant to CIP and NAL at 77.9 % and 75.4 %, respectively, while for *C. coli*, the resistance levels were 83.3 % and 80.9 %. In the general population age group, resistance to CIP and NAL was 91.3 % and 94.8 %, respectively. In isolates related to chicken sources, the resistance to CIP and NAL of *C. jejuni* was 88.7 % and 87.3 % in chicken, and 71.6 % and 75.6 % in chicken products, respectively. For the macrolide antimicrobials, *C. jejuni* isolates showed high susceptibility to ERY and AZI, with resistance levels below 6.8 % across all human-related categories and 9.9 % in chicken-related categories. However, *C. coli* from children with diarrhea exhibited resistance levels of 40.9 % to ERY and 18.5 % to AZI. The AMR levels of *C. jejuni* to TET ranged from 35.4 % to 77.2 % across all categories, while *C. coli* resistance was only observed in chickens at 97.0 %. For AMP and SXT, the levels of AMR prevalence were recorded only for *C. jejuni*, and ranged from 26.5 % to 45.0 % for AMP, and 37.4 % to 51.3 % for SXT, respectively. Only chicken studies reported the prevalence of resistance to GEN in *Campylobacter* isolates, with the prevalence at 5.8 % in chicken products and 0.0 % in chickens (Supplementary Fig. 2).

The AMR levels of *C. jejuni* isolates to (fluoro)quinolones showed an increasing trend in samples collected from chickens between 1985 and 2023, with an increase of 11.9 % for CIP (*p* = 0.004) and 16.1 % for NAL (*p* = 0.027). No significant differences in AMR trends were observed for other categories or antimicrobials among *Campylobacter* isolates.

**Table 3:** AMR prevalence and trends of *C. jejuni* and *C. coli* in humans with diarrhea or in chickens over time in Thailand.

Meta-analysis	AMP	AZI	CIP	ERY	GEN	NAL	SXT	TET
***C. jejuni***								
**Children (diarrhea) (n)**	**1**	**3**	**4**	**4**	**0**	**4**	**1**	**2**
Pooled prevalence (%)	nc	1.7	77.9	6.8	nc	75.4	Nc	35.4
95 % CI	nc	0.6–4.7	62.2–88.3	0.5–53.9	nc	46.9–91.4	Nc	0.0–99.9
*β* (regression coefficient)	nc	−0.061	0.138	−0.213	nc	0.174	Nc	0.114
95 % CI	nc	−2.68-2.56	−0.21-0.49	−0.55-0.12	nc	−0.40-0.749	Nc	nc
*p*-value	nc	0.817	0.230	0.110	nc	0.323	Nc	nc
**General population (diarrhea) (n)**	**2**	**3**	**3**	**2**	**0**	**3**	**2**	**2**
Pooled prevalence (%)	31.4	2.0	91.3	2.7	nc	94.9	37.4	77.2
95 % CI	5.8–77.4	0.2–15.5	77.9–96.9	0.0–94.5	nc	82.2–98.7	0.0–99.9	10.0–99.0
*β* (regression coefficient)	0.165	0.134	0.191	0.116	nc	0.183	−1.762	−0.751
95 % CI	nc	−1.09-1.35	−2.80-3.18	nc	nc	−3.58-3.95	nc	nc
*p*-value	nc	0.396	0.566	nc	nc	0.649	nc	nc
**Chicken (n)**	**4**	**0**	**6**	**5**	**2**	**4**	**2**	**6**
Pooled prevalence (%)	26.5	nc	88.7	5.3	0.0	87.3	51.3	52.8
95 % CI	15.8–41.0	nc	71.3–96.1	0.2–67.7	0.0–100.0	44.7–98.3	0.6–99.5	25.5–78.5
*β* (regression coefficient)	0.058	nc	0.119	−0.147	0.249	0.161	−0.089	0.081
95 % CI	−0.09-0.21	nc	0.07–0.17	−0.56-0.26	nc	0.05–0.28	nc	−0.05-0.21
*p*-value	0.242	nc	0.004	0.336	nc	0.027	nc	0.155
**Chicken meat (n)**	**2**	**1**	**3**	**3**	**2**	**2**	**0**	**3**
Pooled prevalence (%)	45.0	nc	71.6	9.9	5.8	75.6	nc	49.5
95 % CI	4.5–93.4	nc	40.2–90.4	0.4–73.7	0.0–99.9	19.3–97.6	nc	7.4–92.3
*β* (regression coefficient)	−0.096	nc	0.078	−0.200	−0.183	0.044	nc	−0.125
95 % CI	nc	nc	−0.25-0.41	−1.36-0.96	nc	nc	nc	−0.45-0.21
*p*-value	nc	nc	0.206	0.273	nc	nc	nc	0.131
***C. coli***								
**Children (diarrhea) (n)**	**0**	**3**	**3**	**3**	**0**	**3**	**0**	**1**
Pooled prevalence (%)	nc	18.5	83.8	40.9	nc	80.9	nc	nc
95 % CI	nc	7.3–39.6	32.5–98.2	0.6–98.8	nc	11.7–99.3	nc	nc
*β* (regression coefficient)	nc	0.233	0.472	−0.302	nc	0.707	nc	nc
95 % CI	nc	−1.57-2.04	−3.96-4.90	−1.25-0.65	nc	−0.84-2.26	nc	nc
*p*-value	nc	0.348	0.405	0.154	nc	0.109	nc	nc
**Chicken (n)**	**0**	**0**	**1**	**2**	**1**	**1**	**1**	**2**
Pooled prevalence (%)	nc	nc	nc	2.5	nc	nc	nc	97.0
95 % CI	nc	nc	nc	0.0–100.0	nc	nc	nc	1.9–100.0
*β* (regression coefficient)	nc	nc	nc	4.679	nc	nc	nc	−0.143
95 % CI	nc	nc	nc	nc	nc	nc	nc	nc
*p*-value	nc	nc	nc	nc	nc	nc	nc	nc

n: number of studies. *β*: regression coefficient of independent variable ‘year of sampling’ for the univariable meta-regression models. nc: not calculated due to insufficient studies for meta-analysis. AMP: Ampicillin; AZI: Azithromycin; CIP: Ciprofloxacin; ERY: Erythromycin; GEN: Gentamicin; NAL: Nalidixic acid; TET: Tetracycline; SXT: Sulfamethoxazole – Trimethoprim.

### 3.6 Genotypic antimicrobial resistance

Of 81 studies, 8 articles (9.9 %) detected the ARGs and mutations among *Campylobacter* isolates, including studies on humans with diarrhea (*n* = 3) [42,44,55]; poultry (n = 3) [71,106,110]; pigs (*n* = 1) [78]; and study on both humans with diarrhea and chickens (n = 1) [101]. A total of 13 ARGs/mutations conferring against for 4 antimicrobial classes were identified (Table 4).

**Table 4:**
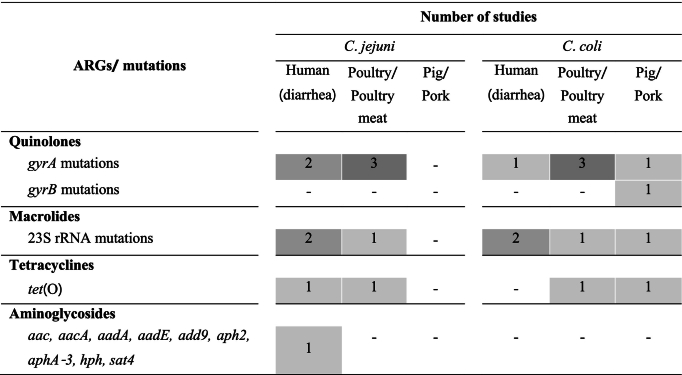
Number of Thai studies that reported genotypic antimicrobial resistance in *Campylobacter* spp.

The presence of the threonine-86-isoleucine (Thr-86-Ile) mutation in the DNA gyrase gene (*gyrA*) is recognized as being linked to resistance against quinolones. For example, 70/70 quinolone-resistant *C. jejuni* isolates from humans and 69/69 isolates from chickens harbored the *gyrA* mutant gene [101]. In the central region, a study on broilers revealed that 100 % of both *C. jejuni* and *C. coli* isolates resistant to quinolones possessed *gyrA* mutations [110]. In northern Thailand, an investigation on chicken meat demonstrated a highly significant association of 59/59 isolates between CIP resistance and the Thr-86-Ile mutation among *C. jejuni* isolates [106]. Similarly, in southern Thailand, a study observed concordance in 234/275 *Campylobacter* isolates between CIP resistance and *gyrA* mutant genes [71]. In the case of pigs, 59/60 quinolone-resistant *C. coli* isolates were found to contain *gyrA* mutant genes, while 5/60*C. coli* isolates possessed *gyrB* mutant gene [78].

The prevalent mechanism conferring resistance to macrolides in *Campylobacter* involves a point mutation in the 23S rRNA gene. A study conducted on pigs found that 34/44 ERY-resistant isolates were associated with point mutations in the 23S rRNA gene, with the A2230G mutation present in 55 % of these isolates [78]. In the case of chickens, only 7.6 % of the isolates had the A2074C point mutation in the 23S rRNA gene, and no significant association was observed between macrolides and A2074C mutation in the 23S rRNA genes [71]. A study on humans with diarrhea also reported only 1 out of 9 macrolide-resistant *Campylobacter* isolates exhibited the A2075G mutation in the 23S rRNA gene [42].

The plasmid-encoded *tet*(O) gene is a characteristic associated with TET resistance in *Campylobacter* isolates. A strong correlation between TET-resistant *Campylobacter* isolates and the presence of *tet*(O) genes has been recognized. In chickens, out of 204 isolates, 180 TET resistant isolates harbored *tet*(O) [71], while in a pig study 65 out of 67 TET resistant isolates showed this association [78]. Furthermore, another study on *C. jejuni* isolates from humans with diarrhea not only identified the plasmid-encoded *tet*(O) gene in association with TET resistance but also revealed the genes (i.e. *aac, aacA, aadA, aadE, add9, aph2, aphA-3, hph, sat4*) associated with aminoglycoside resistance were plasmid-encoded [55].

### 3.7 Publication bias assessment

The contour-enhanced funnel plots illustrate asymmetry among the selected articles in the meta-analysis of *Campylobacter* prevalence (*n* = 60 studies) and the AMR prevalence of *Campylobacter* spp. (*n* = 20 studies) (Supplementary Fig. 3). The scattered points representing selected articles are unevenly distributed and situated far from the pooled effect size (vertical line). Most scatters were plotted within the shaded regions of *p* < 0.05 and *p* < 0.01, indicating the significant asymmetry of selected articles in the meta-analysis.

The asymmetry observed in the funnel plots was further supported by the results of Egger's regression tests. The intercepts (*βo*) of meta-analysis involving *Campylobacter* spp. in humans and resistance to AMP, AZI, CIP, ERY, GEN, and NAL differ from zero (all *βo* either < −1.232 or > 1.942). Conversely, in studies reporting data on *Campylobacter* prevalence in animals (*βo* = −0.620), and AMR prevalence to SXT (*βo* = 0.037) and TET (*βo* = 0.007), although the intercept of Egger's regression tests was close to zero, no significance was detected (all *p* ≥ 0.763).

Moreover, substantial heterogeneity was observed, indicating publication bias among these selected studies. The inverse variance index (*I*^*2*^) values were notably high in studies involving humans, animals, and resistance to CIP, NAL, ERY, AZI, TET, and SXT (all *I*^*2*^ > 83.4 %, all *p* < 0.001). For studies involving resistance to GEN and AMP, non-significance heterogeneity was detected (*I*^*2*^ = 50.7 % and 29.4 %, respectively) (all *p* ≥ 0.175).

## 4 Discussion

This systematic review and meta-analysis represents the first comprehensive investigation of *Campylobacter* spp. in Thailand following a One Health approach. Food-borne pathogens are an important cause of morbidity worldwide, and *Campylobacter* spp. are recognized as the main bacteria causing food-borne diarrheal disease [114]. Our study strategically categorized *Campylobacter* isolates based on various sources, including humans, animals, animal products and environmental samples. This study approach aimed not only to minimize the information bias but to provide a detailed insight of the prevalence and AMR trends of *Campylobacter* spp. isolated from various sources in Thailand.

Poultry, especially chickens, are known to be the main reservoir of *Campylobacter* spp. Findings from our studies highlight the high prevalence of *Campylobacter* isolated from chicken-derived samples. These findings align with a recent One Health review in another LMIC, Ethiopia, which reported higher prevalence rates in animals, especially chickens, compared to humans [115]. The high prevalence of *Campylobacter* observed in chicken studies likely reflects the nature of intensive poultry production systems [116]. Environmental factors and climate change also contribute to the high prevalence of *Campylobacter* spp. [117]. Additionally, our review revealed that, compared to human studies, recent animal studies have shown higher prevalence levels, which may be attributed to the improved sensitivity of *Campylobacter* detection tests, such as the widespread adoption of PCR in recent animal studies.

Our review identified a general decrease in *Campylobacter* prevalence among Thai people, particularly children, from 1985 to 2002. This decline could be attributed to Thailand's consumer education programs on basic food safety [118]. However, from 1992 to 2017, we observed an increasing trend in *C. jejuni* prevalence in chicken products, suggesting contamination during processing. A study in the USA involving 8003 campylobacteriosis cases from 1998 to 2006 reported that 17 % of *Campylobacter* outbreaks were associated with chicken products [119]. This underscores the risk of *Campylobacter* spp. as a causative agent of gastroenteritis in humans. Therefore, continuous monitoring and implementing preventive measures within food chain systems, particularly during chicken product processing, are crucial. Additionally, food safety programs for food-service workers are essential to mitigate the impact of *Campylobacter* infections effectively.

The genetic diversity of STs among *C. jejuni* isolates from poultry and diarrhea humans was shown in our review. A number of STs associated with the ST-21, ST-443, ST-354, ST-464, and ST-574 clonal complexes (CCs) were reported in isolates from both human diarrhea cases and poultry. A previous review on the MLST profiles of *C. jejuni* isolates also reported genetic diversity among isolates with CCs such as ST-21, ST-45, ST-257, ST-48, ST-61, ST-206, ST-628, ST-177 being reported in multiple host sources including humans and various animal species [120]. In Thailand, our review found no reports about MLST profiles of *Campylobacter* isolates that were obtained from other animal species than poultry. However, based on the findings of previous studies in other countries, it is likely that *Campylobacter* isolates obtained from different animal species may exhibit distinctly different dominant MLST profiles. For example, in ruminants, CCs ST-61, ST-48, and ST-42 were the most common among *Campylobacter* isolates [121]. In pigs, a higher prevalence of *C. coli* was found compared to *C. jejuni*, with the most common CCs being ST-828 [122,123]. Also, CCs ST-177 and ST-682 were common among wild starlings [124]. Since MLST is a crucial tool applied to understand the genetic diversity, molecular epidemiology, and disease surveillance of microorganisms, it is suggested that further studies on the MLST profiles of *C. coli* isolates from other animal species are needed in order to have a comprehensive picture of genetic diversity and the evolution of *Campylobacter* spp. in Thailand.

A high prevalence of AMR levels against (fluoro)quinolone antimicrobials, including CIP and NAL, among *Campylobacter* isolates from humans and chicken was observed in our review. These findings align with the recent studies on the *Campylobacter* burden, which have reported the increasingly widespread occurrence of fluoroquinolone and tetracycline resistance in isolates from both humans [125], and farm animals [126]. Our study identified a low prevalence of macrolide-resistant *C. jejuni* and *C. coli* isolates in Thai humans and chickens. However, substantial resistance (>82 %) to ERY and AZI was reported among *Campylobacter* isolates from Thai swine [107]. This finding was not presented in our results due to insufficient studies for meta-analysis of AMR in *Campylobacter* isolates from pigs/pork. In contrast, a study in Vietnam, another LMIC in Southeast Asia, documented 100 % resistance to erythromycin among *Campylobacter* isolates from chickens, ducks, and pigs [122]. These findings highlight the complexity of *Campylobacter* AMR patterns, shaped by variations in animal populations, environmental factors, and geographic regions. As well, the increased AMR level of *Campylobacter* spp. in chickens revealed in this review implies a potential link with antimicrobial use practices in chicken production systems. In Thailand, fluoroquinolones, among the highest-priority critically important antimicrobials, were the most commonly used antimicrobials in humans and food-producing animals [22]. Based on the Thailand National Strategic Plan on the regulation of antimicrobial distribution on AMR [127], legislative and regulatory measures are recommended to restrict the use of fluoroquinolones and macrolides in animal production systems in Thailand. Besides, further investigations are also suggested to explore the association between antimicrobial use in animal production systems and AMR [16].

Our review uncovered a connection between resistance to (fluoro)quinolones and TET and the presence of *gryA* mutations and *tet(*O*)* genes in *Campylobacter* spp. isolates in Thailand. However, there was no consistent agreement on macrolides and their related-ARGs across the reviewed studies. Understanding the relationship between phenotypic and genotypic resistance in *Campylobacter* isolates is still challenging [128]. The observed phenotypic resistance can stem from various sources that include low-level resistance gene expression, bacterial mechanisms controlling gene expression [129], and non-targeted resistance involving efflux pumps [130]. Additionally, the methods used for genotypic resistance testing may have constraints affecting the correlation with the observed phenotypic resistance. Most of the reviewed studies utilized band-based genotyping and not sequence-based methods. It is crucial to utilize both phenotypic and genotypic approaches, with a preference for sequence-based genotyping, to detect and monitor the AMR of *Campylobacter* spp. effectively.

Our study had several limitations. Firstly, while our review sought to investigate the prevalence and AMR of *Campylobacter* isolates across humans, animals, animal products, and the environment, the AMR prevalence was exclusively observed in isolates obtained from humans with diarrhea and from chickens but did not cover other animal species *(*e.g. ducks and pigs*)*. Secondly, although sensitivity analyses were conducted to assess the impact of studies using assumed sampling years, the meta-analysis included only a small number of studies due to the diverse classification of sample sources. Therefore, to ensure adequate studies, we accepted these assumptions in some parts of our meta-analysis. Lastly, despite efforts to minimize biases through study selection, data extraction, and publication bias assessment, the comprehensive nature of our review and the extensive study period under a One Health approach unavoidably introduced high heterogeneity among selected studies. The observed high heterogeneity may be influenced by publication bias stemming from the impact of small studies with limited sample size possibly posing a challenge to the representativeness of the AMR situation of *Campylobacter* spp. throughout the entire country. Nevertheless, within these acknowledged limitations, our study provides valuable insights into *Campylobacter* prevalence and trends in AMR in these pathogens in Thailand under a One Health approach, offering a potential model for application in other LMICs.

## 5 Conclusion

Our review highlighted the high prevalence of *Campylobacter* spp. isolates in both poultry and humans in Thailand. Additionally, our study evidenced the high and increasing incidence of AMR among *Campylobacter* spp. isolates from poultry and humans in the country over time, particularly to quinolone antimicrobials. Given that Thailand has an ongoing AMR surveillance system aligned with the strategies outlined in the National Strategic Plan, our findings emphasize the need for establishing comprehensive surveillance systems encompassing key bacterial species, including *Campylobacter* spp., under a One Health approach. Achieving this requires coordinated efforts among stakeholders, improved source attribution to specific sources, and systematic monitoring of intervention strategies. Furthermore, public education campaigns are crucial for raising awareness about *Campylobacter* infections to tackle the growing AMR challenges in Thailand.

The following are the supplementary data related to this article.

Supplementary Fig. 1 Forest plots of prevalence of *C. jejuni* and *C. coli* by different sources of sample collection. Supplementary Fig. 1

Supplementary Fig. 2 Forest plots of AMR prevalence of *C. jejuni* and *C. coli* by different sources of sample collection. Supplementary Fig. 2

Supplementary Fig. 3 Contour-enhanced funnel plots and Egger's tests used for publication bias assessment. Supplementary Fig. 3

Supplementary Table 1 The PRISMA 2020 item checklist. Supplementary Table 1

Supplementary Table 2 JBI Critical Appraisal Checklist for minimizing the bias selection of included studies. Supplementary Table 2

Supplementary Table 3 Data extraction of included studies. Supplementary Table 3

Supplementary Table 4 Sensitivity analysis reports the results of *Campylobacter* prevalence of selected studies. Supplementary Table 4

Supplementary Table 5 Prevalence of *Campylobacter* spp. isolates obtained from different sources of sample collection. Supplementary Table 5

Supplementary Table 6 The sequence types distribution of *C. jejuni* in Thailand. Supplementary Table 6

Supplementary Table 7 The prevalence of phenotypic antimicrobial resistance of *C. jejuni* and *C. coli* in Thailand; Supplementary Table 7

Supplementary Table 8 Sensitivity analysis reports the results of AMR prevalence of *Campylobacter* spp. in included studies. Supplementary Table 8

## Funding

This work has been supported by Thailand Science Research and 10.13039/100012774Innovation Fund (Contract No. FRB650082/0227) and also supported by 10.13039/501100010034Walailak University under the international research collaboration Scheme (Contract Number WU-CIA-02704/2024) awarded to Assoc. Prof. Dr. Thotsapol Thomrongsuwannakij.

## Declaration of generative AI in scientific writing

During the preparation of this work, the authors used the SCISPACE and Grammarly tools to improve the readability and language of the manuscript. After using this tool, the authors reviewed and edited the content as needed and took full responsibility for the content of the published articles.

## CRediT authorship contribution statement

**Doan Hoang Phu:** Writing – original draft, Methodology, Investigation, Formal analysis, Data curation, Conceptualization. **Tuempong Wongtawan:** Writing – review & editing, Validation, Conceptualization. **Truong Thanh Nam:** Methodology, Formal analysis. **Dinh Bao Truong:** Formal analysis, Data curation. **Naparat Suttidate:** Writing – review & editing, Validation. **Juan Carrique-Mas:** Writing – review & editing, Conceptualization. **Niwat Chansiripornchai:** Writing – review & editing. **Conny Turni:** Writing – review & editing, Data curation. **Patrick J. Blackall:** Writing – review & editing, Data curation. **Thotsapol Thomrongsuwannakij:** Writing – review & editing, Supervision, Project administration, Methodology, Funding acquisition, Conceptualization.

## Declaration of competing interest

The authors declare no conflicts of interest.

## Data Availability

Data will be made available on request.
