# Supplementary material for: Prevalence and antimicrobial resistance of Campylobacter jejuni and Campylobacter coli over time in Thailand under a One Health approach: A systematic review and meta-analysis
Source: One Health. 2025 Jan 10;20:100965. doi: 10.1016/j.onehlt.2025.100965 (PMC11782884; doi:10.1016/j.onehlt.2025.100965)
Supplement: Supplementary Fig. 1 — Forest plots of prevalence of C. jejuni and C. coli by different sources of sample collection. [file mmc1.docx]

**Supplementary Figure 1**: Forest plots of prevalence of *C. jejuni* and *C. coli* by different sources of sample collection.

***A. Campylobacter jejuni***

***All human studies (n=13)***

***
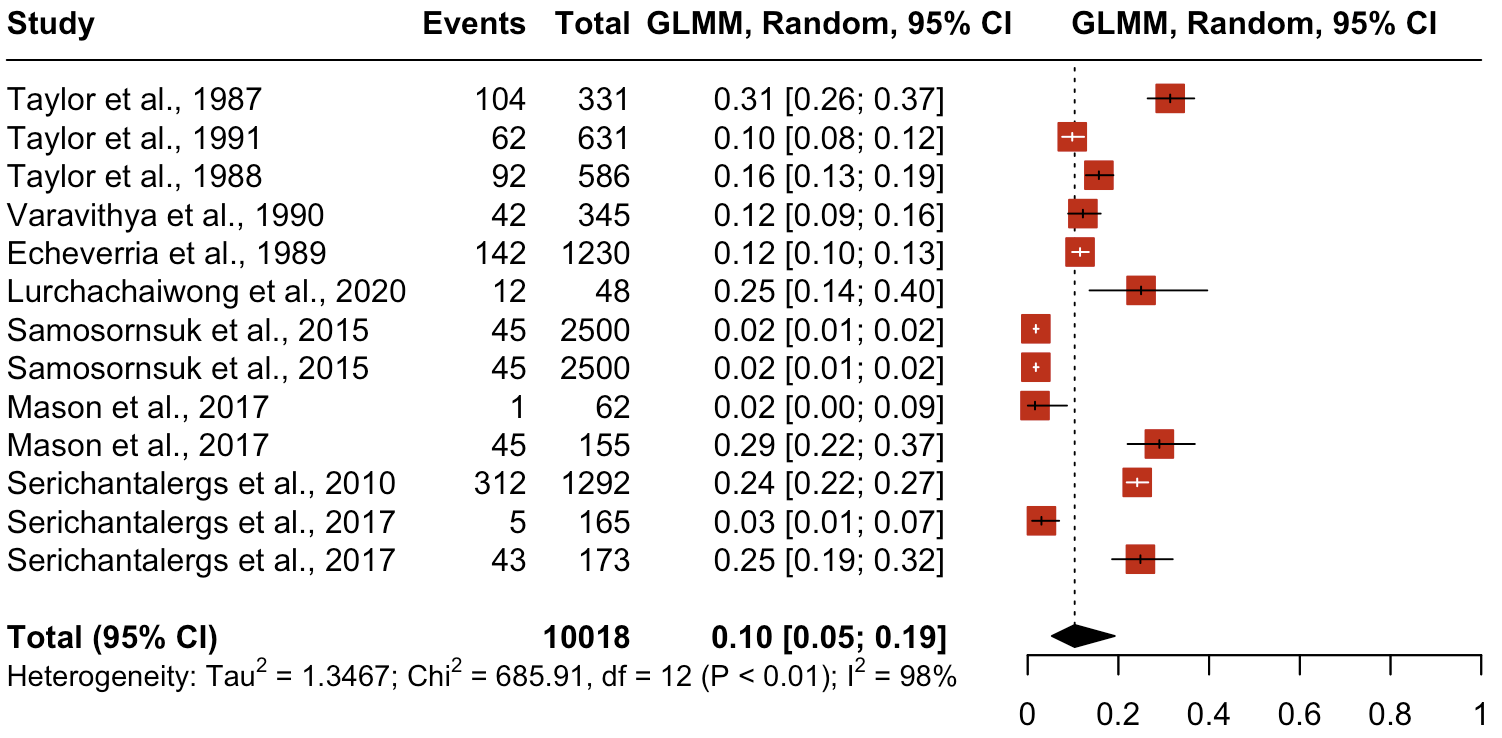
***

***Children (diarrhea) (n=6)***

*
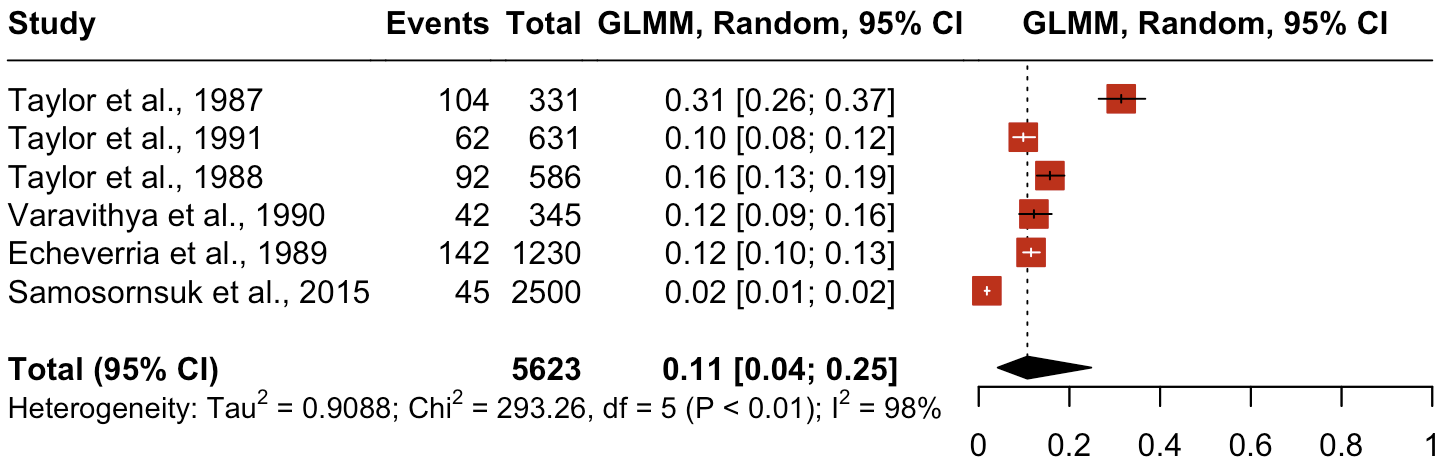
*

***General population (diarrhea) (n=5)***

*
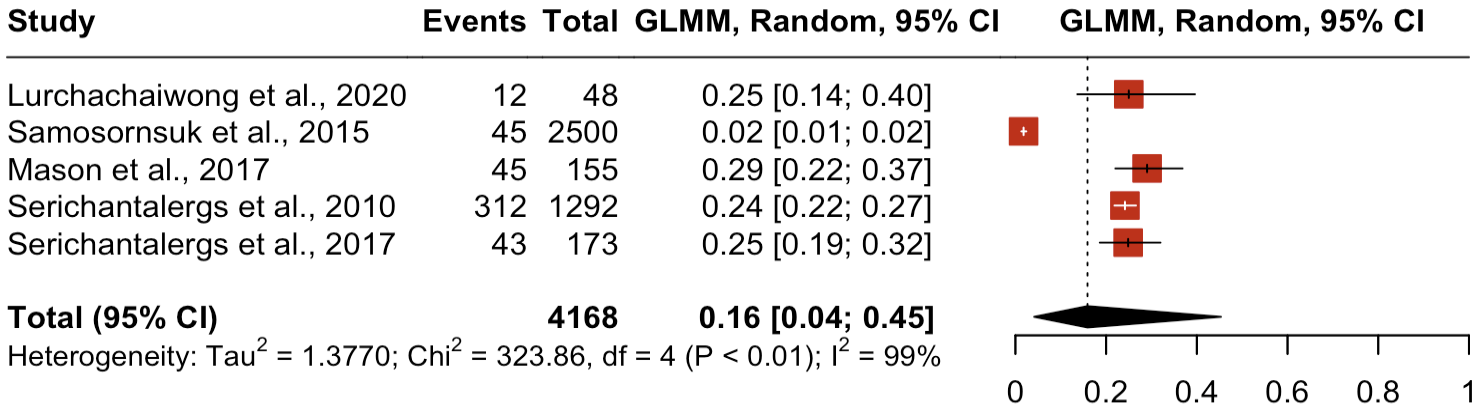
*

***General population (carriage) (n=2)***

*
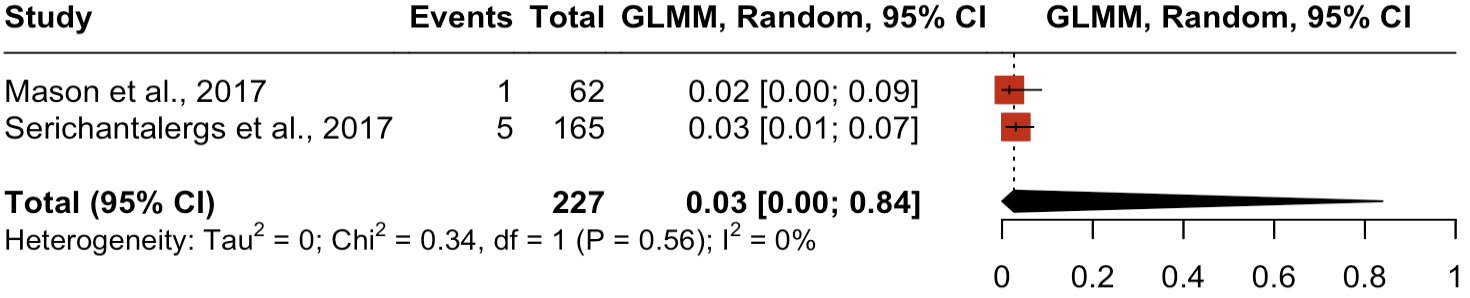
*

***All animal studies (n=14)***

*
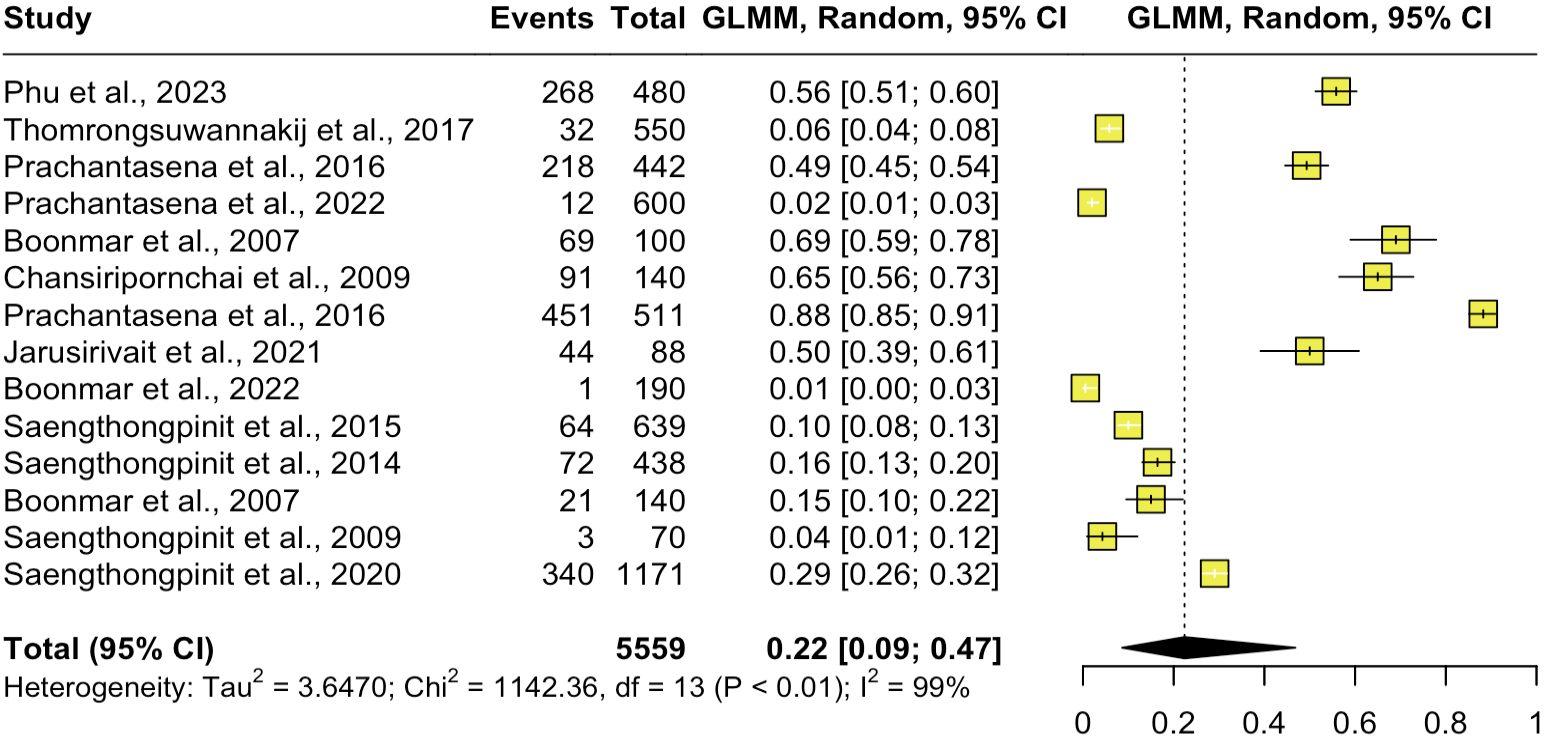
*

***Chicken (n=8)***

*
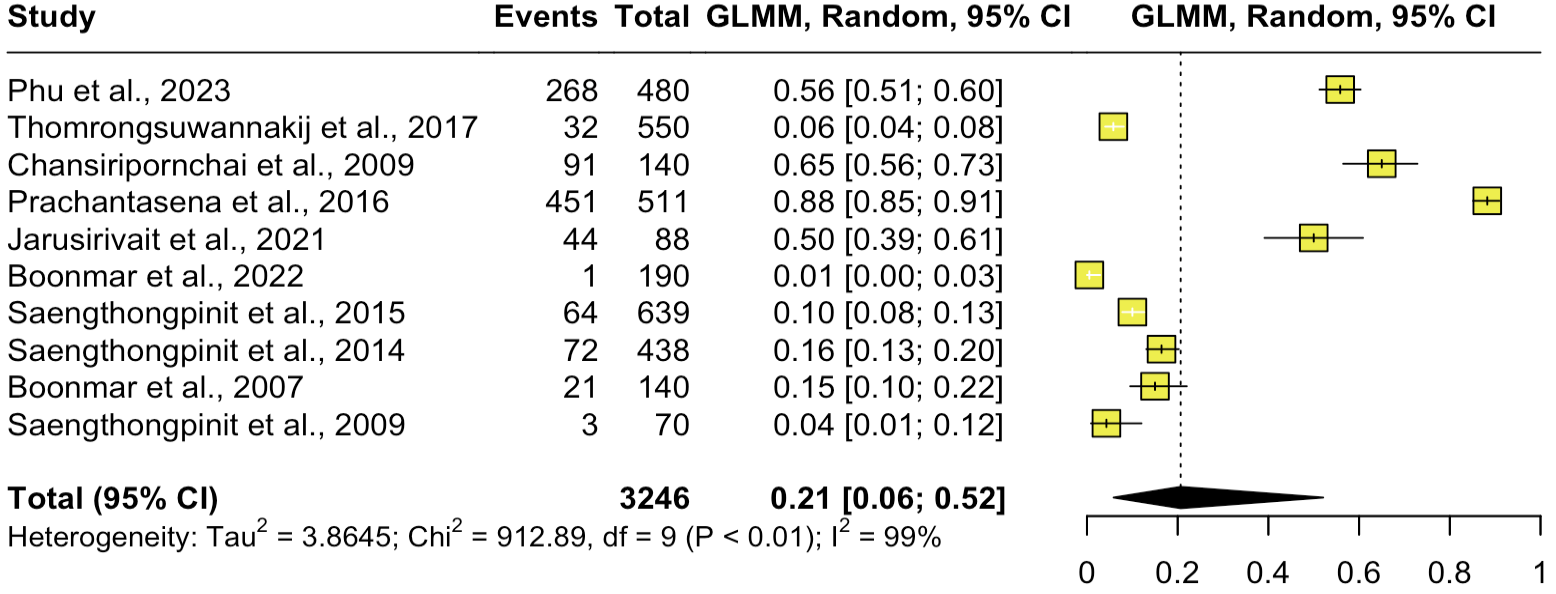
*

***Duck (n=4)***

*
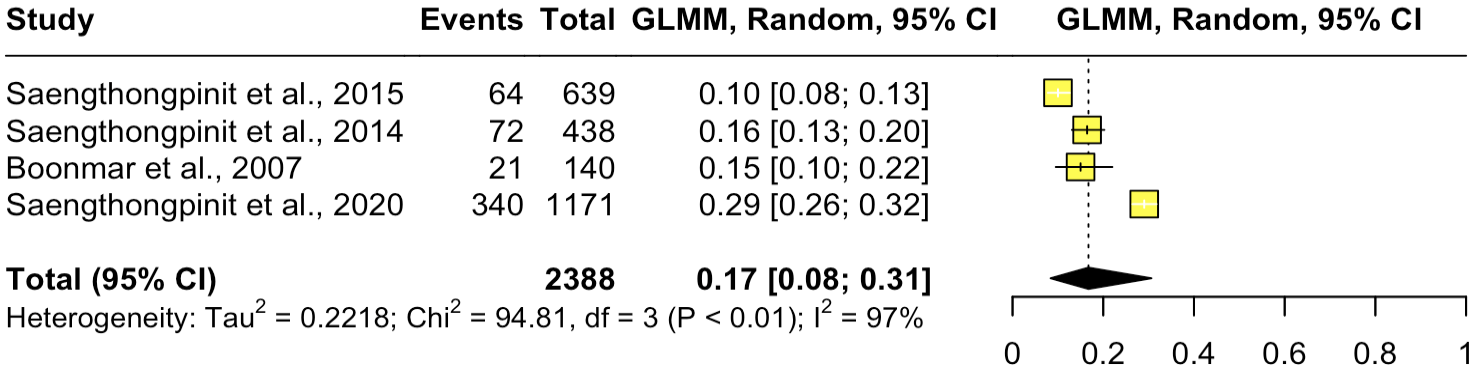
*

***Ruminant (n=2)***

*
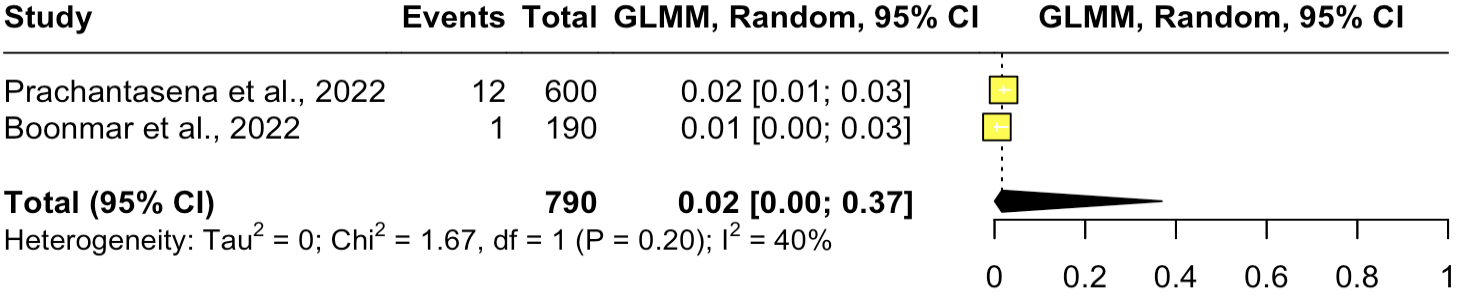
*

***All animal product studies (n=15)***

*
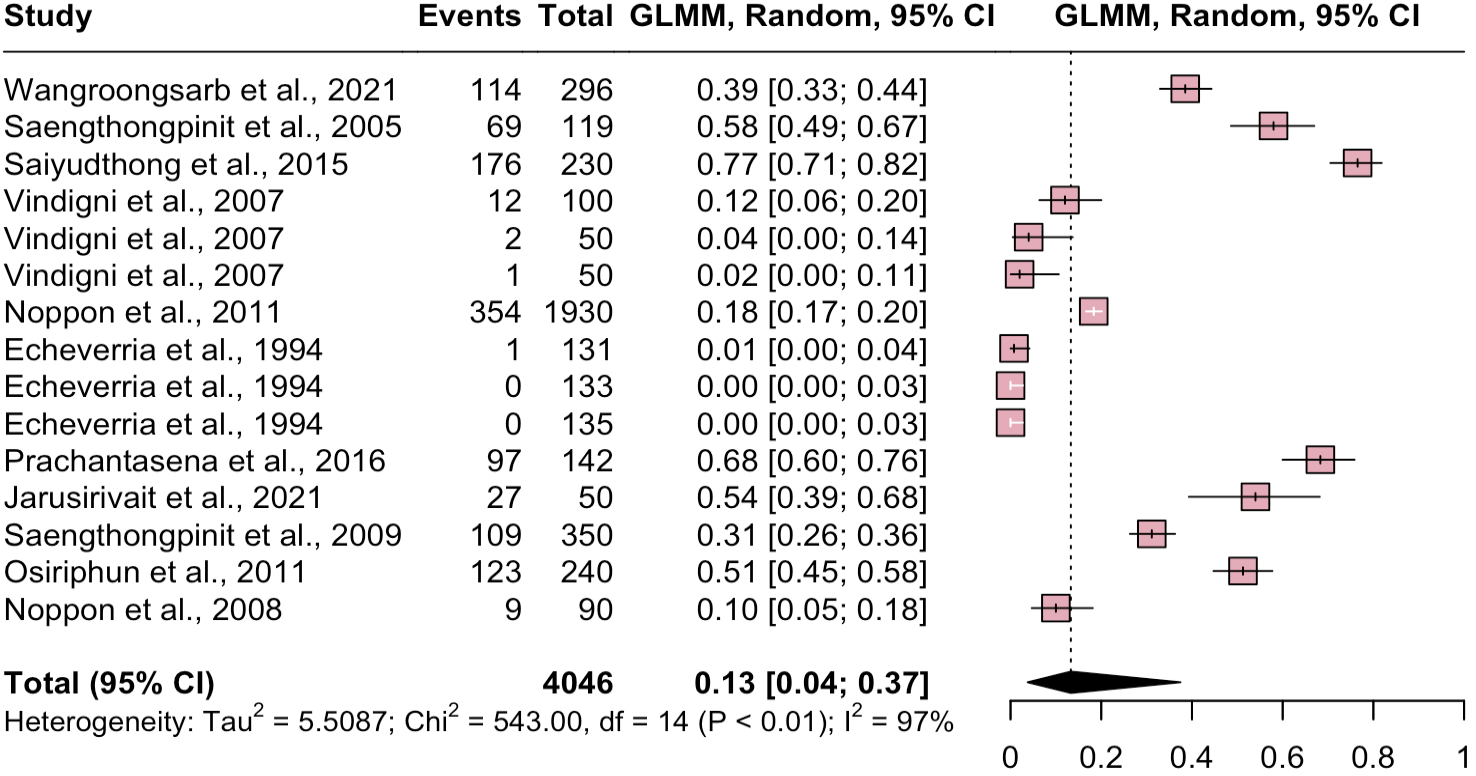
*

***Chicken products (n=11)***

*
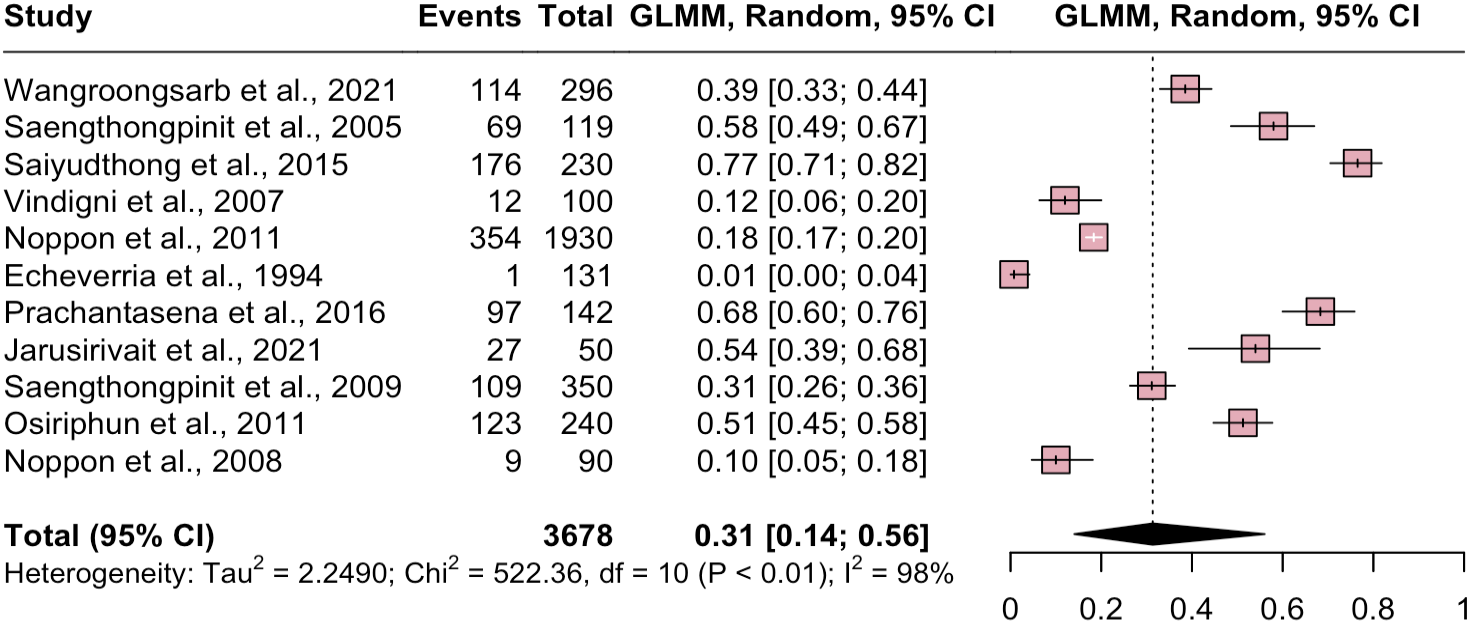
*

***Pork (n=2)***

*
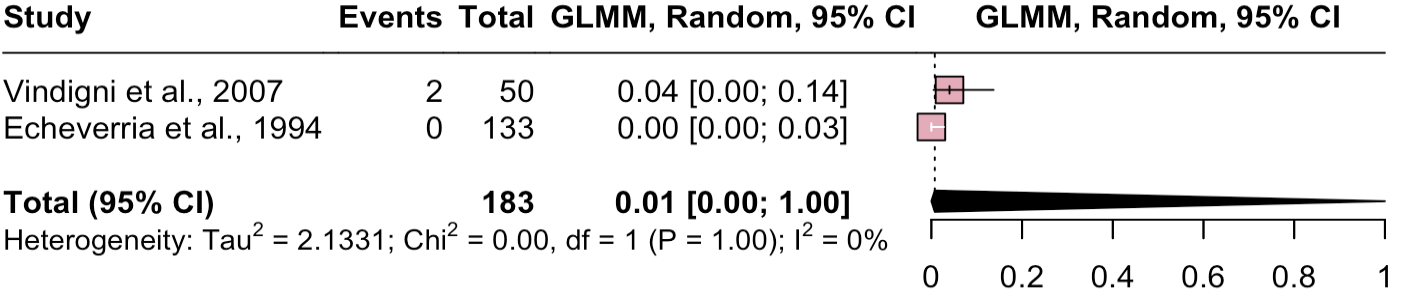
*

***Ruminant products (n=2)***

*
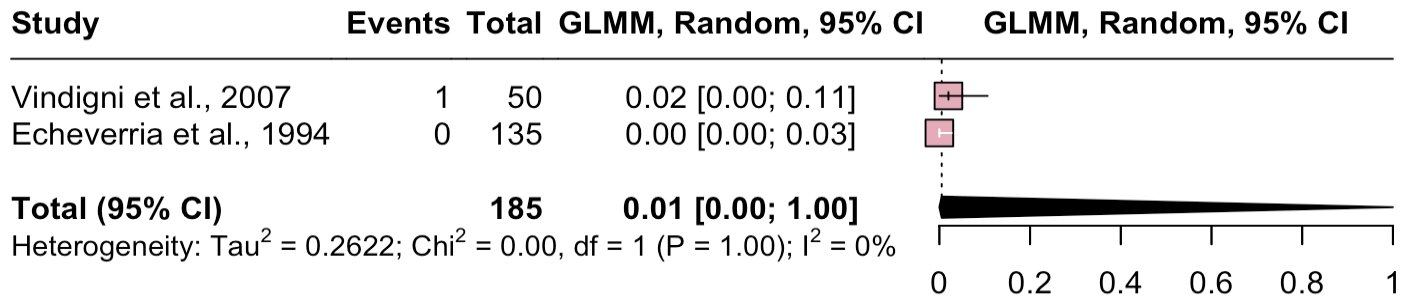
*

***All environment studies (n=6)***

*
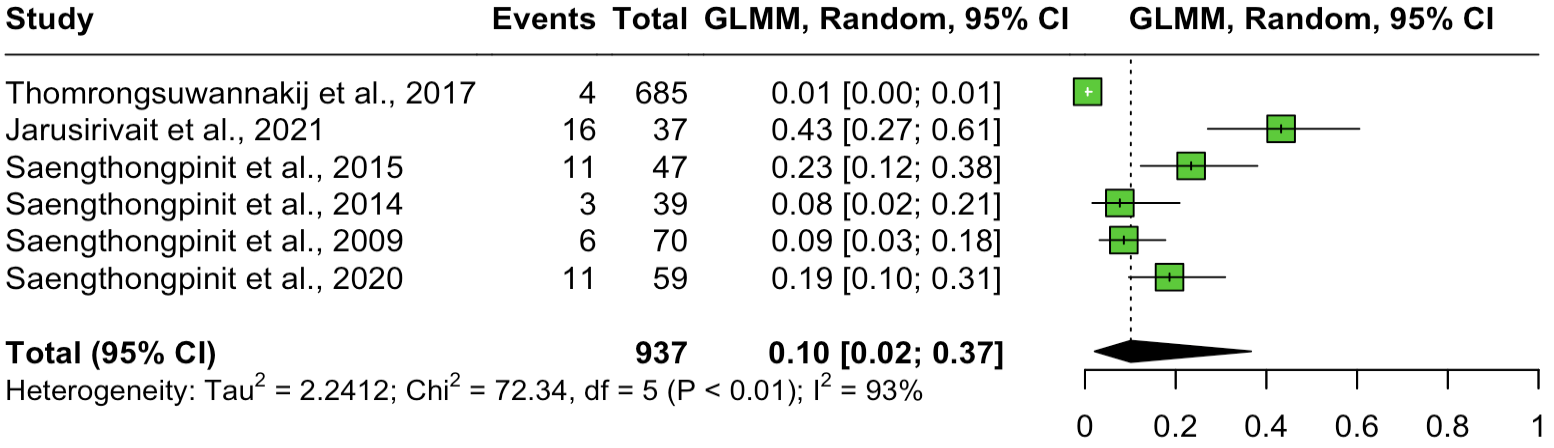
*

***Environment samples collected at chicken farm (n=3)***

*
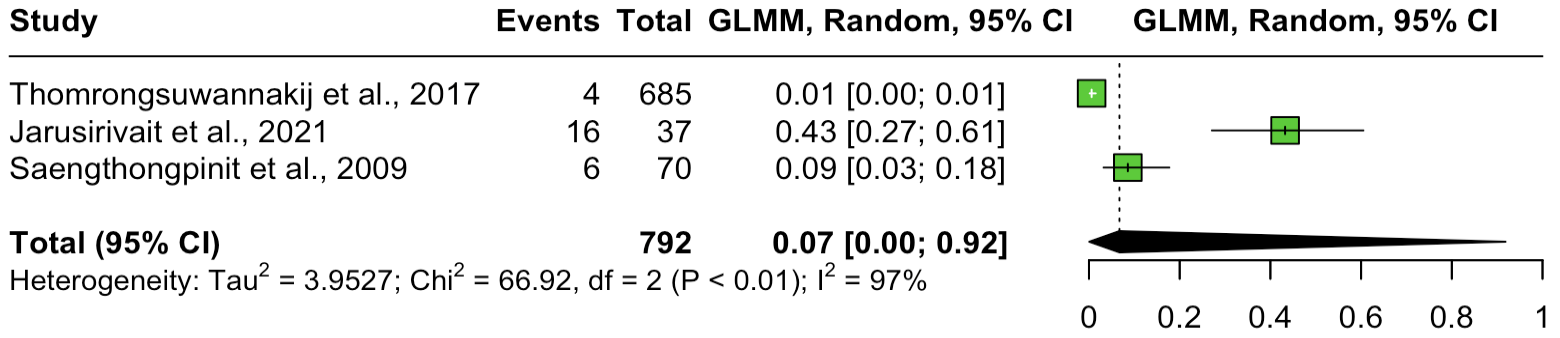
*

***Environment samples collected at duck farm (n=3)***

*
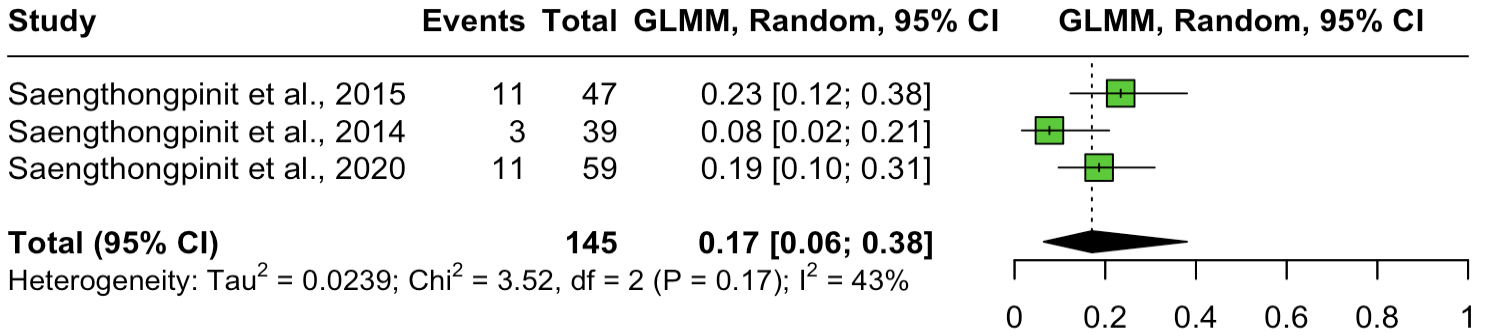
*

***A. Campylobacter coli***

***All human studies (n=11)***

*
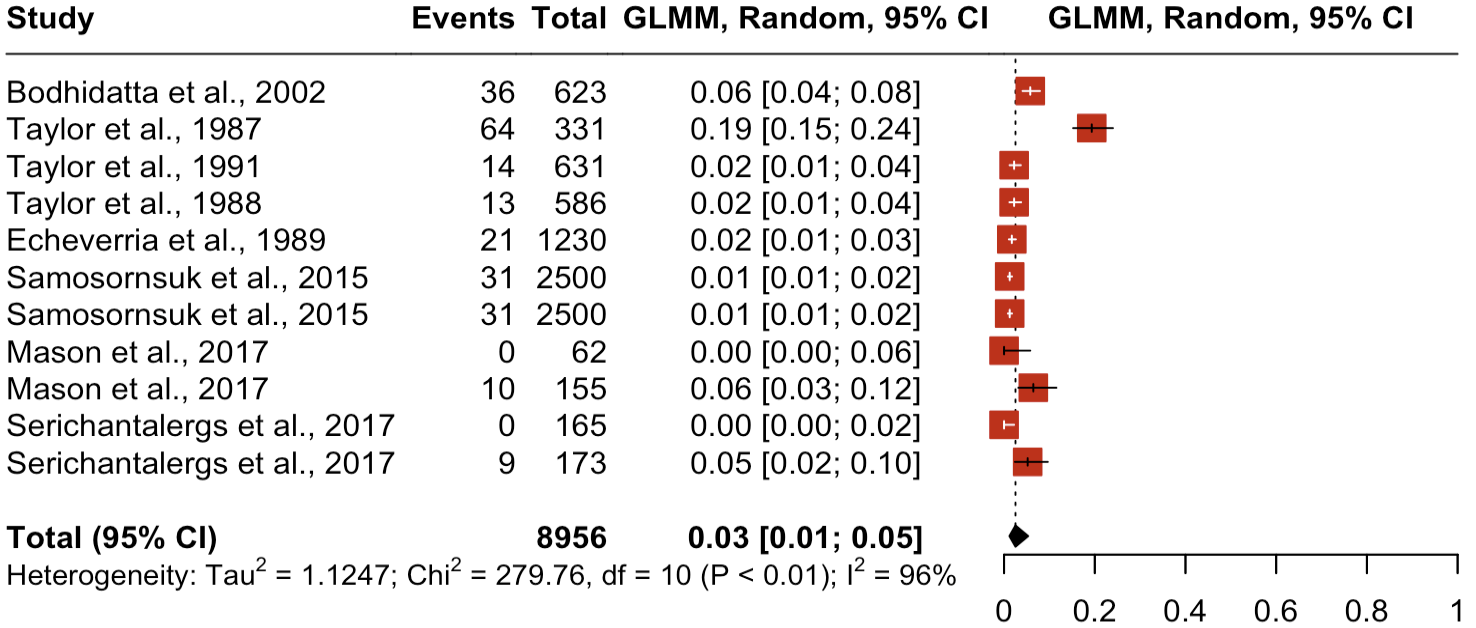
*

***Children (diarrhea) (n=6)***

*
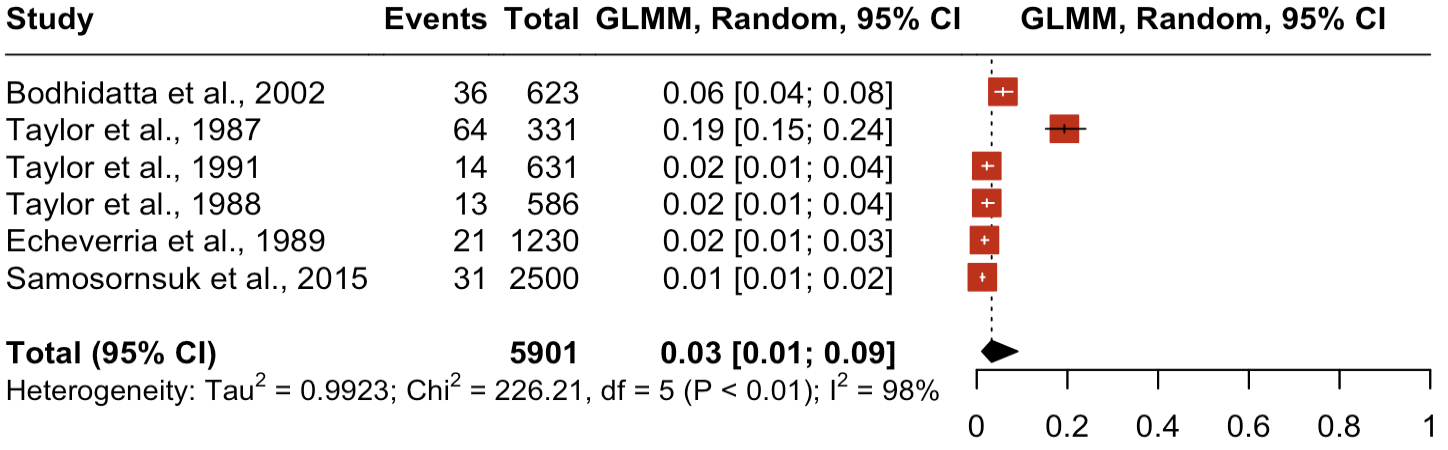
*

***General population (diarrhea) (n=3)***

*
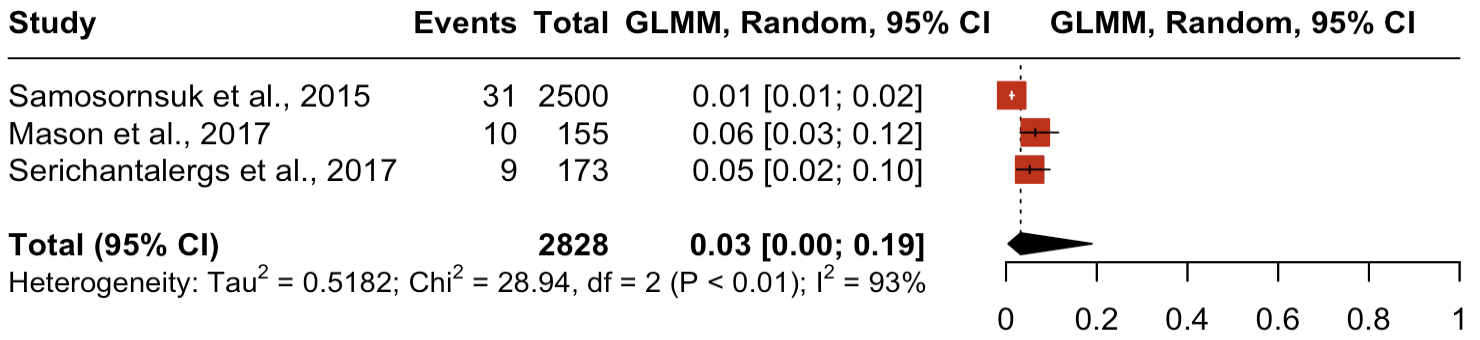
*

***General population (carriage) (n=2)***

*
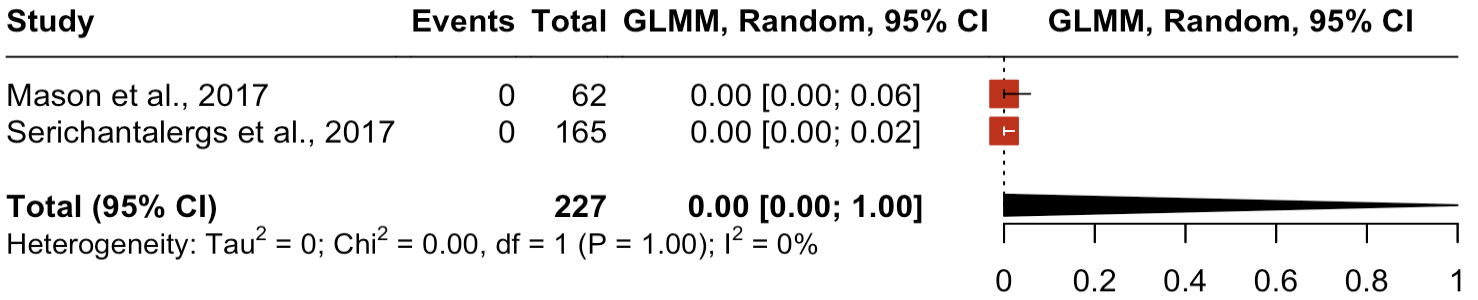
*

***All animal studies (n=11)***

***
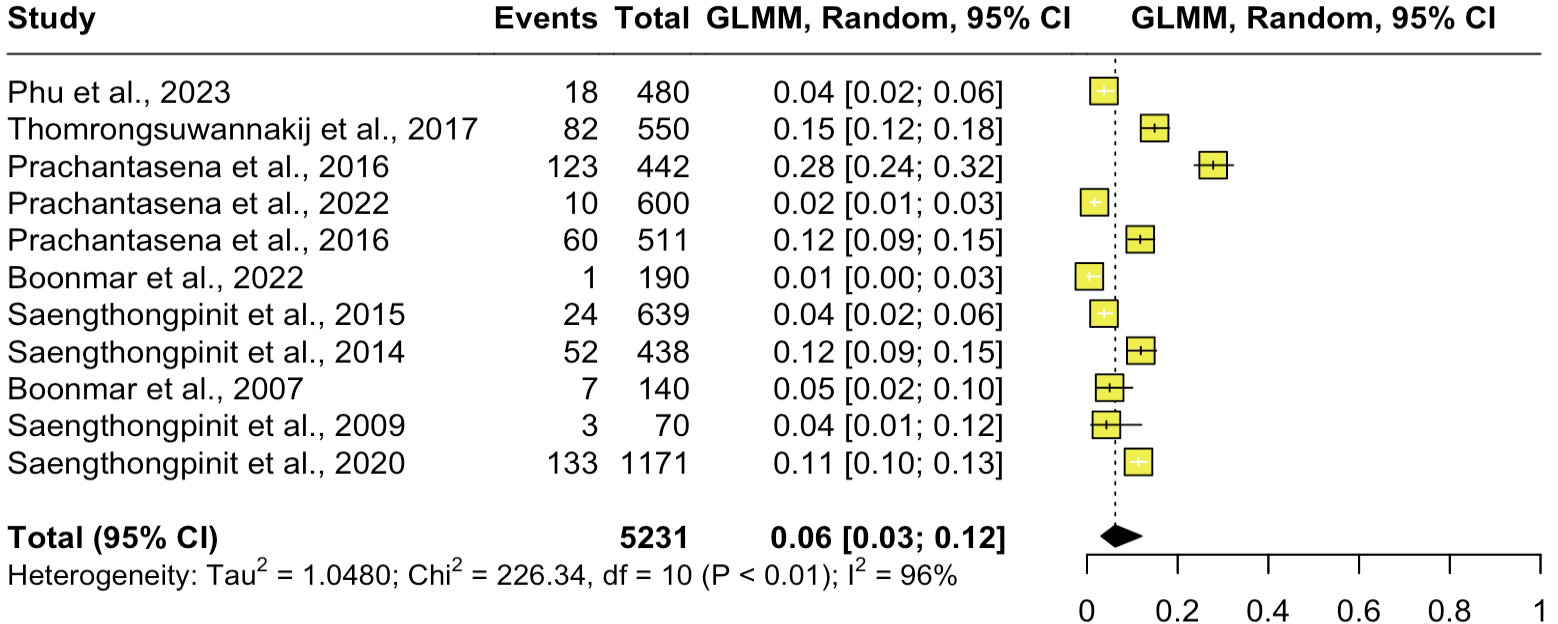
***

***Chicken (n=5)***

***
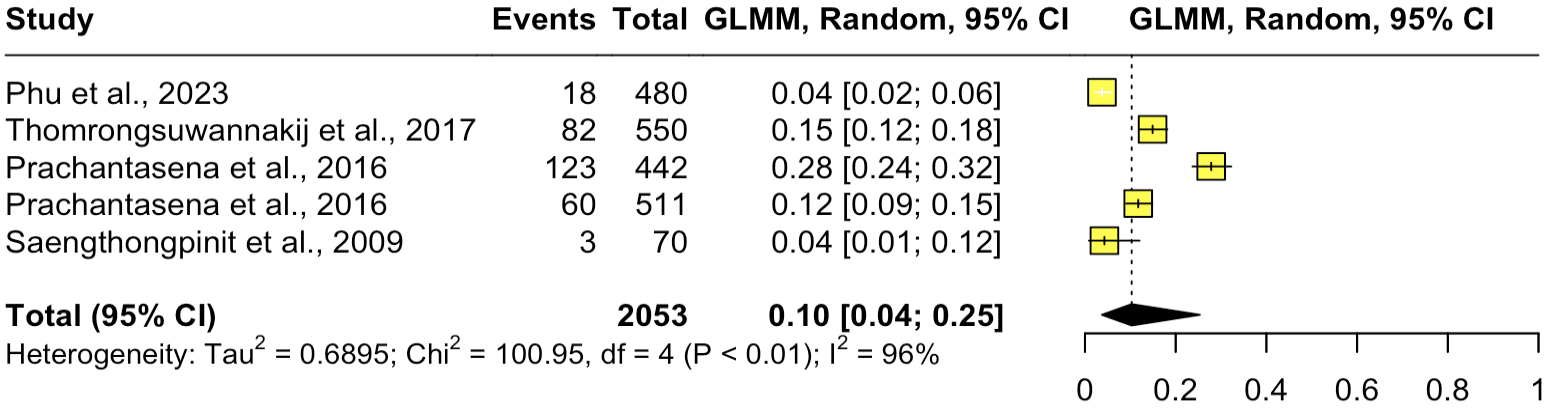
***

***Duck (n=4)***

***
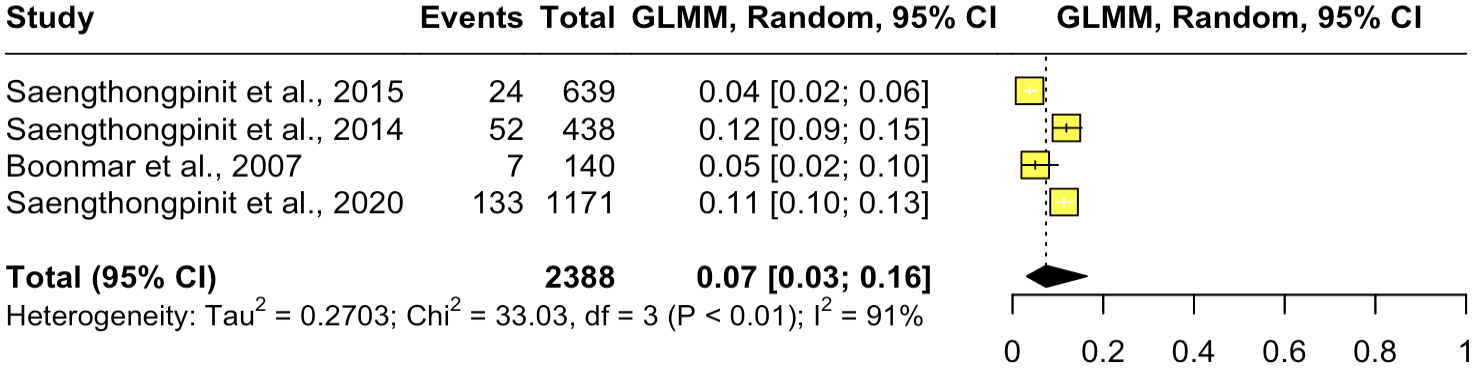
***

***Ruminant (n=2)***

***
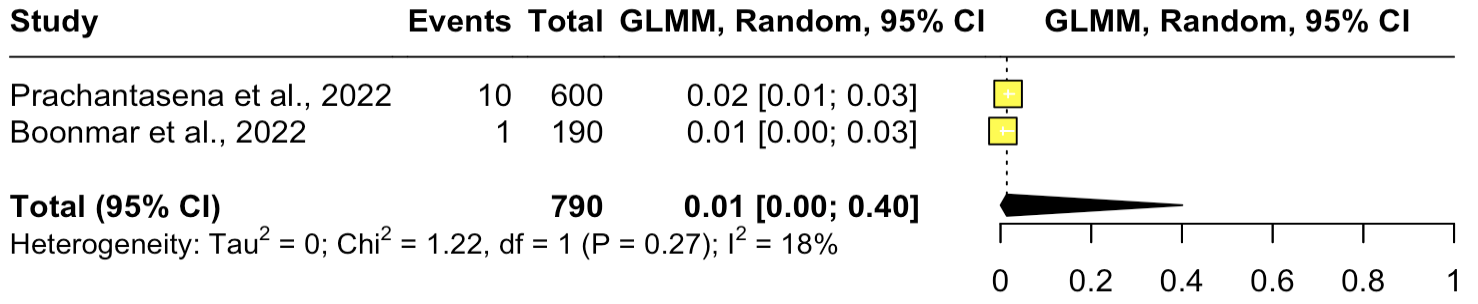
***

***All animal product studies (n=8) – Chicken products (n=8)***

***
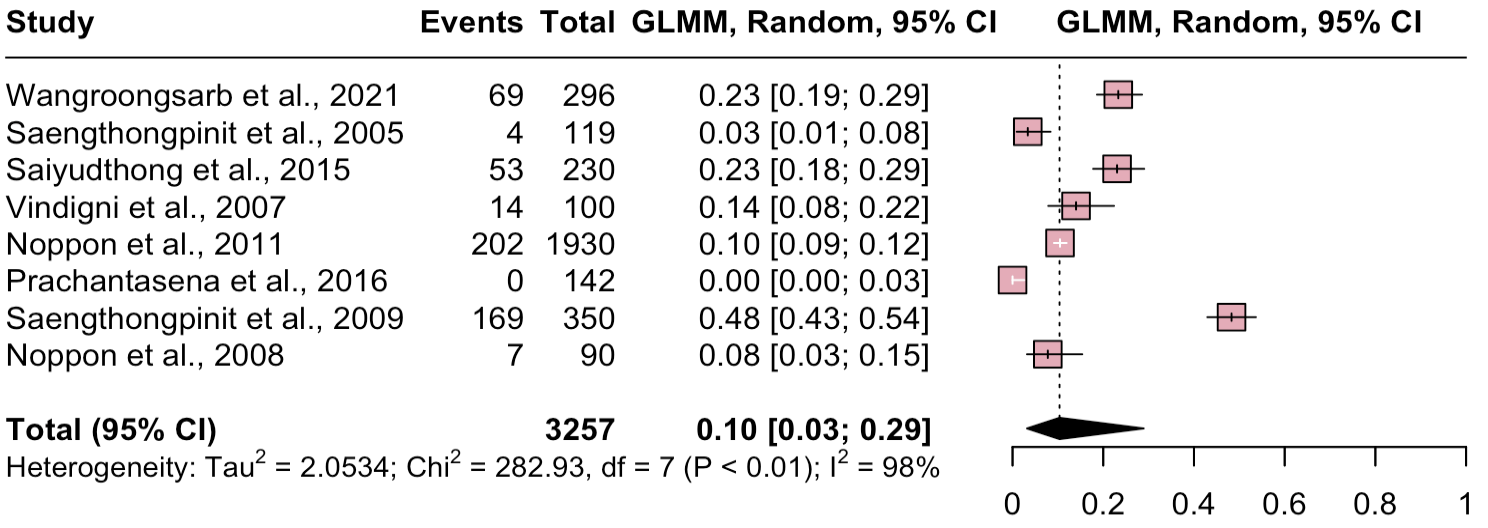
***

***All environment studies (n=5)***

***
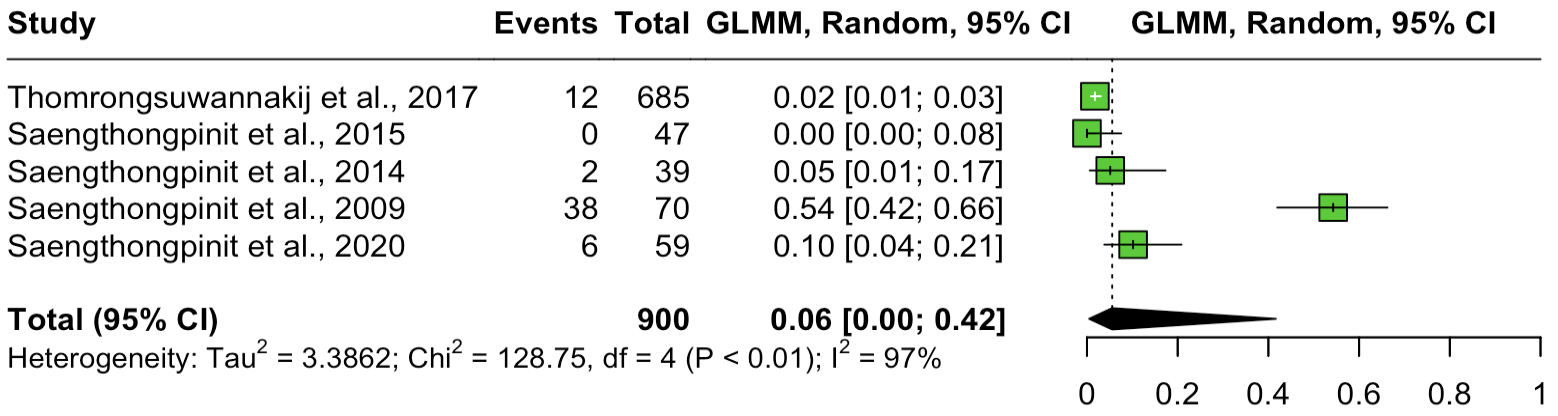
***

***Environment samples collected at chicken farm (n=2)***

***
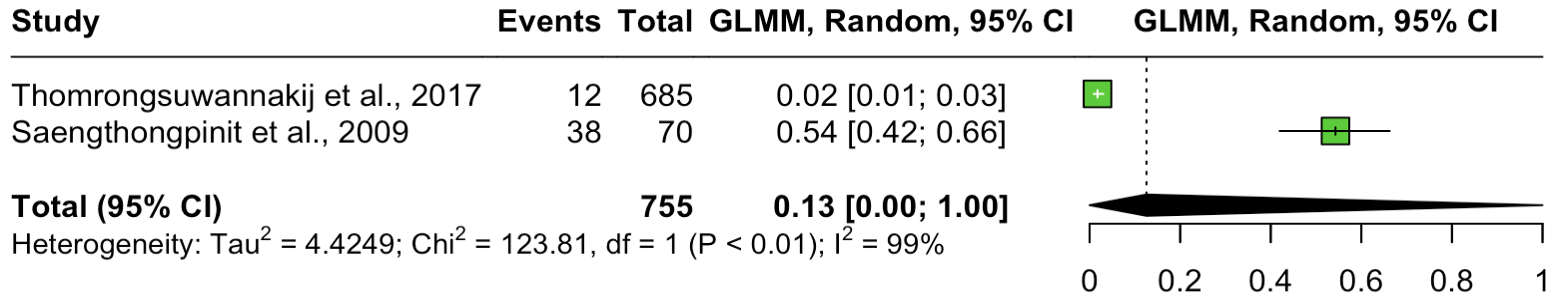
***

***Environment samples collected at duck farm (n=3)***

***
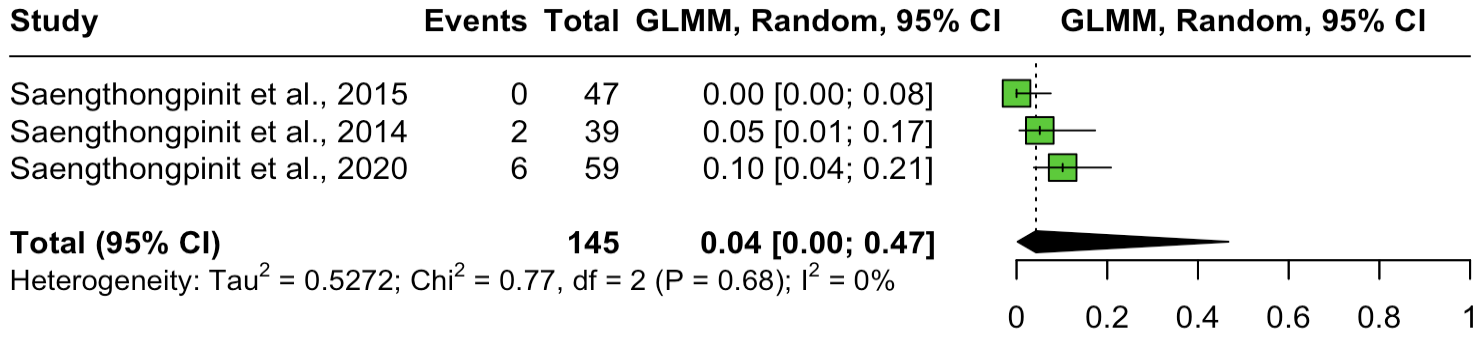
***
