# Supplementary material for: Prevalence and antimicrobial resistance of Campylobacter jejuni and Campylobacter coli over time in Thailand under a One Health approach: A systematic review and meta-analysis
Source: One Health. 2025 Jan 10;20:100965. doi: 10.1016/j.onehlt.2025.100965 (PMC11782884; doi:10.1016/j.onehlt.2025.100965)
Supplement: Supplementary Fig. 2 — Forest plots of AMR prevalence of C. jejuni and C. coli by different sources of sample collection. [file mmc2.docx]

**Supplementary Figure 2**: Forest plots of AMR prevalence of *C. jejuni* and *C. coli.* by different sources of sample collection.

***A. Ampicillin***

***C. jejuni – General population (diarrhea) (n=2)***

*
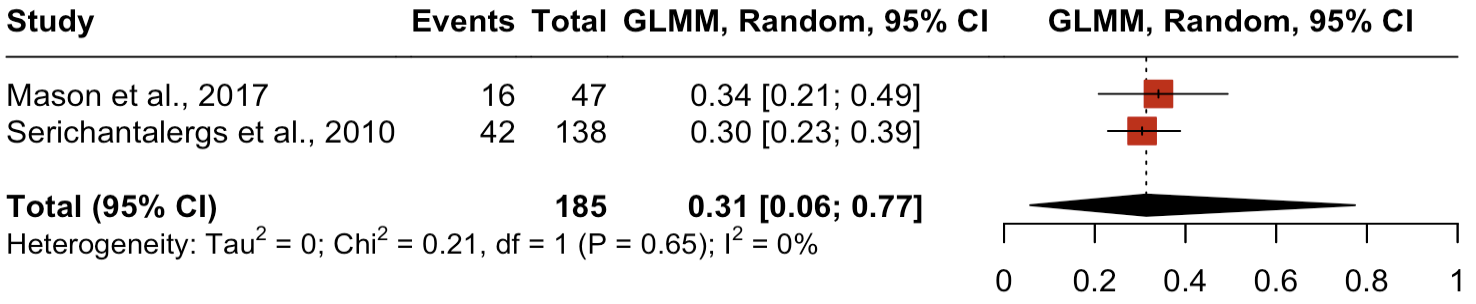
*

***C. jejuni – Chicken (n=4)***

*
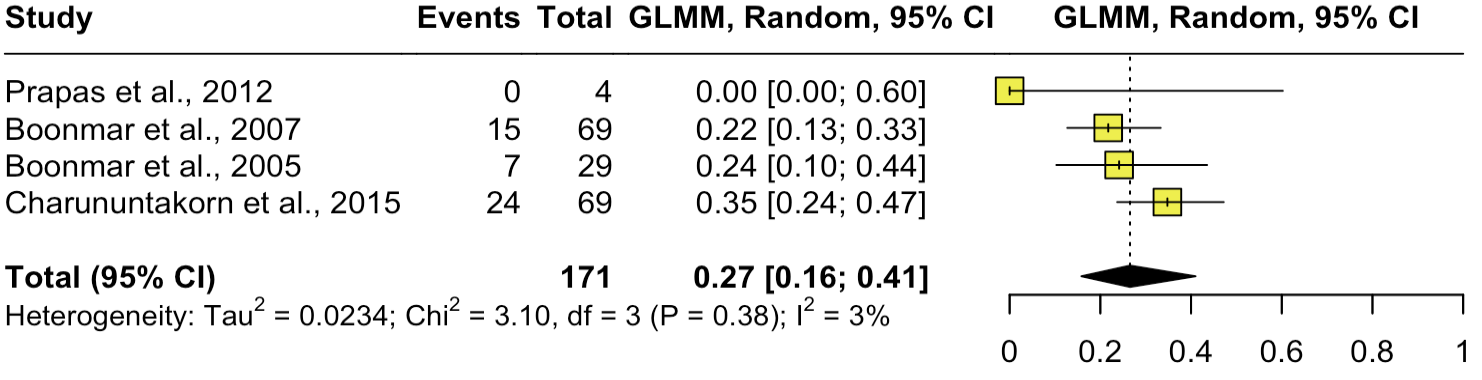
*

***C. jejuni – Chicken products (n=2)***

*
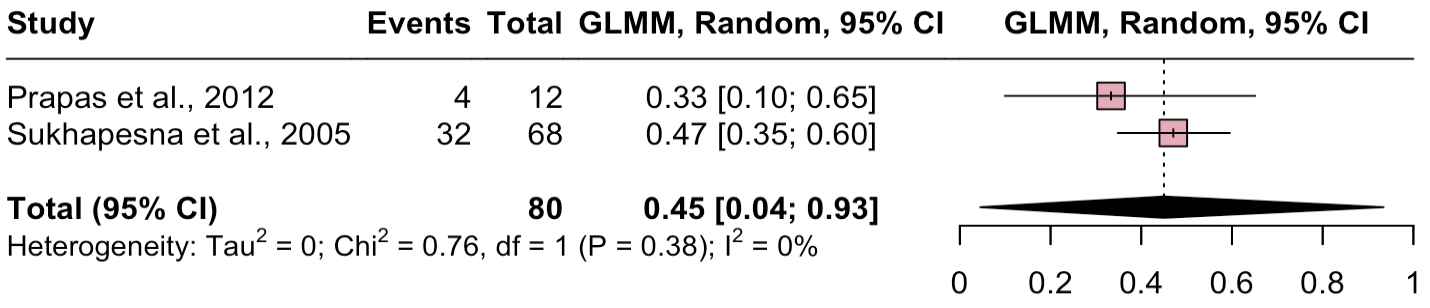
*

***B. Azithromycin***

***C. jejuni – Children (diarrhea) (n=3)***

***
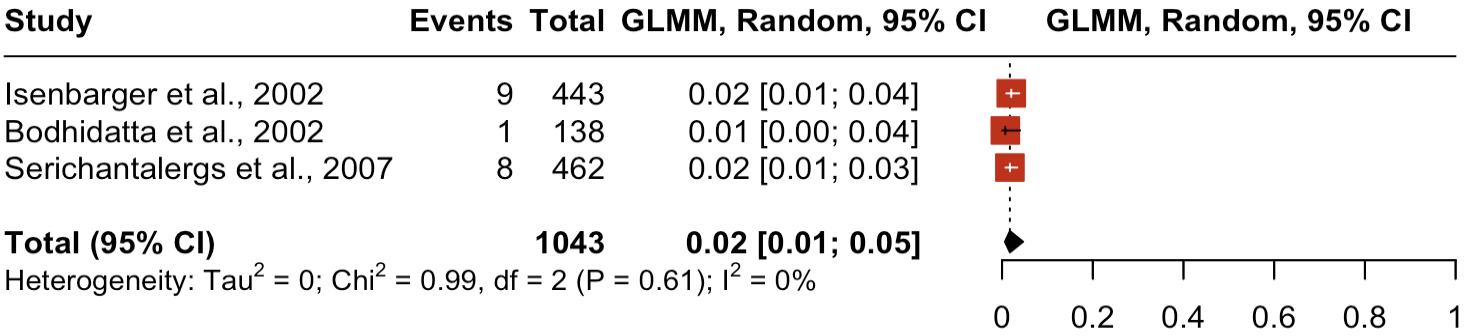
***

***C. jejuni – General population (diarrhea) (n=3)***

***
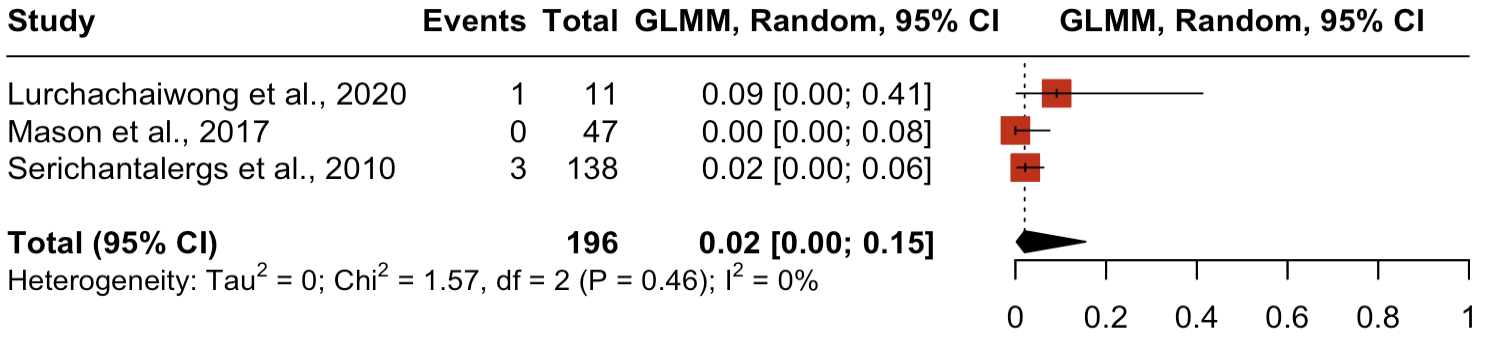
***

***C. coli – Children (diarrhea) (n=3)***

***
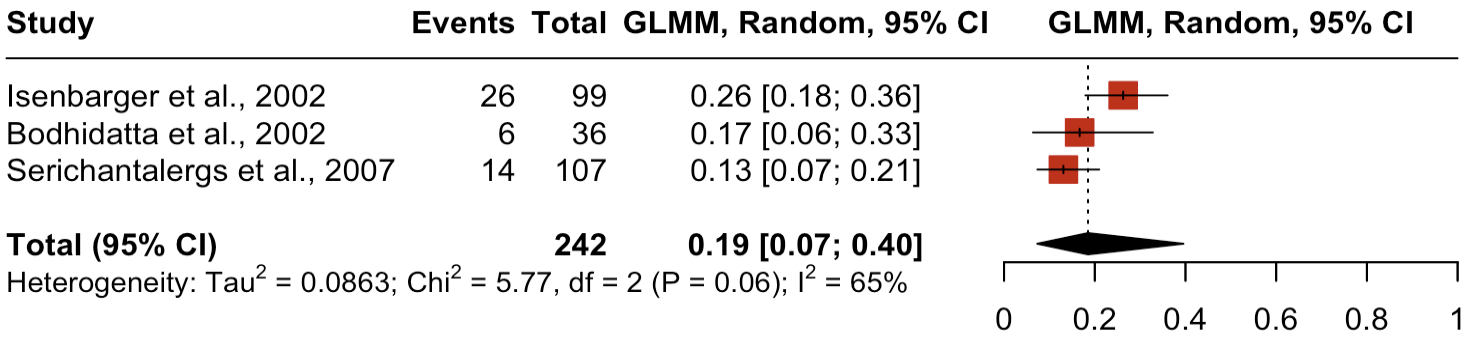
***

***C. Ciprofloxacin***

***C. jejuni – Children (diarrhea) (n=4)***

***
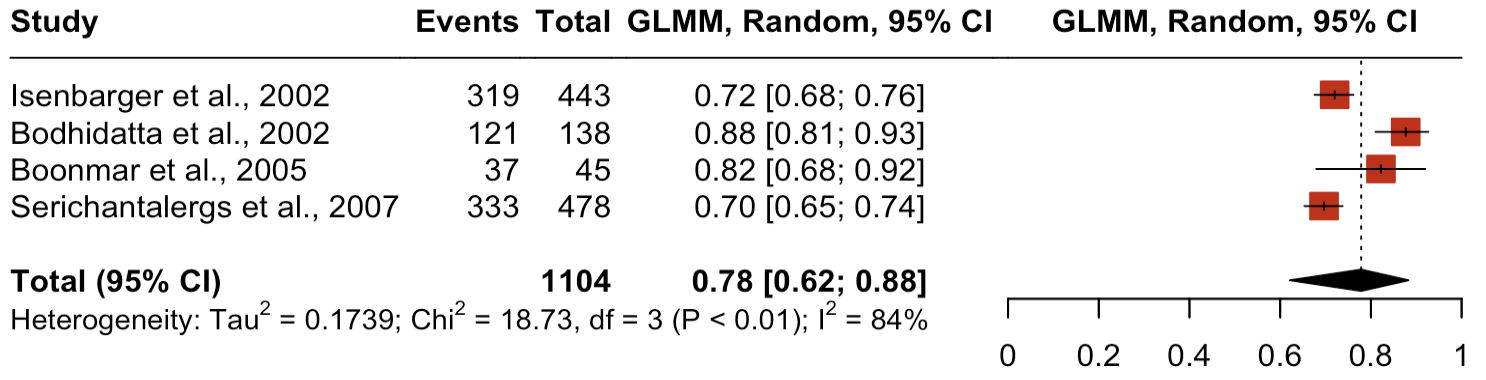
***

***C. jejuni – General population (diarrhea) (n=3)***

***
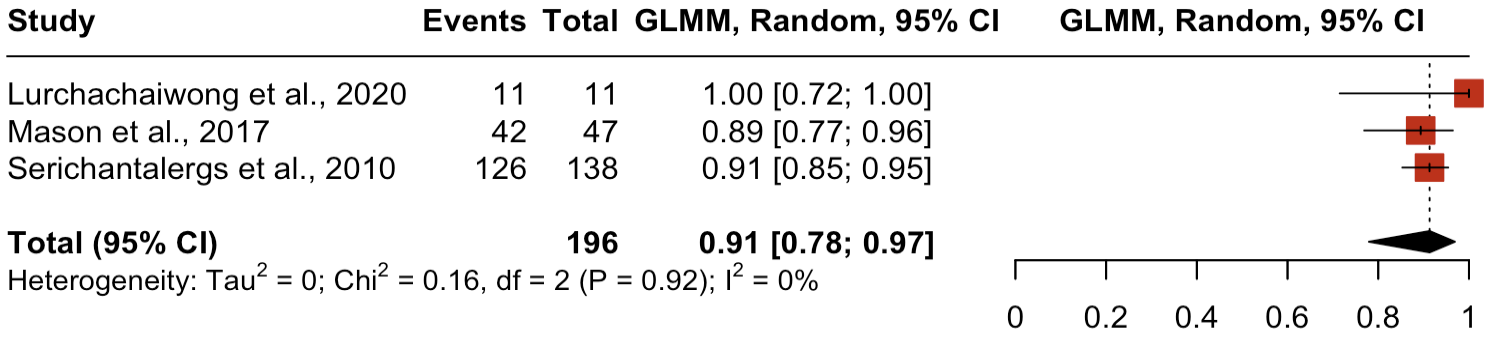
***

***C. jejuni – Chicken (n=6)***

***
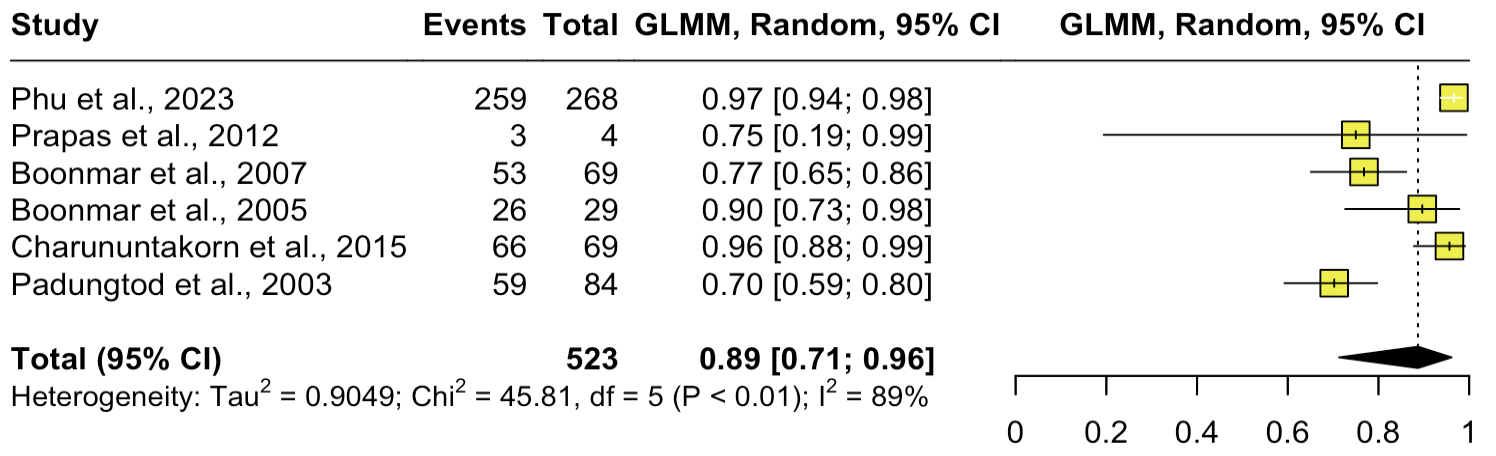
***

***C. jejuni – Chicken products (n=3)***

***
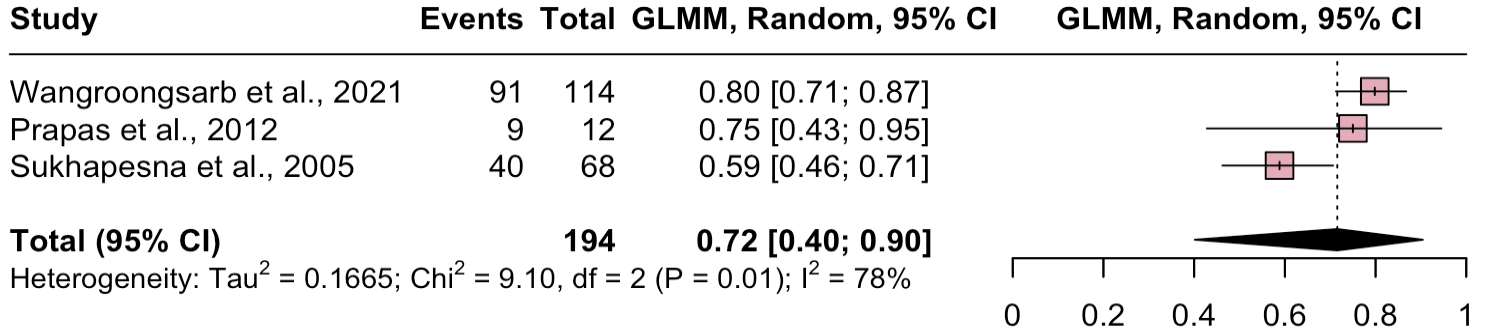
***

***C. coli – Children (diarrhea) (n=3)***

***
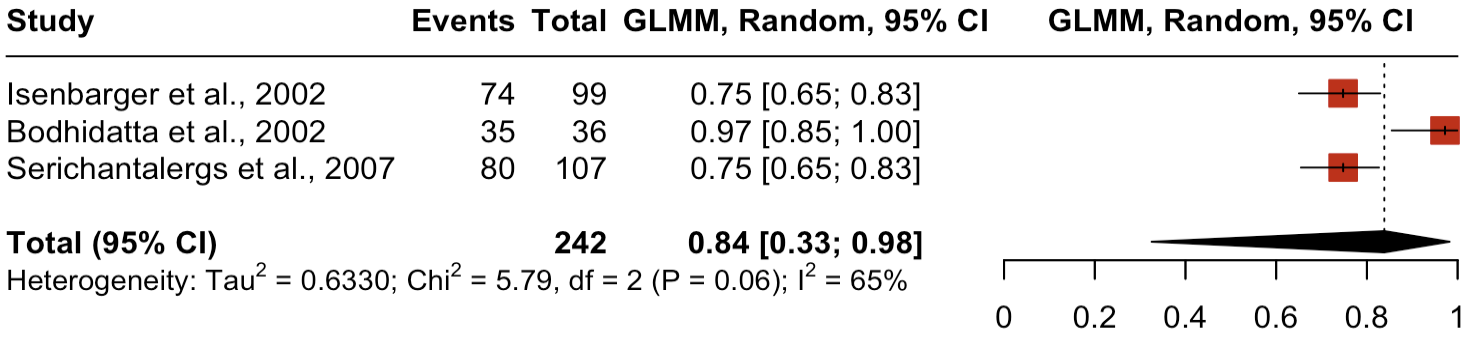
***

***D. Erythromycin***

***C. jejuni – Children (diarrhea) (n=4)***

***
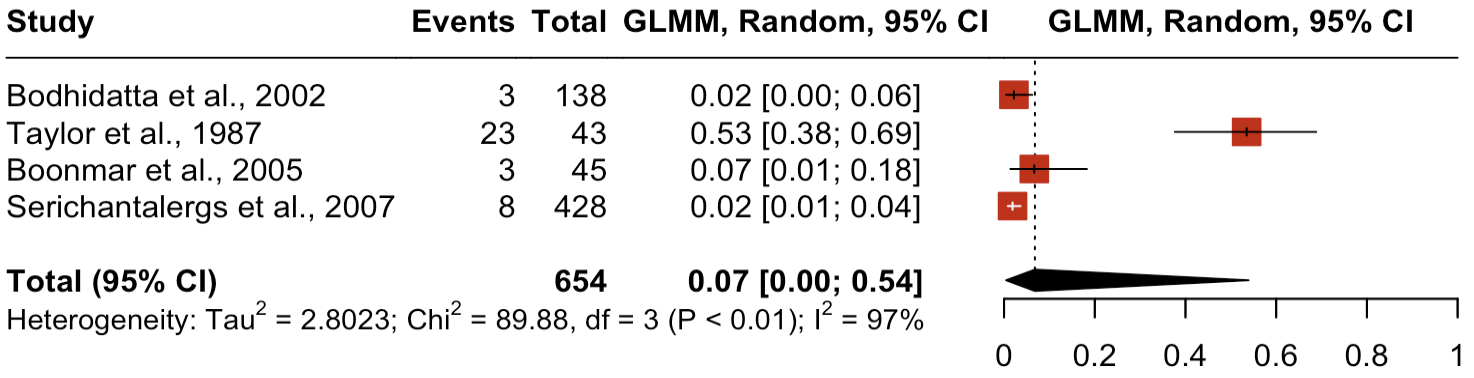
***

***C. jejuni – General population (diarrhea) (n=2)***

***
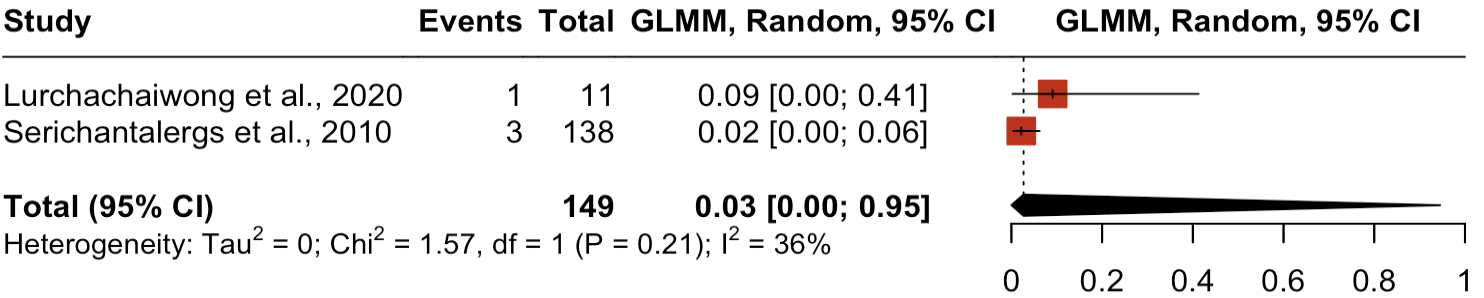
***

***C. jejuni – Chicken (n=5)***

***
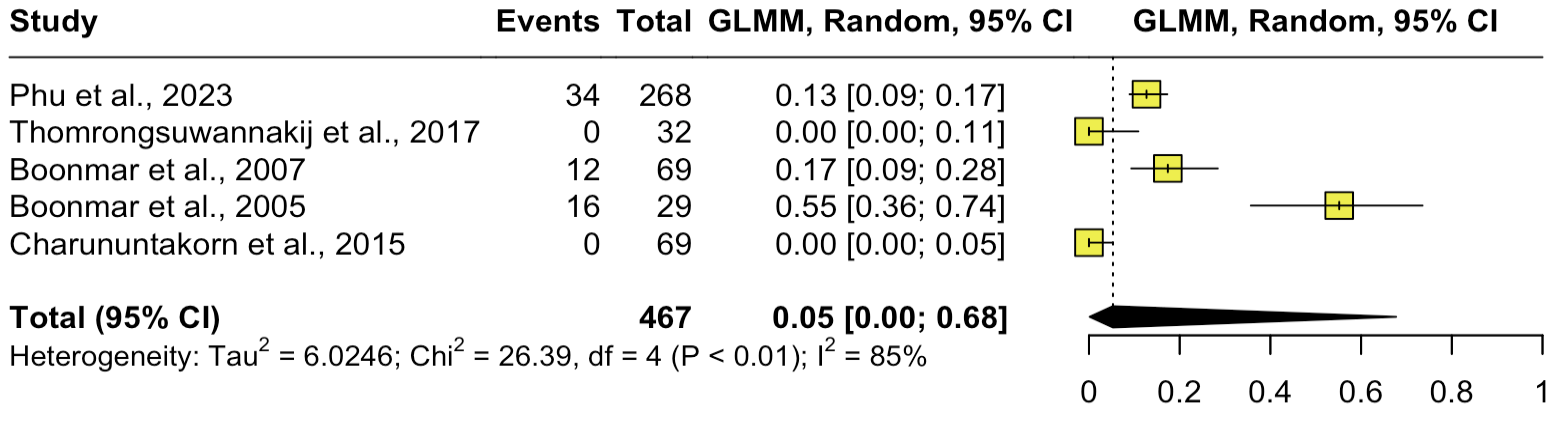
***

***C. jejuni – Chicken products (n=3)***

***
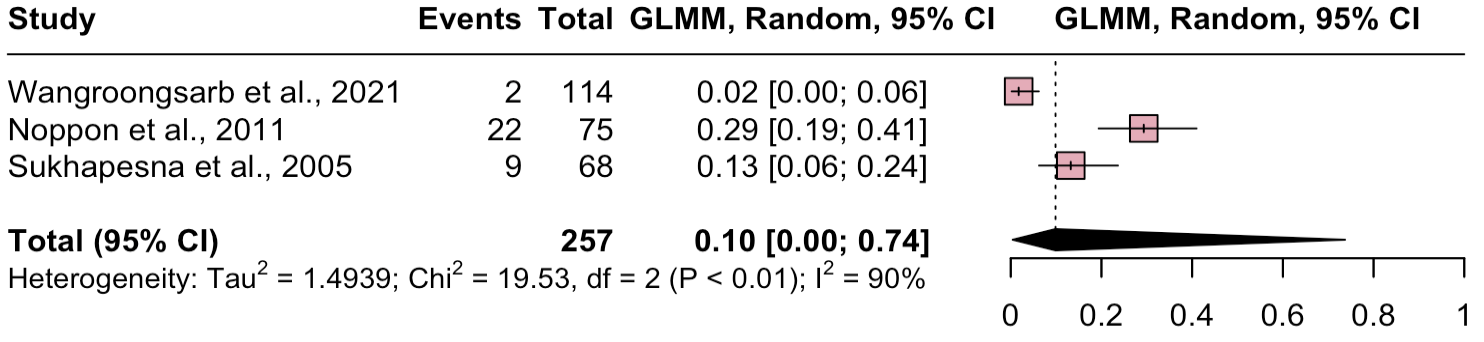
***

***C. coli – Children (diarrhea) (n=3)***

***
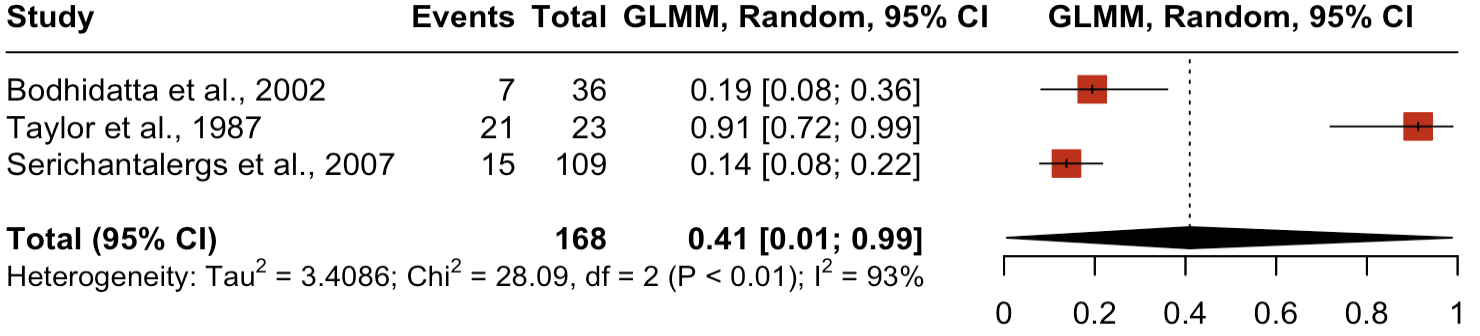
***

***C. coli – Chicken (n=2)***

***
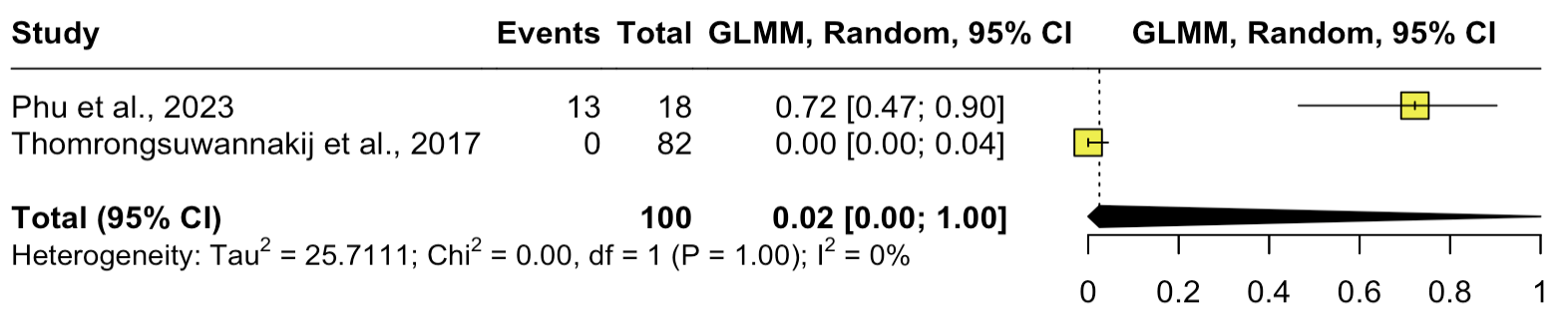
***

***E. Gentamicin***

***C. jejuni – Chicken (n=2)***

***
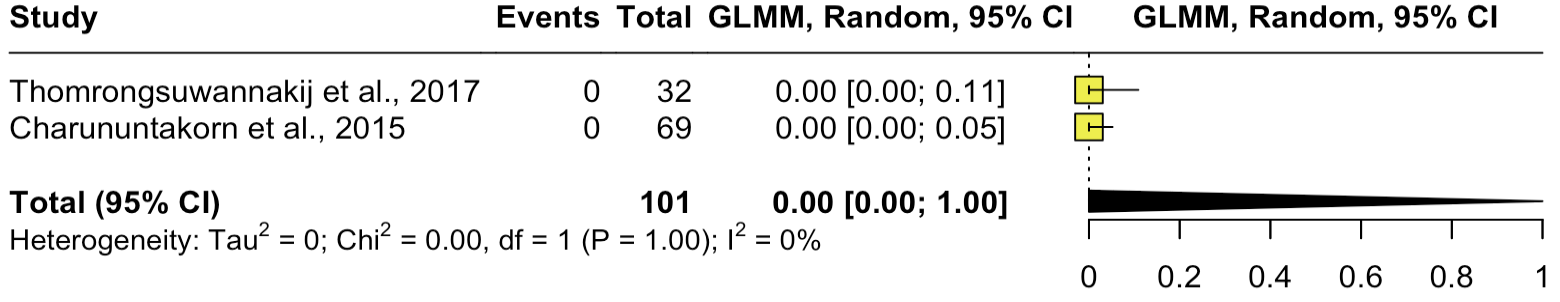
***

***C. jejuni – Chicken product (n=2)***

***
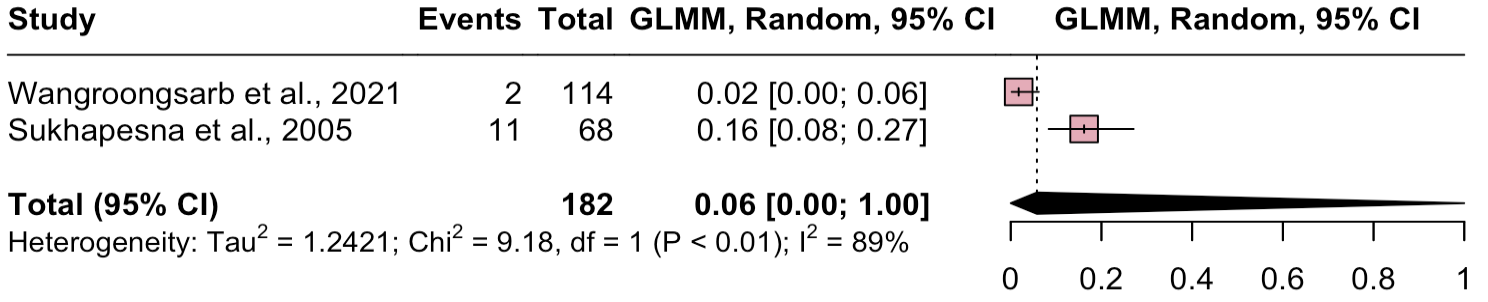
***

***F. Nalidixic acid***

***C. jejuni – Children (diarrhea) (n=4)***

***
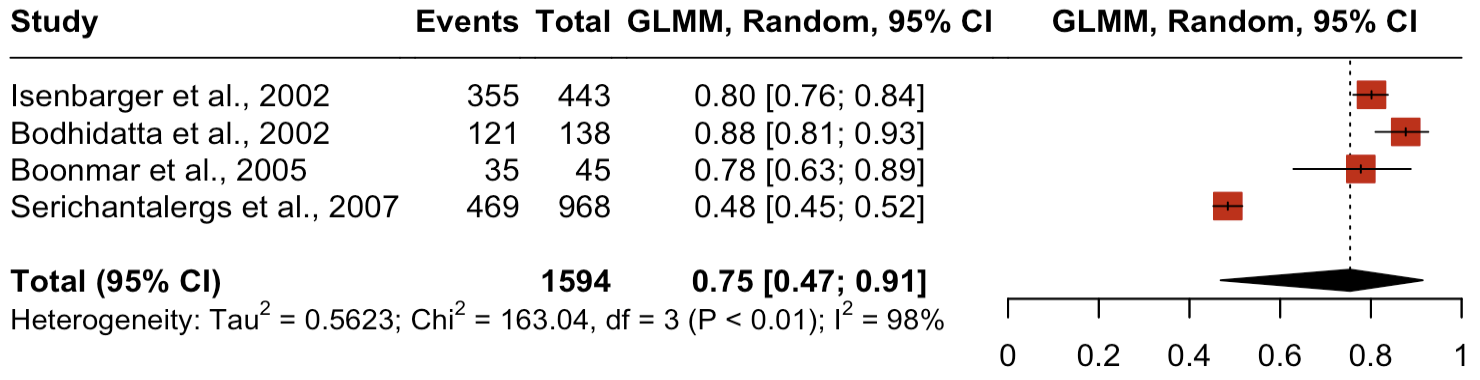
***

***C. jejuni – General population (diarrhea) (n=3)***

***
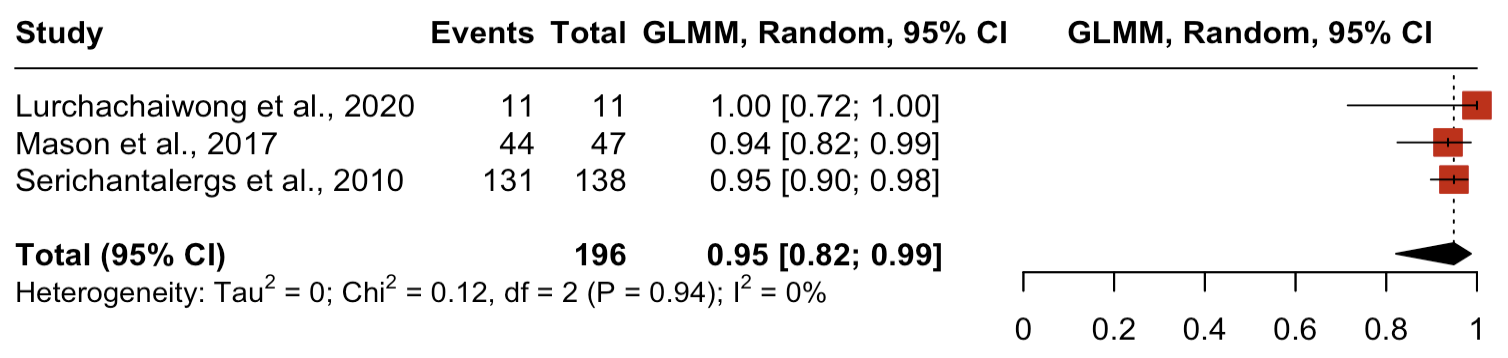
***

***C. jejuni – Chicken (n=4)***

***
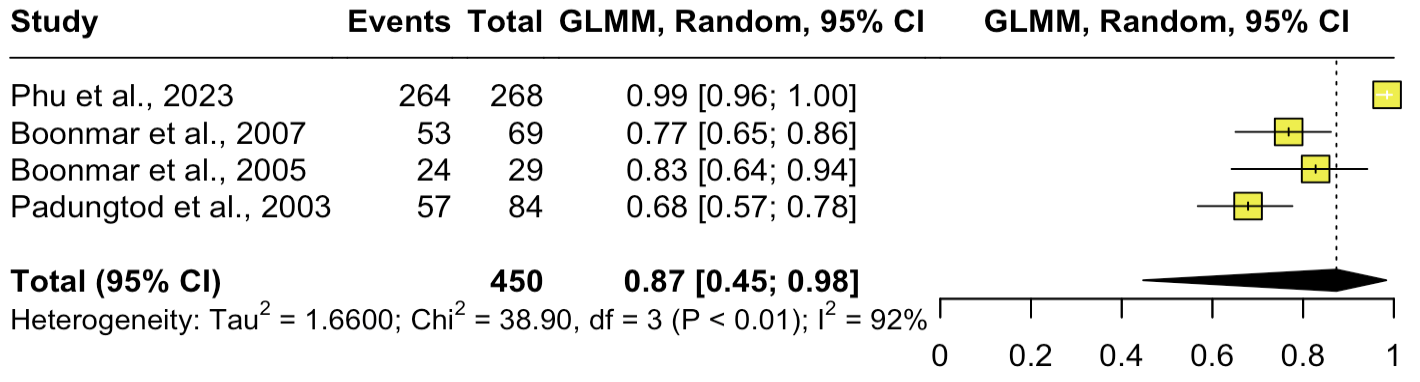
***

***C. jejuni – Chicken products (n=2)***

***
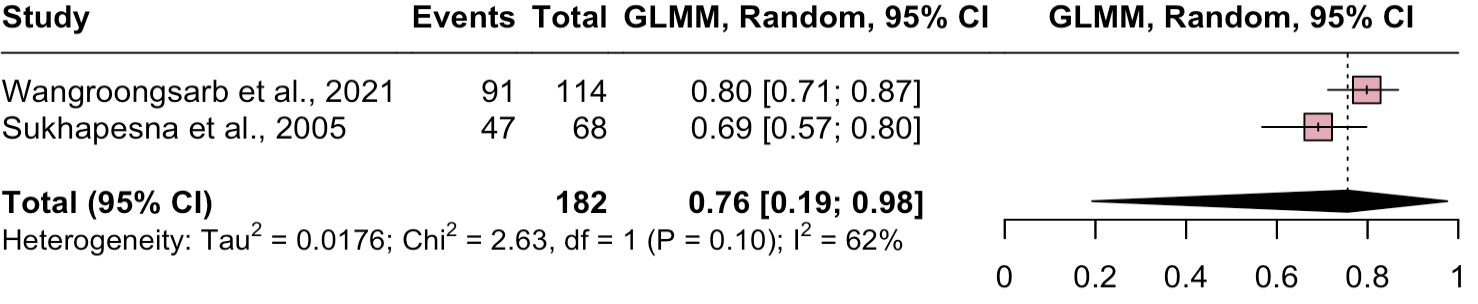
***

***C. coli – Children (diarrhea) (n=3)***

***
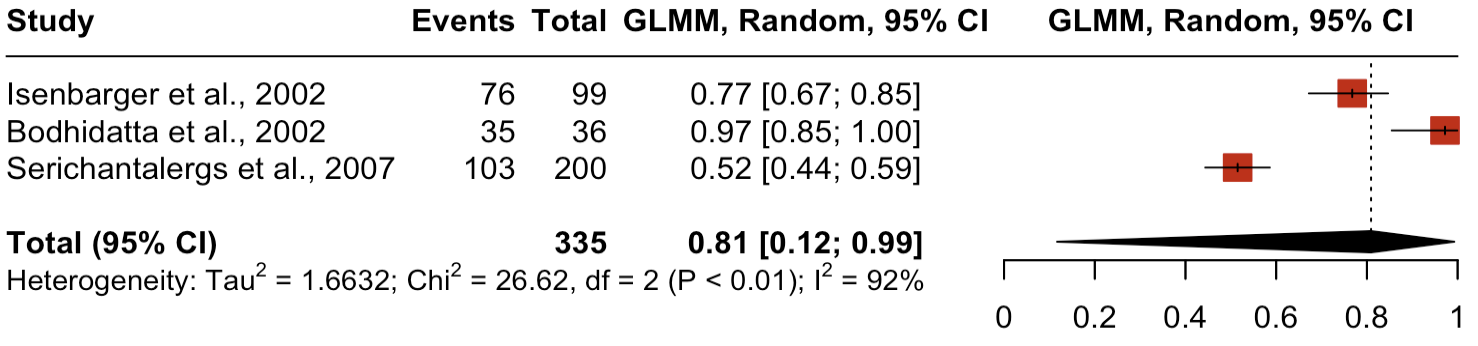
***

***G. Trimethoprim-Sulfamethoxazole***

***C. jejuni – General population (diarrhea) (n=2)***

***
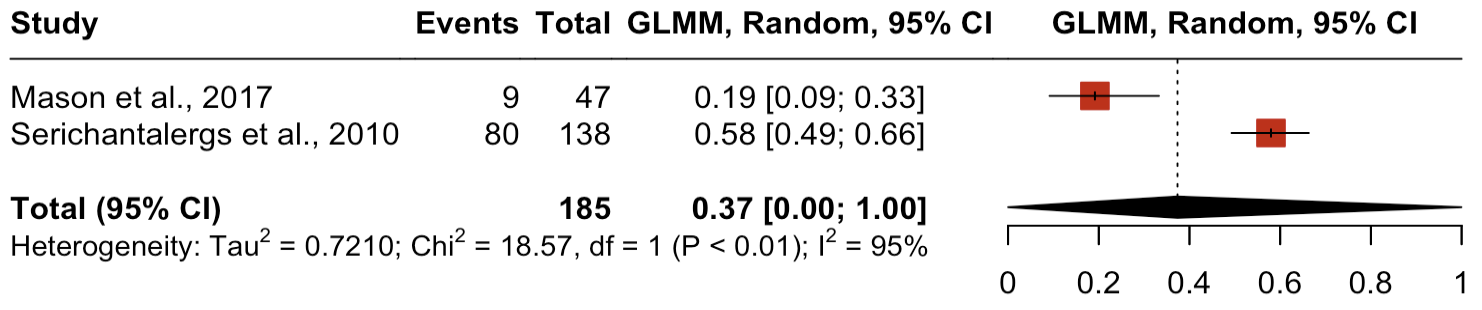
***

***C. jejuni – Chicken (diarrhea) (n=2)***

***
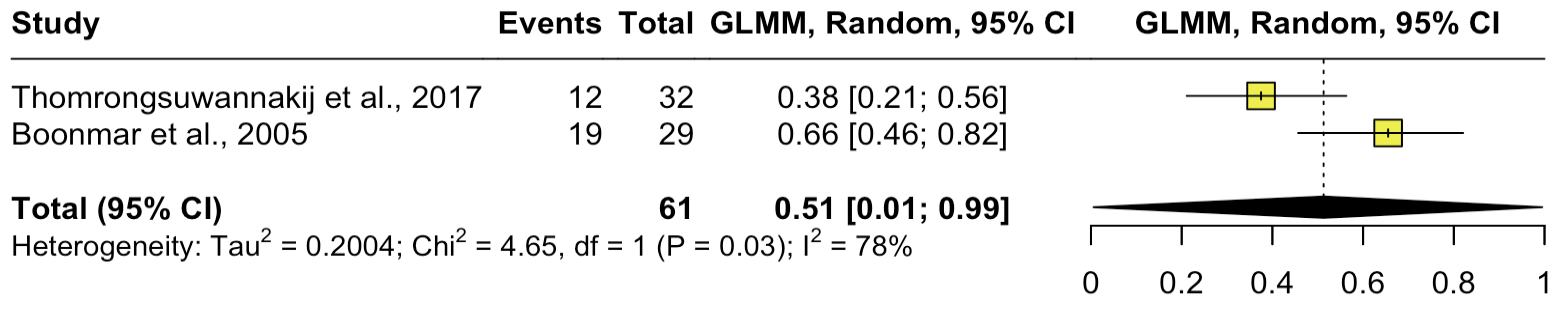
***

***H. Tetraycycline***

***C. jejuni – Children (diarrhea) (n=2)***

***
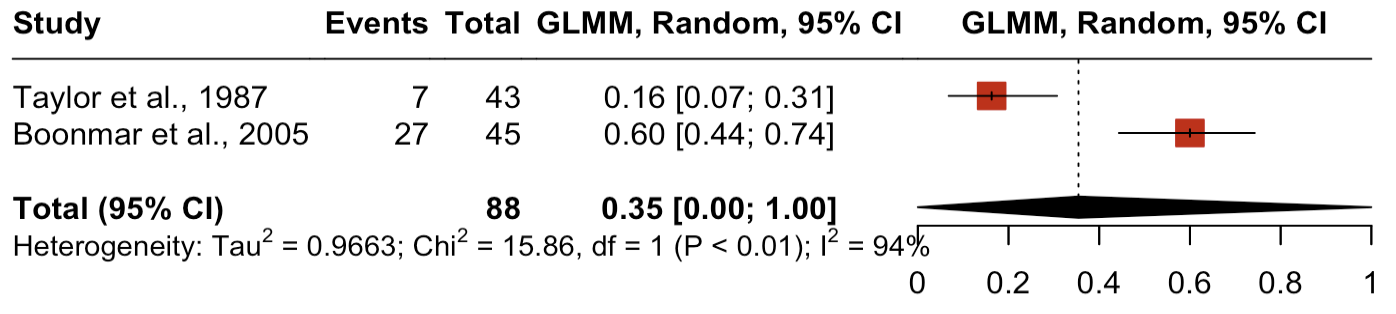
***

***C. jejuni – General population (diarrhea) (n=2)***

***
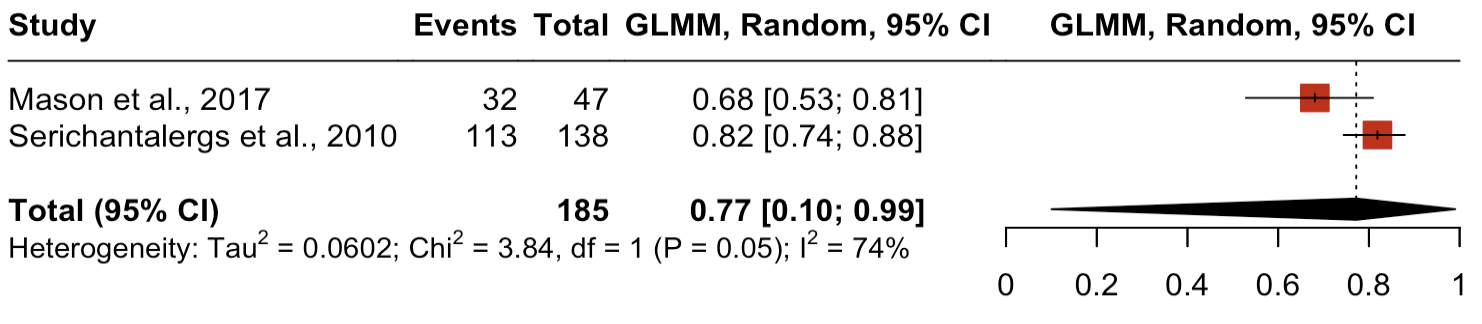
***

***C. jejuni – Chicken (n=6)***

***
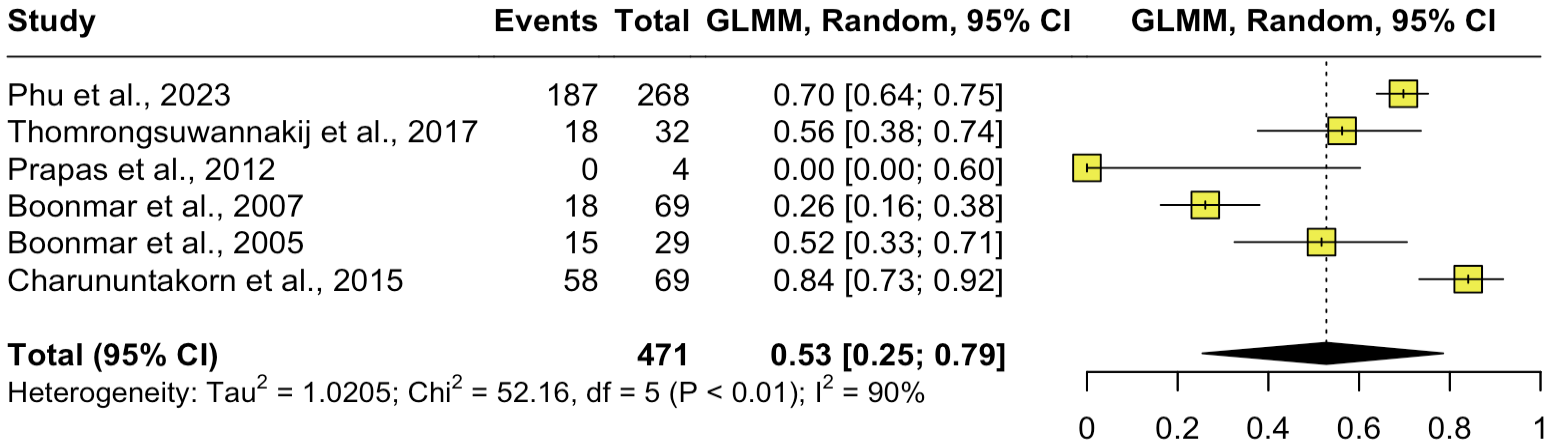
***

***C. jejuni – Chicken products (n=3)***

***
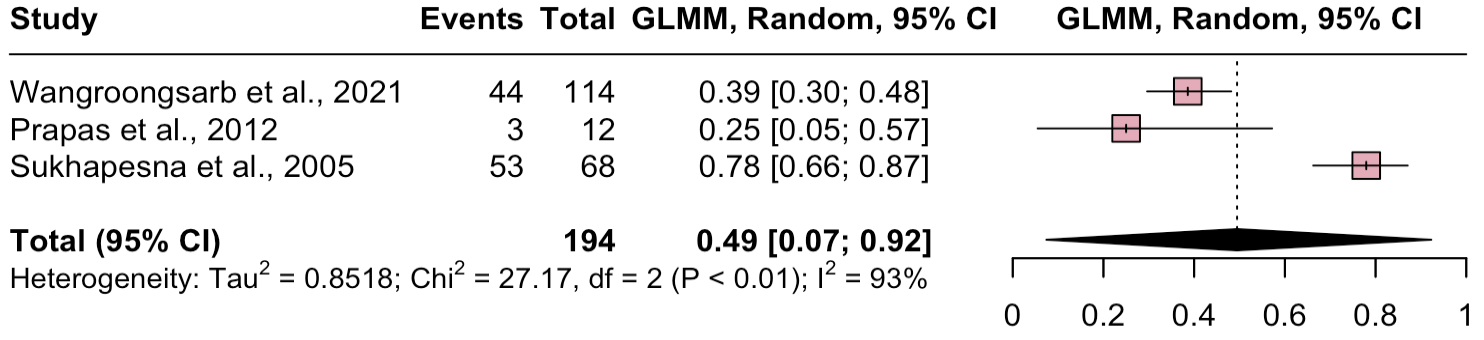
***

***C. coli – Chicken (n=2)***

***
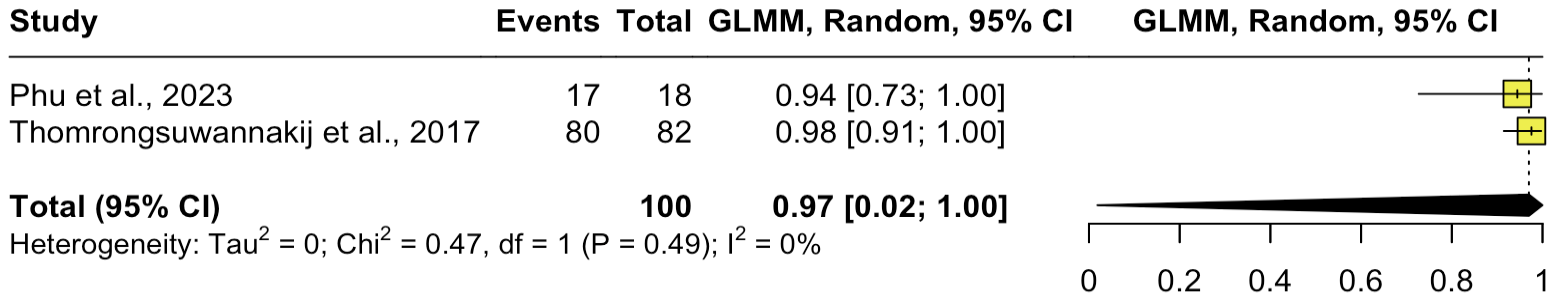
***
