# Supplementary material for: Prevalence and antimicrobial resistance of Campylobacter jejuni and Campylobacter coli over time in Thailand under a One Health approach: A systematic review and meta-analysis
Source: One Health. 2025 Jan 10;20:100965. doi: 10.1016/j.onehlt.2025.100965 (PMC11782884; doi:10.1016/j.onehlt.2025.100965)
Supplement: Supplementary Fig. 3 — Contour-enhanced funnel plots and Egger's tests used for publication bias assessment. [file mmc3.docx]

**Supplementary Figure 3**: Contour-enhanced funnel plots and Egger’s tests used for publication bias assessment.

**A.** The asymmetry among selected studies exhibits on the contour-enhanced funnel plots used for publication bias assessment using funnel plots of *Campylobacter* prevalence. Each scatter illustrates each selected article. The dashed vertical line shows the average effect sizes. The shaded regions illustrate the statistical significance of asymmetry of the publication (*p* < 0.1 (in gray), *p* < 0.05 (in dark yellow), *p* < 0.01(in yellow)).

| **Categories** | **No. prevalence estimate** | **Contour-enhanced funnel plots** | **Egger’s test** | |
| --- | --- | --- | --- | --- |
|  |  |  | ***βo*** | ***P-value*** |
| ***C. jejuni*** |  |  |  |  |
| **Humans** |  |  |  |  |
| Children (diarrhea) | 6 | 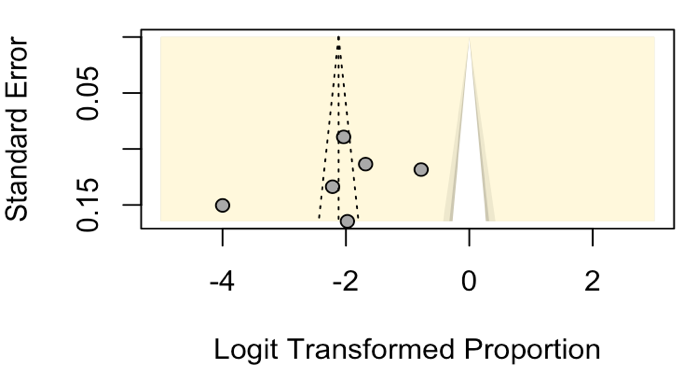 | -0.74 | 0.499 |
| General population (diarrhea) | 5 | 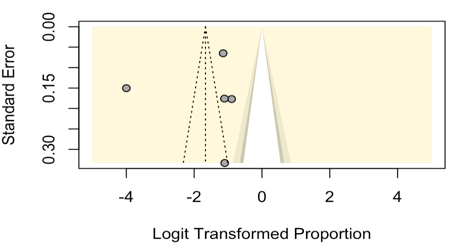 | -0.46 | 0.674 |
| General population (carriage) | 2 | 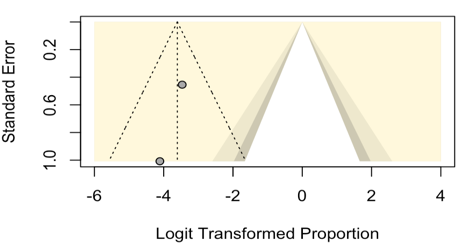 | nc | nc |
| **Animals** |  |  |  |  |
| Chicken | 8 | 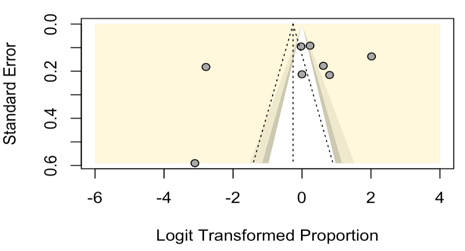 | -0.60 | 0.568 |
| Duck | 4 | 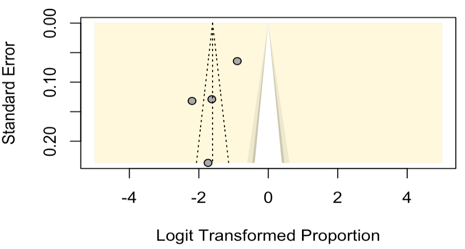 | -1.86 | 0.204 |
| Ruminant | 2 | 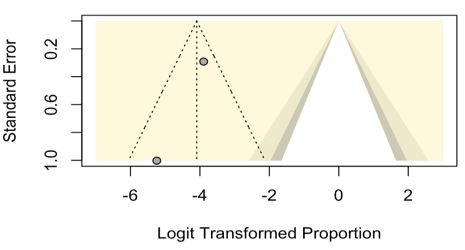 | nc | nc |
| **Animal products** |  |  |  |  |
| Chicken products | 11 | 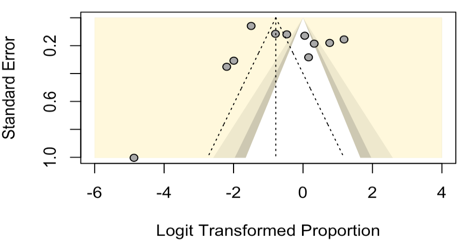 | 1.11 | 0.297 |
| Pork | 2 | 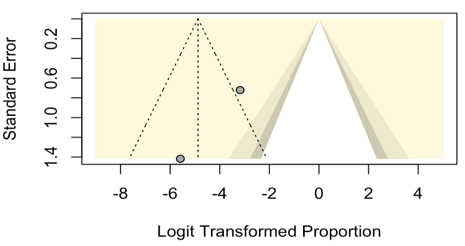 | nc | nc |
| Ruminant products | 2 | 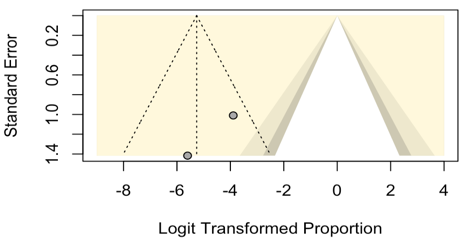 | nc | nc |
| **Environment** |  |  |  |  |
| Samples collected at chicken farm | 3 | 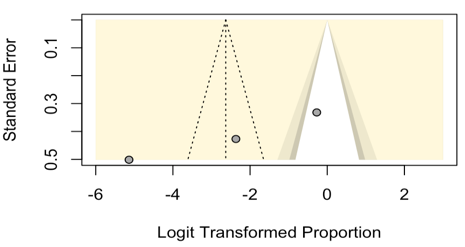 | -6.80 | 0.093 |
| Samples collected at duck farm | 3 | 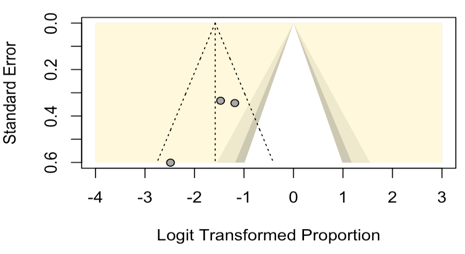 | -2.52 | 0.240 |
| ***C. coli*** |  |  |  |  |
| **Humans** |  |  |  |  |
| Children (diarrhea) | 6 | 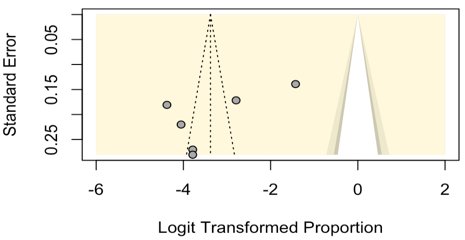 | -1.95 | 0.122 |
| General population (diarrhea) | 3 | 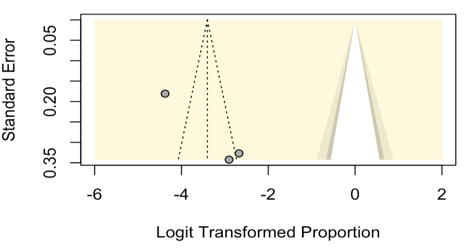 | -4.49 | 0.097 |
| General population (carriage) | 2 | 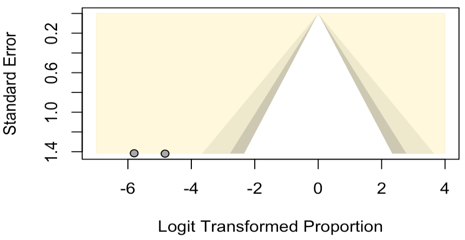 | nc | nc |
| **Animals** |  |  |  |  |
| Chicken | 5 | 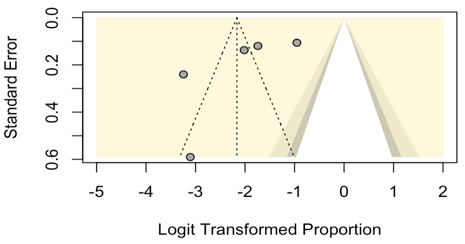 | -1.68 | 0.193 |
| Duck | 4 | 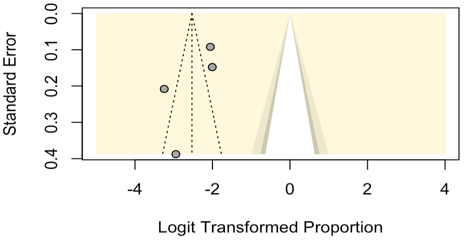 | -1.49 | 0.274 |
| Ruminant | 2 | 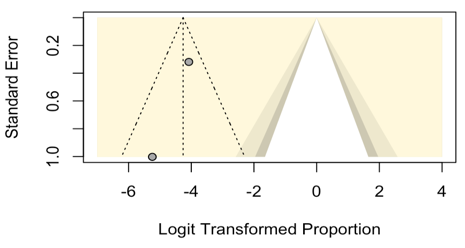 | nc | nc |
| **Animal products –**  Chicken products | 8 | 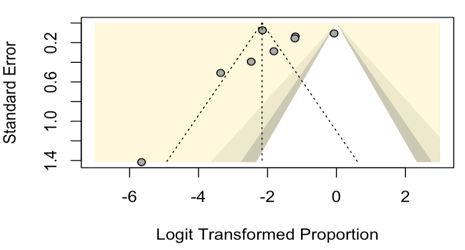 | -0.29 | 0.778 |
| **Environment** |  |  |  |  |
| Samples collected at chicken farm | 2 | 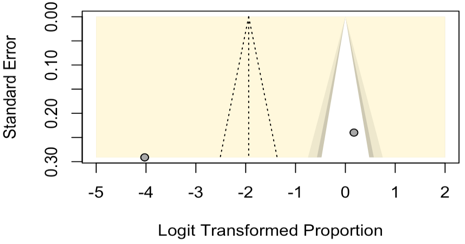 | nc | nc |
| Samples collected at duck farm | 3 | 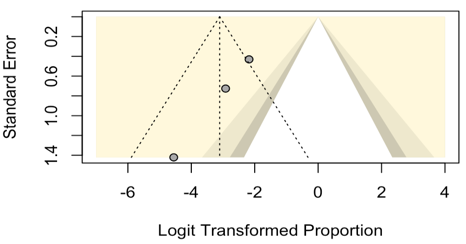 | -8.85 | 0.013 |

**B.** The asymmetry among selected studies exhibits on the contour-enhanced funnel plots used for publication bias assessment of AMR prevalence of *Campylobacter* isolates against Ampicillin - AMP (B1), Azithromycin - AZI (B2), Ciprofloxacin - CIP (B3), Erythromycin - ERY (B4), Gentamicin - GEN (B5), Nalidixic acid - NAL (B6), Sulfamethoxazole-Trimethoprim - SXT (B7), and Tetracycline - TET (B8). Each scatter illustrates each selected article. The dashed vertical line shows the average effect sizes. The shaded regions illustrate the statistical significance of asymmetry of the publication (*p* < 0.1 (in gray), *p* < 0.05 (in dark yellow), *p* < 0.01(in yellow)).

**B1. Ampicillin**

| **Categories** | **No. prevalence estimates** | **Contour-enhanced funnel plots** | **Egger’s test** | |
| --- | --- | --- | --- | --- |
|  |  |  | ***βo*** | ***P-value*** |
| ***C. jejuni*** |  |  |  |  |
| General population (diarrhea) | 2 | 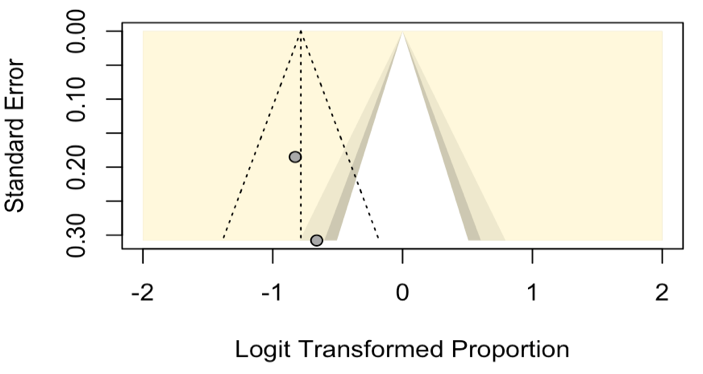 | nc | nc |
| Chicken | 4 | 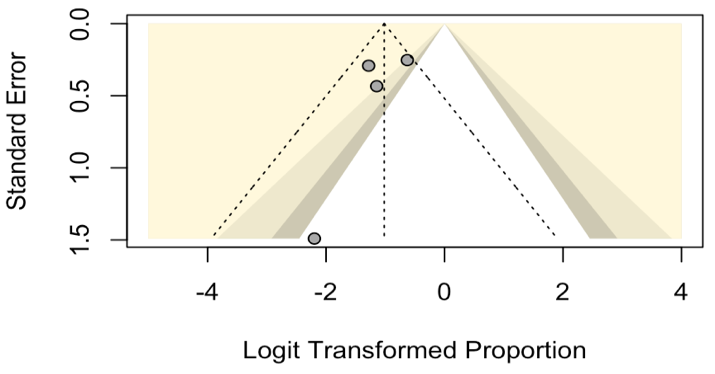 | -1.04 | 0.409 |
| Chicken products | 2 | 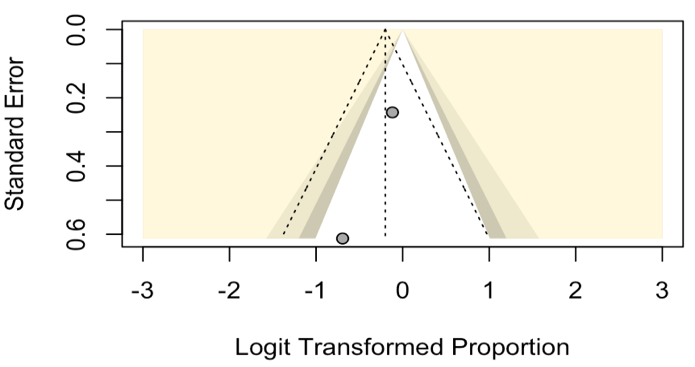 | nc | nc |

**B2. Azithromycin**

| **Categories** | **No. prevalence estimates** | **Contour-enhanced funnel plots** | **Egger’s test** | |
| --- | --- | --- | --- | --- |
|  |  |  | ***βo*** | ***P-value*** |
| ***C. jejuni*** |  |  |  |  |
| Children (diarrhea) | 3 | 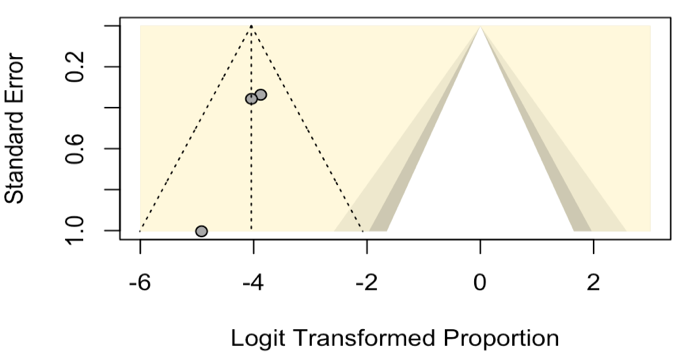 | -3.52 | 0.176 |
| General population (diarrhea) | 3 | 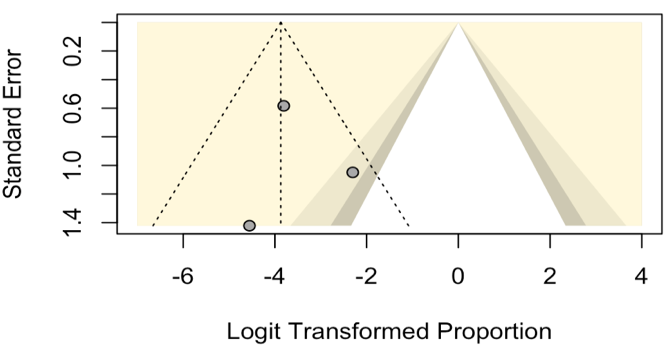 | 0.15 | 0.905 |
| ***C. coli*** |  |  | -0.66 | 0.629 |
| Children (diarrhea) | 3 | 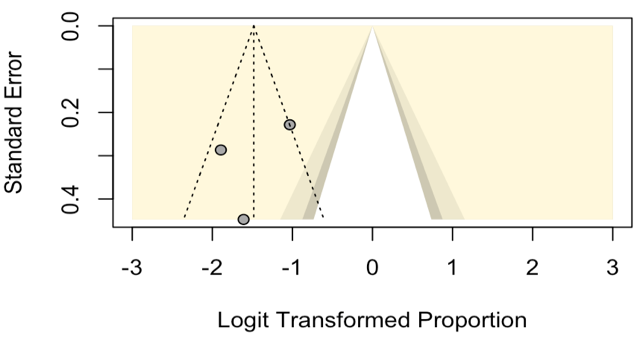 |  |  |

**B3. Ciprofloxacin**

| **Categories** | **No. prevalence estimates** | **Contour-enhanced funnel plots** | **Egger’s test** | |
| --- | --- | --- | --- | --- |
|  |  |  | ***βo*** | ***P-value*** |
| ***C. jejuni*** |  |  |  |  |
| Children (diarrhea) | 4 | 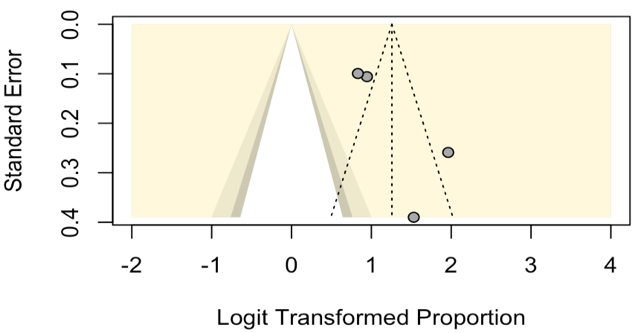 | 2.34 | 0.144 |
| General population (diarrhea) | 3 | 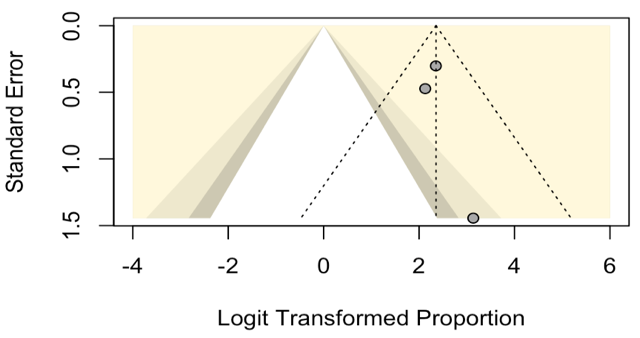 | 0.66 | 0.630 |
| Chicken | 6 | 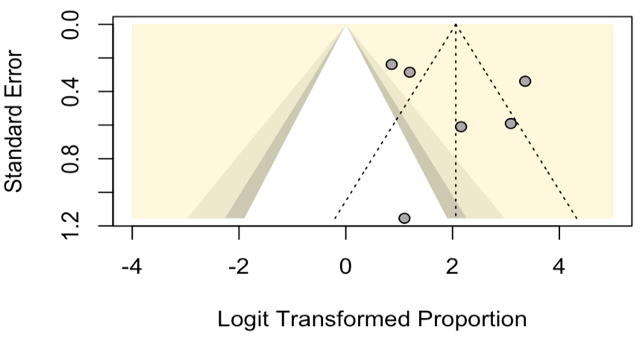 | 0.85 | 0.441 |
| Chicken products | 3 | 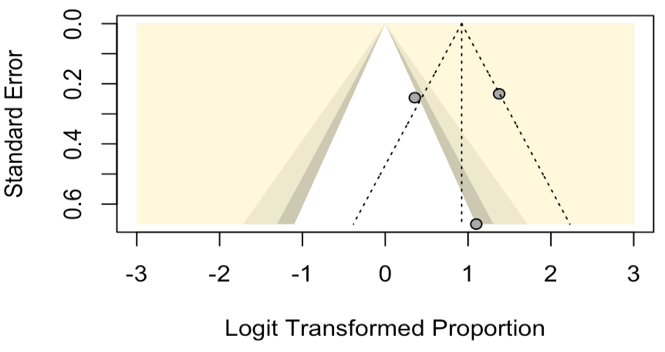 | 0.04 | 0.976 |
| ***C. coli*** |  |  |  |  |
| Children (diarrhea) | 3 | 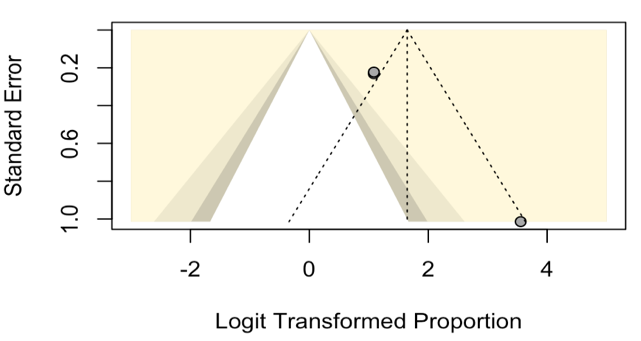 | 7.11 | 0.024 |

**B4. Erythromycin**

| **Categories** | **No. prevalence estimates** | **Contour-enhanced funnel plots** | **Egger’s test** | |
| --- | --- | --- | --- | --- |
|  |  |  | ***βo*** | ***P-value*** |
| ***C. jejuni*** |  |  |  |  |
| Children (diarrhea) | 4 | 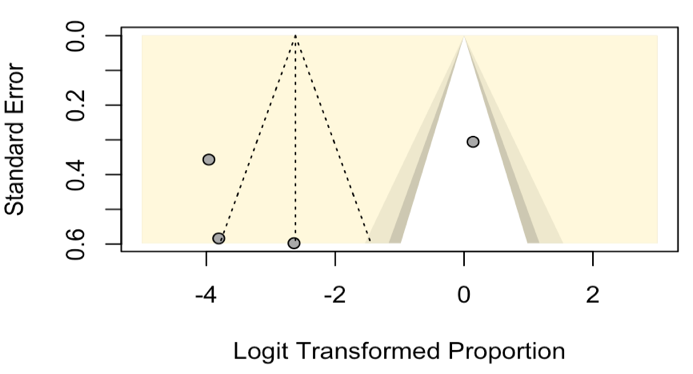 | -0.88 | 0.471 |
| General population (diarrhea) | 2 | 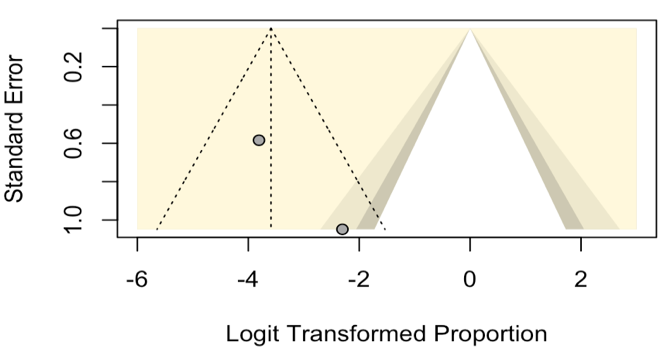 | nc | nc |
| Chicken | 5 | 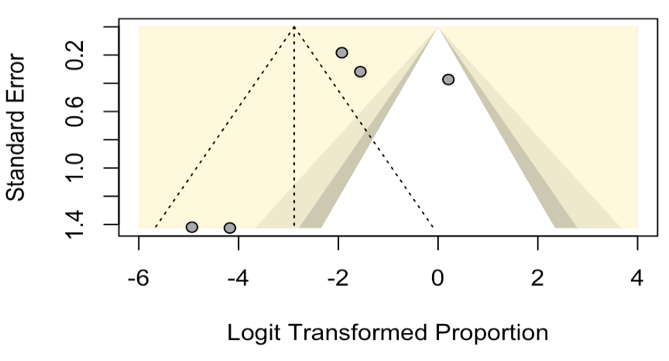 | -0.27 | 0.807 |
| Chicken products | 3 | 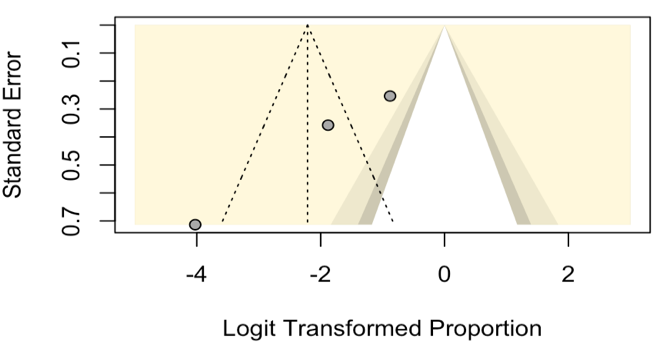 | -6.67 | 0.095 |
| ***C. coli*** |  |  |  |  |
| Children (diarrhea) | 3 | 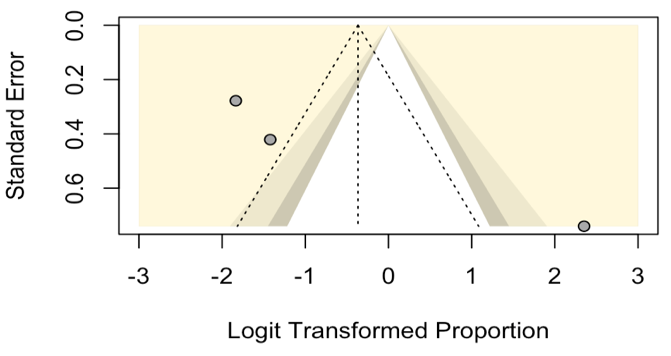 | 2.93 | 0.209 |
| Chicken | 2 | 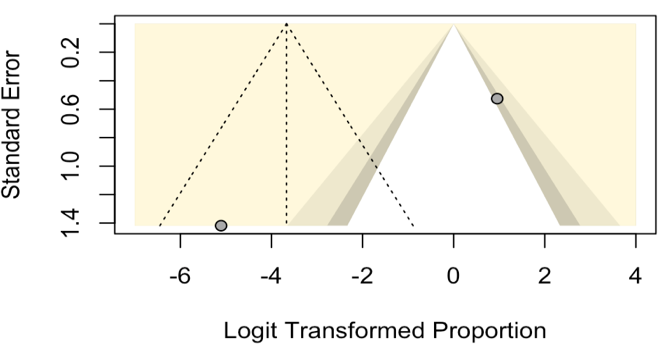 | nc | nc |

**B5. Gentamicin**

| **Categories** | **No. prevalence estimates** | **Contour-enhanced funnel plots** | **Egger’s test** | |
| --- | --- | --- | --- | --- |
|  |  |  | ***βo*** | ***P-value*** |
| ***C. jejuni*** |  |  |  |  |
| Chicken | 2 | 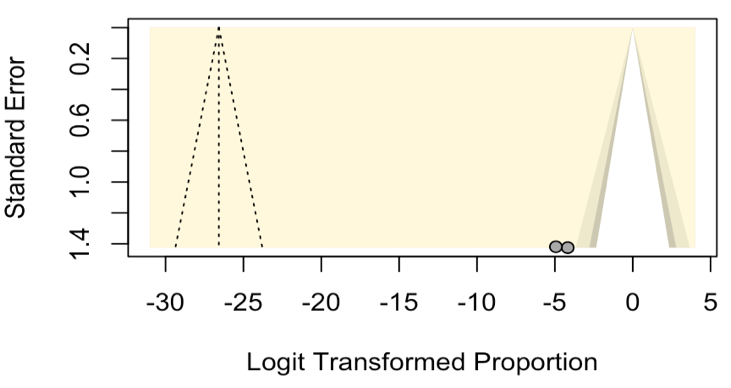 | nc | nc |
| Chicken products | 2 | 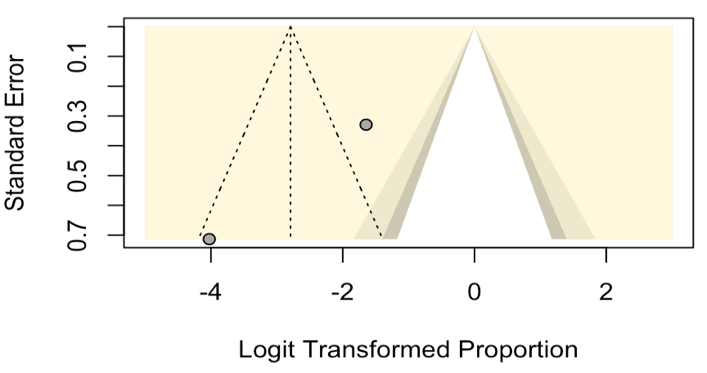 | nc | nc |

**B6. Nalidixic acid**

| **Categories** | **No. prevalence estimates** | **Contour-enhanced funnel plots** | **Egger’s test** | |
| --- | --- | --- | --- | --- |
|  |  |  | ***βo*** | ***P-value*** |
| ***C. jejuni*** |  |  |  |  |
| Children (diarrhea) | 4 | 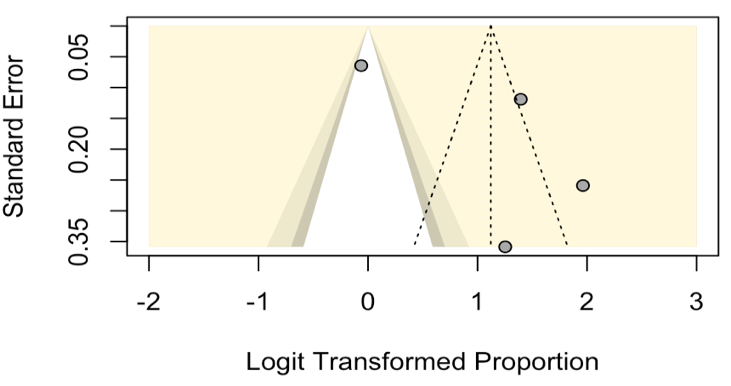 | 1.65 | 0.241 |
| General population (diarrhea) | 3 | 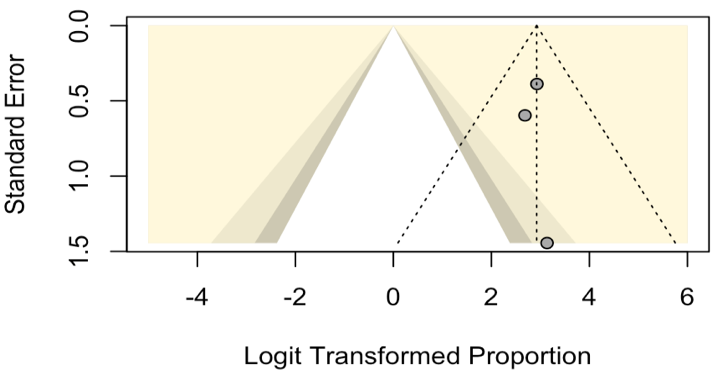 | 0.09 | 0.942 |
| Chicken | 4 | 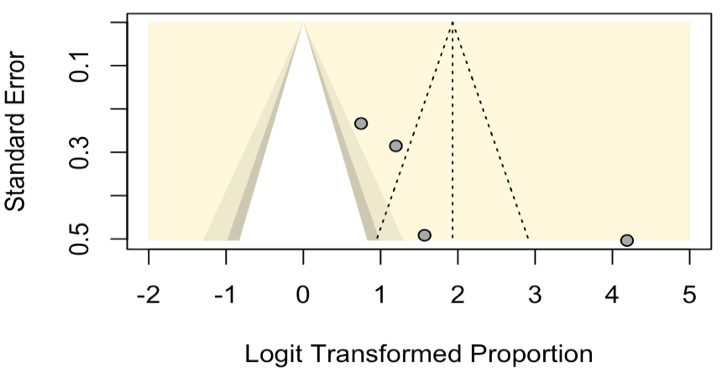 | 2.01 | 0.182 |
| Chicken products | 2 | 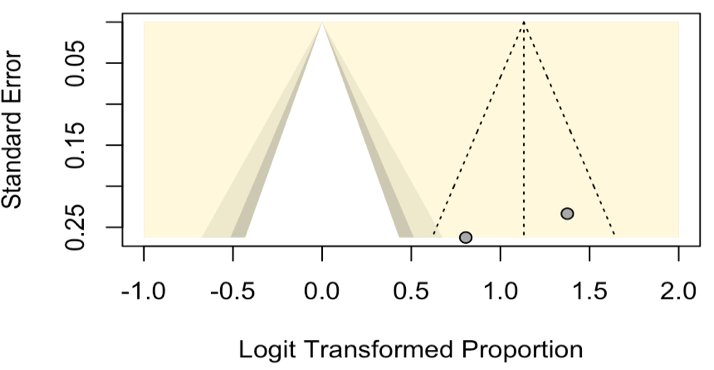 | nc | nc |
| ***C. coli*** |  |  |  |  |
| Children (diarrhea) | 3 | 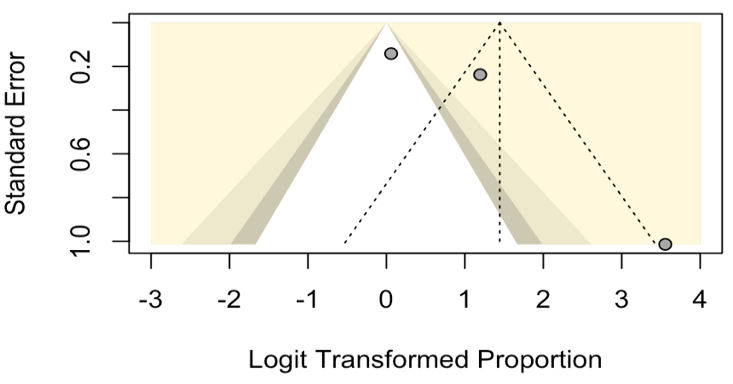 | 1.74 | 0.332 |

**B7. Trimethoprim-Sulfamethoxazole**

| **Categories** | **No. prevalence estimates** | **Contour-enhanced funnel plots** | **Egger’s test** | |
| --- | --- | --- | --- | --- |
|  |  |  | ***βo*** | ***P-value*** |
| ***C. jejuni*** |  |  |  |  |
| General population (diarrhea) | 2 | 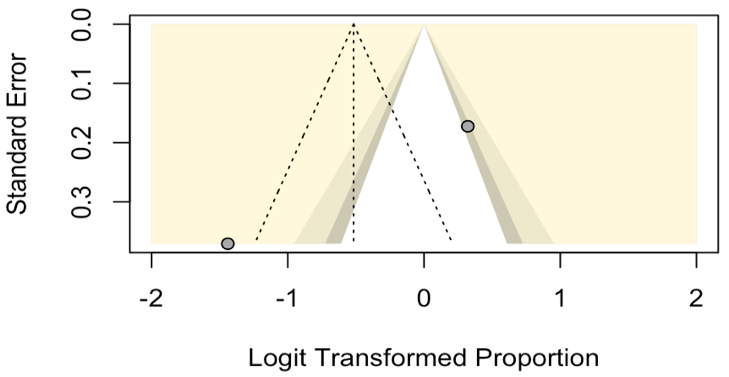 | nc | nc |
| Chicken | 2 | 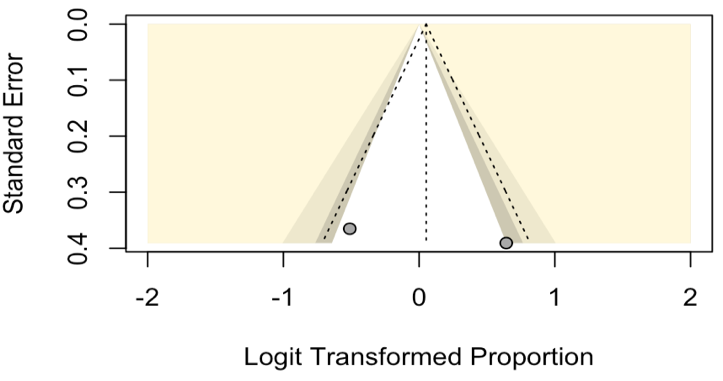 | nc | nc |

**B8. Tetracycline**

| **Categories** | **No. prevalence estimates** | **Contour-enhanced funnel plots** | **Egger’s test** | |
| --- | --- | --- | --- | --- |
|  |  |  | ***βo*** | ***P-value*** |
| ***C. jejuni*** |  |  |  |  |
| Children (diarrhea) | 2 | 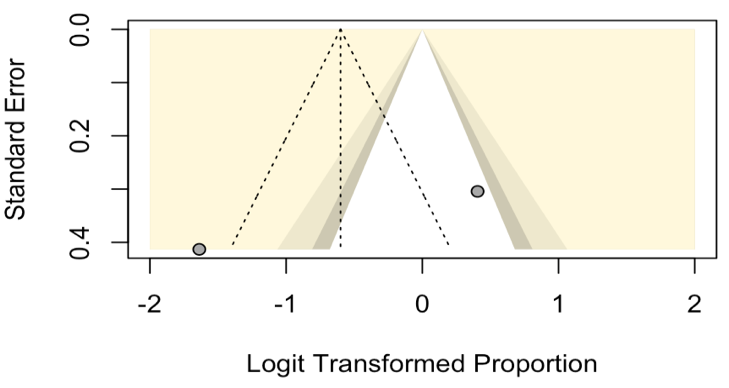 | nc | nc |
| General population (diarrhea) | 2 | 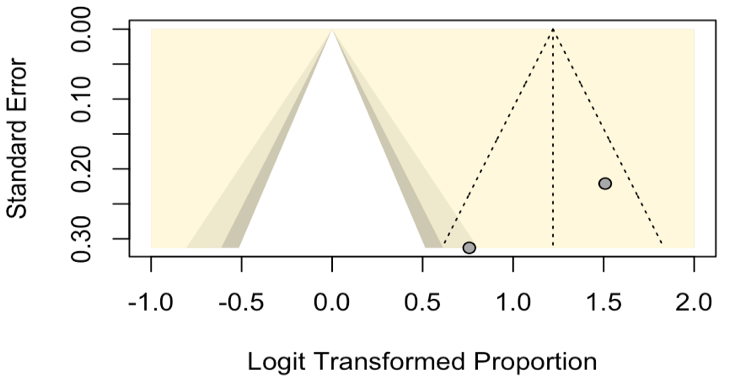 | nc | nc |
| Chicken | 6 | 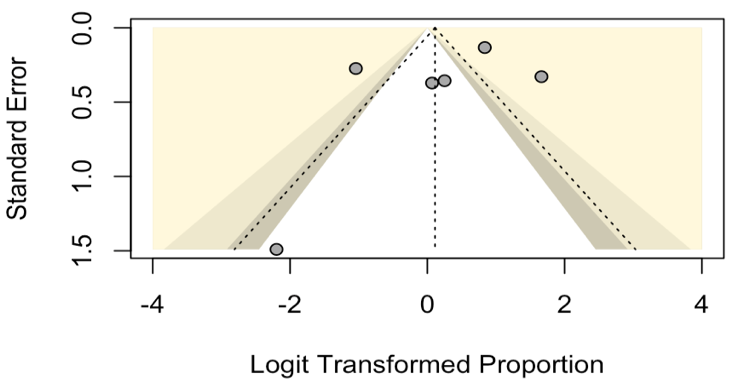 | -0.89 | 0.422 |
| Chicken products | 3 | 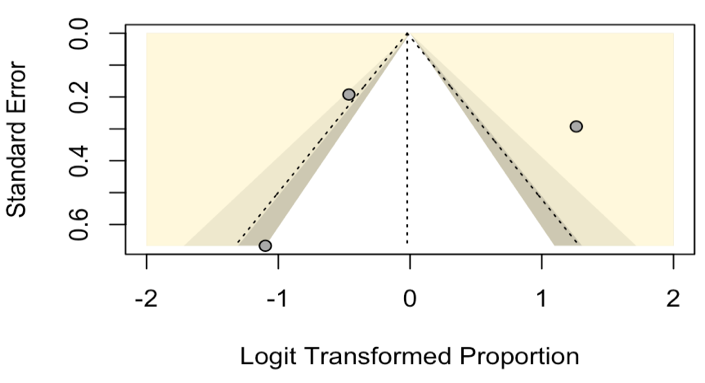 | 0.09 | 0.945 |
| ***C. coli*** |  |  |  |  |
| Chicken | 2 | 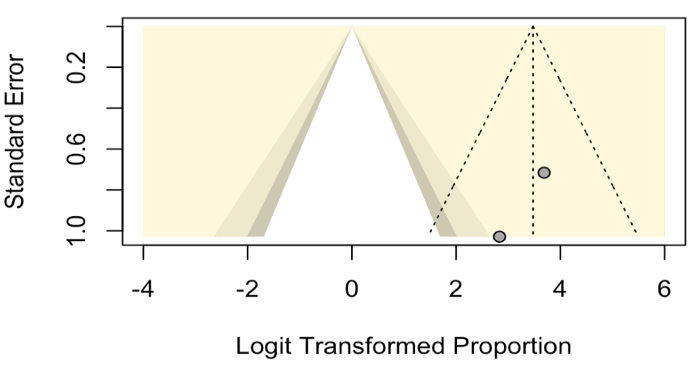 | nc | nc |
