# Supplementary material for: Prevalence and antimicrobial resistance of Campylobacter jejuni and Campylobacter coli over time in Thailand under a One Health approach: A systematic review and meta-analysis
Source: One Health. 2025 Jan 10;20:100965. doi: 10.1016/j.onehlt.2025.100965 (PMC11782884; doi:10.1016/j.onehlt.2025.100965)
Supplement: Supplementary Table 1 — The PRISMA 2020 item checklist. [file mmc4.docx]

| **Section and Topic** | **Item #** | **Checklist item** | **Content presented in the manuscript** |
| --- | --- | --- | --- |
| **TITLE** | | |  |
| Title | 1 | Identify the report as a systematic review. | Prevalence and Antimicrobial Resistance of *Campylobacter* spp. over time in Thailand under One Health approach: A systematic review and meta-analyses. |
| **ABSTRACT** | | |  |
| Abstract | 2 | See the PRISMA 2020 for Abstracts checklist. | Based on the PRISMA guidelines, we retrieved 81 articles from PubMed, ScienceDirect, and Google Scholar for review. |
| **INTRODUCTION** | | |  |
| Rationale | 3 | Describe the rationale for the review in the context of existing knowledge. | *Campylobacter* spp. is a prominent zoonotic pathogen causing human gastrointestinal infections through foodborne transmission. Although the burden of campylobacteriosis still critically impacts human health and food safety, *Campylobacter* spp. has been neglected for the past decades, especially being underestimated in low- and middle-income countries (LMICs).  Like other Southeast Asia countries, Thailand is recognized for having a substantial burden of *Campylobacter* infections in the region. Besides, Thailand is known as a hotspot of antimicrobial resistance (AMR).  Several reviews on the prevalence and AMR of *Campylobacter* spp. have been conducted in Thailand. Therefore, an updated review is essential to investigate the prevalence of *Campylobacter* spp. and comprehensively explore AMR trends in Thailand under the One Health approach, over across human, animal, and environmental compartments. |
| Objectives | 4 | Provide an explicit statement of the objective(s) or question(s) the review addresses. | Our study aimed to investigate the prevalence, sequence types, and phenotypic and genotypic AMR of *Campylobacter* spp. in Thailand using the One Health approach. Additionally, we evaluated the trends in *Campylobacter* prevalence and the AMR issues over time in Thailand. |
| **METHODS** | | |  |
| Eligibility criteria | 5 | Specify the inclusion and exclusion criteria for the review and how studies were grouped for the syntheses. | The selection of articles for review was completed based on three stages: title, abstract and full text. The title and abstracts were screened for information on: (1) Prevalence of *Campylobacter* isolates detected; (2) Sequence types (STs); (3) Antimicrobial susceptibility; and (4) Antimicrobial resistance genes (ARGs)/mutations. |
| Information sources | 6 | Specify all databases, registers, websites, organisations, reference lists and other sources searched or consulted to identify studies. Specify the date when each source was last searched or consulted. | Initially, searching through PubMed, ScienceDirect and Google Scholar were performed and yielded the list of all titles and abstracts of relevant articles. The time for searching was performed in November 2023. (PubMed: 02/11/2023), ScienceDirect (17/11/2023) and Google Scholar (30/11/2023). |
| Search strategy | 7 | Present the full search strategies for all databases, registers and websites, including any filters and limits used. | We searched PubMed, ScienceDirect for titles containing the terms: ‘*Campylobacter*’, ‘Campylobacteriosis’, ‘Thai’, and ‘Thailand’. Moreover, hand-searching from the reference lists of selected studies were also performed on Google Scholar to have a higher range of eligible articles. |
| Selection process | 8 | Specify the methods used to decide whether a study met the inclusion criteria of the review, including how many reviewers screened each record and each report retrieved, whether they worked independently, and if applicable, details of automation tools used in the process. | Article selection for the review was conducted based on three stages: title, abstract and full text. Initially, searches were performed on PubMed, ScienceDirect and Google Scholar, yielding a list of all titles of relevant articles. For inclusion, the articles were selected if their title and abstracts provided information on: (1) prevalence of *Campylobacter* isolates detected; (2) sequence types (STs); (3) antimicrobial susceptibility; and (4) antimicrobial resistance genes (ARGs)/mutations. During the full-text screening, articles included had to describe the prevalence of *Campylobacter* spp., and sample collection was from: (1) humans (carriage/ diarrhea); (2) animals (e.g., chickens, ducks, pigs, ruminants, aquatic animals); (3) animal products (e.g., products derived from chickens, ducks, pigs, ruminants and seafood); and (4) non-animal derived products (e.g., salad, fruit, vegetable); and (5) environmental samples (e.g., litter, footwear, water, feed). For exclusion, articles without any information on either the *Campylobater* spp. prevalence or STs or antimicrobial susceptibility or detection of ARGs/mutations were excluded. Review articles, book chapter, conference abstracts, letter, and articles written in other languages than English, and duplicated among records were also excluded.  Article selection was based on the Joanna Briggs Institute (JBI) Critical Appraisal Checklist, specifically designed for studies reporting prevalence data. The two authors (D.H.P and T.T.N) independently conducted the selection process. In cases where discrepancies in study selection between the two reviewers, a third reviewer (T.P) participated in to resolve any disagreements regarding article inclusion. |
| Data collection process | 9 | Specify the methods used to collect data from reports, including how many reviewers collected data from each report, whether they worked independently, any processes for obtaining or confirming data from study investigators, and if applicable, details of automation tools used in the process. | Two authors (D.H.P and T.T.N) independently participated in data extraction and cross-checked the extracted data for consistency to mitigate the information bias. In case of discrepancies during data extraction, the third reviewer (T.P) was responsible for making a final decision. |
| Data items | 10a | List and define all outcomes for which data were sought. Specify whether all results that were compatible with each outcome domain in each study were sought (e.g. for all measures, time points, analyses), and if not, the methods used to decide which results to collect. | From each study, the information was extracted as follows:  (1) Year of sample collection;  (2) Sources of samples collected (humans, animals, animal derived products, non-animal derived product, and environmental samples);  (3) Sample type (faeces, stool, rectal swabs, blood, floor swabs, farm waste, carcass, meat, milk, environmental samples);  (4) Number of samples collected;  (5) Methods of *Campylobacter* spp. identification;  (6) Prevalence of *Campylobacter* spp.;  (7) Typing methods of *Campylobacter* spp.;  (8) STs using multilocus sequence typing (MLST);  (8) Methods of antimicrobial susceptibility testing;  (9) Prevalence of phenotypic resistance;  (10) Genotypic resistance (ARGs and mutations). |
|  | 10b | List and define all other variables for which data were sought (e.g., participant and intervention characteristics, funding sources). Describe any assumptions made about any missing or unclear information. | In case the absence of an explicit date of sample collection, a date of two years prior to publication was assigned; for studies conducted over a time period, a mid-point was defined.  Human isolates were further categorized based on whether *Campylobacter* isolates from healthy subjects (i.e., enteric carriage), or from cases of enteric disease (i.e., diarrhea). Animal isolates were also classified based on the different animal species including poultry, pig, aquatic animals, and ruminant. |
| Study risk of bias assessment | 11 | Specify the methods used to assess risk of bias in the included studies, including details of the tool(s) used, how many reviewers assessed each study and whether they worked independently, and if applicable, details of automation tools used in the process. | - Selection bias: Article selection was based on the Joanna Briggs Institute (JBI) Critical Appraisal Checklist, specifically designed for studies reporting prevalence data. The two authors (D.H.P and T.T.N) independently conducted the selection process. In cases where discrepancies in study selection between the two reviewers, a third reviewer (T.P) participated in to resolve any disagreements regarding article inclusion.  - Information bias: Two authors (D.H.P and T.T.N) independently participated in data extraction and cross-checked the extracted data for consistency to mitigate the information bias. In case of discrepancies during data extraction, the third reviewer (T.P) was responsible for making a final decision.  - Publication bias: Two methods were used to assess publication bias in the meta-analysis. The contour-enhanced funnel plots were used to visualize the asymmetrical patterns indicating publication bias. Subsequently, Egger’s regression test was performed based on the linear regression model to identify the publication bias by testing for asymmetry in the funnel plots. In case the intercept (*βo*) deviated from zero, indicating funnel plot asymmetry and suggesting the presence of publication bias. |
| Effect measures | 12 | Specify for each outcome the effect measure(s) (e.g. risk ratio, mean difference) used in the synthesis or presentation of results. | The extracted data was synthesized and evaluated through descriptive analyses, including proportion, mean, and standard error (SE).  + Prevalence of *Campylobacter* isolates detected (%)  + Prevalence Antimicrobial resistance genes (ARGs)/mutations (%) |
| Synthesis methods | 13a | Describe the processes used to decide which studies were eligible for each synthesis (e.g. tabulating the study intervention characteristics and comparing against the planned groups for each synthesis (item #5). | Meta-analysis was performed from included studies with the prevalence data extracted from at least two articles by the sources of isolates categorized as follows: (1) humans with enteric carriage. (2) humans with diarrhea, (3) poultry/poultry meat; (4) pigs/pork; (5) aquatic animals/seafood; (6) ruminant/ ruminant products; and (7) environmental samples. |
|  | 13b | Describe any methods required to prepare the data for presentation or synthesis, such as handling of missing summary statistics, or data conversions. | Descriptive analyses of proportion, mean, and standard error (SE). |
|  | 13c | Describe any methods used to tabulate or visually display results of individual studies and syntheses. | Descriptive analyses of proportion, mean, and standard error (SE). |
|  | 13d | Describe any methods used to synthesize results and provide a rationale for the choice(s). If meta-analysis was performed, describe the model(s), method(s) to identify the presence and extent of statistical heterogeneity, and software package(s) used. | Logit-transformed proportions were analyzed using a generalized linear mixed-effect model, including a random-effect model to identify the within- and between-study variances. The results of the meta-analysis were presented using forest plots. Univariable meta-regression models were performed to investigate the trends in *Campylobacter* prevalence and phenotypic resistance.  All statistical analyses and figures were performed using R programming language with the ‘*meta*’ and ‘*meta for*’ packages were used for meta-analysis and univariable meta-regression models. Package ‘*tidyverse*’ facilitated the evaluation of publication bias. Also, package ‘*ggplot2*’ were used to visualize the study results. Moreover, all STs reported were visualized by constructing a minimum spanning tree using the goeBURST algorithm in the PHYLOViZ software (<http://www.phyloviz.net/>). |
|  | 13e | Describe any methods used to explore possible causes of heterogeneity among study results (e.g. subgroup analysis, meta-regression). | Heterogeneity across selected studies was assessed using the inverse variance index (*I^2^*^)^, with *I^2^* > 75%, p-value < 0.05 indicating significant heterogeneity, as described by previous studies. |
|  | 13f | Describe any sensitivity analyses conducted to assess robustness of the synthesized results. | Additionally, sensitivity analyses were performed to assess the impact of influential studies with data extracted based on sampling year assumptions. The results of the main analysis which included all studies (both those with data collected using assumptions and those without) were compared with the results after removing influential studies. |
| Reporting bias assessment | 14 | Describe any methods used to assess risk of bias due to missing results in a synthesis (arising from reporting biases). | Two methods were used to assess publication bias in the meta-analysis. The contour-enhanced funnel plots were used to visualize the asymmetrical patterns indicating publication bias. Subsequently, Egger’s regression test was performed based on the linear regression model to identify the publication bias by testing for asymmetry in the funnel plots. In case the intercept (*βo*) deviated from zero, indicating funnel plot asymmetry and suggesting the presence of publication bias. |
| Certainty assessment | 15 | Describe any methods used to assess certainty (or confidence) in the body of evidence for an outcome. | Not applicable |
| **RESULTS** | | |  |
| Study selection | 16a | Describe the results of the search and selection process, from the number of records identified in the search to the number of studies included in the review, ideally using a flow diagram. | A total of 480 articles were identified during the initial search. Of these papers, 286 articles that met the criterion for publishing primary data were selected. Additionally, 91 articles were excluded due to the absence of *Campylobacter* data in their title and abstracts. Consequently, 195 papers remained for evaluation within the full text of the publication. Subsequently, a further refinement process was undertaken by excluding 39 articles duplicated among the databases, 78 articles conducted in countries other than Thailand, 6 articles without full-text availability, and 17 articles lacking data on *Campylobacter* prevalence, STs, phenotypic and genotypic antimicrobial resistance. Besides, through hand-searching on Google Scholar, 19 eligible articles were further included, resulting in a total of 81 articles for the systematic review. Additionally, to conduct meta-analyses, articles reporting the prevalence of *Campylobacter* isolates (n=58) and the phenotypic antimicrobial resistance (n=20) were chosen for meta-analyses. |
|  | 16b | Cite studies that might appear to meet the inclusion criteria, but which were excluded, and explain why they were excluded. | In Table 1. The selected articles for review have been cited: ^1^ 13 studies [22,35-46]; ^2^ 3 studies [10,12,47]; ^3^ 9 studies [48-56]; ^4^ 2 studies [57,58]; ^5^ 4 studies [11,59-61]; ^6^ 3 studies [62-64]; ^7^ 1 study [65]; ^8^ 8 studies [66-73]; ^9^ 2 studies [74,75]; ^10^ 1 study [76]; ^11^ 1 study [77]; ^12^ 1 study [78]; ^13^ 10 studies [79-88]; ^14^ 1 study [89]; ^15^ 1 study [90]; ^16^ 1 study [91]; ^17^ 1 study [92]; ^18^ 4 studies [93-96]; ^19^ 2 studies [97,98]; ^20^ 2 studies [99,100]; ^21^ 2 studies [101,102]; ^22^ 2 studies [103,104]; ^23^ 2 studies [105,106]; ^24^ 1 study [107]; ^25^ 1 study [108]; ^26^ 1 study [109]; ^27^ 1 study [110]; ^28^ 1 study [111]. |
| Study characteristics | 17 | Cite each included study and present its characteristics. | [48-56]; ^4^ 2 studies [57,58]; ^5^ 4 studies [11,59-61]; ^6^ 3 studies [62-64]; ^7^ 1 study [65]; ^8^ 8 studies [66-73]; ^9^ 2 studies [74,75]; ^10^ 1 study [76]; ^11^ 1 study [77]; ^12^ 1 study [78]; ^13^ 10 studies [79-88]; ^14^ 1 study [89]; ^15^ 1 study [90]; ^16^ 1 study [91]; ^17^ 1 study [92]; ^18^ 4 studies [93-96]; ^19^ 2 studies [97,98]; ^20^ 2 studies [99,100]; ^21^ 2 studies [101,102]; ^22^ 2 studies [103,104]; ^23^ 2 studies [105,106]; ^24^ 1 study [107]; ^25^ 1 study [108]; ^26^ 1 study [109]; ^27^ 1 study [110]; ^28^ 1 study [111]. |
| Risk of bias in studies | 18 | Present assessments of risk of bias for each included study. | The contour-enhanced funnel plots illustrate asymmetry among the selected articles in the meta-analysis of *Campylobacter* prevalence (n=60 studies, Figure 6A, 6B) and the AMR prevalence of *Campylobacter* spp. (n=20 studies, Figure 6C-6J). |
| Results of individual studies | 19 | For all outcomes, present, for each study: (a) summary statistics for each group (where appropriate) and (b) an effect estimate and its precision (e.g. confidence/credible interval), ideally using structured tables or plots. | *3.2. Characteristics of selected study*  Of the 81 selected studies, 35 (43.2%) included *Campylobacter* spp. isolated from humans: thirteen focused on enteric diseases in children with diarrhea, nine on enteric diseases in the general population, four on both enteric disease and carriage in the general population, three on both enteric disease and carriage in children, and four on humans without specifying the age group. A total of 13 studies (16.0%) investigated *Campylobacter* spp. in animals, specifically chickens (8 studies), ducks (2 studies), pigs (1 study), ruminants (1 study), and a combination of pigs and ruminants (1 study). Regarding animal products, 13 studies (16.0%) were conducted, in which most focused on samples collected from chicken products (10 studies), while others investigated *Campylobacter* spp. in mixed samples from chicken, pork, ruminants, and seafood. Only one study was categorized as non-animal-derived products. Additionally, nineteen studies were classified as integrated studies, as they collected samples from various sources, including humans, animals, animal products, and environmental samples.  Most human studies were conducted from 1985 to 2005 (26/35 studies), while studies on animals (13/13 studies) and animal products (12/13 studies) were performed from 2001 to 2023. Regarding the study area, most (45/81) studies were conducted in the central region, in which 13/45 studies were conducted in multi-regions, including central and other parts of Thailand, and 25/45 studies were conducted in Bangkok. For other regions, most studies were conducted in some core provinces like Chiangmai (13 studies, North), Khon Kaen (8 studies, Northeast), Nakhon Si Thammarat (5 studies, South), Kanchanaburi (5 studies, West), Chonburi (4 studies, East) **(Figure 2)**.  Regarding *Campylobacter* identification, PCR was the most common method to identify *Campylobacter* spp., used in 38 studies (46.9% studies). Twenty-eight studies performed typing methods, including multilocus sequence typing (MLST) (8 studies), and pulsed-field gel electrophoresis (PFGE) (5 studies), whereas most studies conducted before 2000 used traditional method such as Lior and Penner serotyping. In 35 studies (43.2%), *Campylobacter* isolates were investigated for their antimicrobial susceptibility. The disc diffusion test was used in 12 studies, while 21 studies applied the minimum inhibitory concentration (MIC). Only 8 studies (9.9%) performed the genotypic antimicrobial resistance to detect antimicrobial resistance genes (ARGs) and mutations. Full details are given in **Table 1** and **Supplementary File 2.** |
| Results of syntheses | 20a | For each synthesis, briefly summarise the characteristics and risk of bias among contributing studies. | Among the various sources of sample collection in studies on C. jejuni, the highest prevalence was observed in chicken, with a pooled prevalence of 43.6% (crude prevalence 48.4%), followed by chicken products at 31.4% (crude prevalence 38.1%), ducks at 16.7% (crude prevalence 17.6%), the general population with diarrhea at 12.0% (crude prevalence 21.0%), and children with diarrhea at 12.0% (crude prevalence 14.9%). Other categories had low prevalence levels, all below 2.6%. In environmental samples, the pooled prevalence of C. jejuni was 17.1% (crude prevalence 16.6%) at duck farms and 6.7% (crude prevalence 17.5%) at chicken farms. Similarly, the pooled prevalence of was observed highest in sample related to chicken, with the *C. coli* prevalence levels at chicken farms with environmental samples were 12.6% (crude prevalence 17.5%), chicken products 10.4% (crude prevalence 13.3%), and chicken 10.30% (crude prevalence 8.9%). Other categories were found with the low prevalence levels, all below 7.4%.  The highest AMR prevalence in Campylobacter spp. was observed for quinolone antimicrobials across all categories. In children with diarrhea, C. jejuni isolates were resistant to CIP and NAL at 77.9% and 75.4%, respectively, while in C. coli, the resistance levels were at 83.3% and 80.9%. In the general population age group, resistance to CIP and NAL was 91.3% and 94.8%, respectively. In isolates related to chicken sources, the resistance to CIP and NAL of C. jejuni was 88.7% and 87.3% in chicken, and 71.6% and 75.6% in chicken products. In macrolide antimicrobials, C. jejuni isolates showed high susceptibility to ERY and AZI, with resistance levels below 6.8% across all human-related categories and 9.9% in chicken-related categories. However, C. coli from children with diarrhea exhibited resistance levels of 40.9% to ERY and 18.5% to AZI. The AMR levels of C. jejuni to TET ranged from 35.4% to 77.2% across all categories, while C. coli resistance was only observed in chickens at 97.0%. For AMP and SXT, the levels of AMR prevalence of only *C. jejuni* were recorded, ranged from 26.5% to 45.0% for AMR, and 37.4 to 51.3 for SXT, respectively. Only chicken studies reported the prevalence of *Campylobacter* isolates to GEN, with the prevalence at 5.8% in chicken products and 0.0% in chicken (**Supplementary Figure 2**). |
|  | 20b | Present results of all statistical syntheses conducted. If meta-analysis was done, present for each the summary estimate and its precision (e.g. confidence/credible interval) and measures of statistical heterogeneity. If comparing groups, describe the direction of the effect. | Forest plots were applied to visualize the findings of meta-analysis. The results were presented in Table 2, Table 3 and Supplementary Figure 1, Supplementary Figure 2. |
|  | 20c | Present results of all investigations of possible causes of heterogeneity among study results. | The factors may contribute to the high heterogeneity of selected study as follows:  - Wide range of year of sample collection (from 1985 – 2023)  - Multiple host species: Humans, animals, animal product, non-animal derived products, and the environment  - Sample type (faeces, stool, rectal swabs, blood, floor swabs, farm waste, carcass, meat, milk, environmental samples);  - Number of samples collected (small studies with limitation of sample size were observed)  - Methods of *Campylobacter* spp. identification (culture-based, PCR, biochemical test…) |
|  | 20d | Present results of all sensitivity analyses conducted to assess the robustness of the synthesized results. | Sensitivity analyses generally aligned with the main analyses, except in studies on children with diarrhea. Initially, seven studies in this category showed no significant trends in *Campylobacter* prevalence over time. However, after removing an influential study, the trend became significant (**Supplementary File 3**).  Our sensitivity analyses found that removing influential studies resulted in non-significant outcomes in the meta-analyses due to the limited number of studies. Therefore, to preserve our findings' statistical power and significance, we decided to include all studies (n=20), both with and without assumptions, in our final analyses (**Supplementary File 7**). |
| Reporting biases | 21 | Present assessments of risk of bias due to missing results (arising from reporting biases) for each synthesis assessed. | The scattered points representing selected articles are unevenly distributed and situated far from the pooled effect size (vertical line). Most scatters were plotted within the shaded regions of p < 0.05 and p < 0.01, indicating the significant asymmetry of selected articles in meta-analysis.  The asymmetry observed in the funnel plots was further supported by the results of Egger's regression tests. The intercepts (*βo*) of meta-analysis involving *Campylobacter* spp. in humans, AMP, AZI, CIP, ERY, GEN, NAL differ from zero (all *βo* either < -1.232 or > 1.942). Conversely, in studies reporting data on *Campylobacter* prevalence in animals (*βo* = -0.620), and AMR prevalence to SXT (*βo* = 0.037) and TET (*βo* = 0.007), although the intercept of Egger's regression tests was closed to zero, no significance was detected (all p > 0.763). |
| Certainty of evidence | 22 | Present assessments of certainty (or confidence) in the body of evidence for each outcome assessed. | Not applicable |
| **DISCUSSION** | | |  |
| Discussion | 23a | Provide a general interpretation of the results in the context of other evidence. | The general interpretation of the results are presented in Discussion part, Page 26 – Page 29 |
|  | 23b | Discuss any limitations of the evidence included in the review. | Our study acknowledges several limitations. Given that our review investigated the prevalence and AMR of *Campylobacter* isolates across humans, animals, and the environment; however, the AMR prevalence was exclusively observed in isolates obtained from diarrhea in humans and poultry without covering other animal species. Besides, despite efforts to minimize biases through study selection, data extraction, and publication bias assessment, the comprehensive nature of our review and the extensive study period under the One Health approach unavoidably introduced high heterogeneity among selected studies. The observed high heterogeneity may be influenced by publication bias stemming from the impact of small studies with limited sample collection in our review, possibly posing challenges in accurately assessing the representativeness of the AMR situation of *Campylobacter* spp. throughout the entire country. Nevertheless, within these acknowledged limitations, our study provides valuable insights into *Campylobacter* prevalence and its AMR trends in Thailand under the One Health approach, offering a potential model for application in other LMICs. |
|  | 23c | Discuss any limitations of the review processes used. | Secondly, although sensitivity analyses were conducted to assess the impact of studies using assumed sampling years, the meta-analysis included only a small number of studies due to the diverse classification of sample sources. Therefore, to ensure adequate studies, we accepted these assumptions in some parts of our meta-analysis |
|  | 23d | Discuss implications of the results for practice, policy, and future research. | Our observations emphasize the critical need for ongoing monitoring and the implementation of preventive measures in both poultry farming and human health to mitigate the impact of *Campylobacter* effectively.  Since MLST is a crucial tool applied to understand the genetic diversity, molecular epidemiology, and disease surveillance of microorganisms, it is suggested that further studies on the MLST profiles in *C. coli*and other animal species to have a comprehensive picture of genetic diversity and the evolution of *Campylobacter* spp. in Thailand.  Following the Thailand National Strategic Plans in regulation of antimicrobial distribution on AMR [111], legislative measures are recommended to restrict the use of fluoroquinolones and macrolides in animal production systems in Thailand. Besides, further investigations are also suggested to explore the association between antimicrobial use and AMR in bacterial isolates.  It is crucial to utilize both observable and genetic approaches, with a preference for sequence-based genotyping, to detect and monitor the AMR of *Campylobacter* spp. effectively. |
| **OTHER INFORMATION** | | |  |
| Registration and protocol | 24a | Provide registration information for the review, including register name and registration number, or state that the review was not registered. | Not applicable |
|  | 24b | Indicate where the review protocol can be accessed, or state that a protocol was not prepared. | Not applicable |
|  | 24c | Describe and explain any amendments to information provided at registration or in the protocol. | Not applicable |
| Support | 25 | Describe sources of financial or non-financial support for the review, and the role of the funders or sponsors in the review. | This work has been supported by Thailand Science Research and Innovation Fund (Contract No. FRB650082/0227) awarded to Assist. Prof. Dr. Thotsapol Thomrongsuwannakij. |
| Competing interests | 26 | Declare any competing interests of review authors. | All authors declare no conflict of interest. |
| Availability of data, code and other materials | 27 | Report which of the following are publicly available and where they can be found: template data collection forms; data extracted from included studies; data used for all analyses; analytic code; any other materials used in the review. | **Supplementary Materials**  **Table S1**: Data extraction of included studies;  **Table S2**: Sensitivity analysis reports the results of Campylobacter prevalence of selected studies;  **Table S3:** Prevalence of *Campylobacter* spp. isolates obtained from different sources of sample collection;  **Table S4:** The sequence types distribution of *C. jejuni* in Thailand;  **Table S5:** The prevalence of phenotypic antimicrobial resistance of *C. jejuni* and *C. coli* in Thailand;  **Table S6**: Sensitivity analysis reports the results of AMR prevalence of *Campylobacter* spp. in included studies.  **Table S7**: The PRISMA 2020 item checklist;  **Table S8**: JBI Critical Appraisal Checklist for minimizing the bias selection of included studies;  **Figure S1**: Forest plots of prevalence of *C. jejuni* and *C. coli* by different sources of sample collection;  **Figure S2**: Forest plots of AMR prevalence of *C. jejuni* and *C. coli* by different sources of sample collection;  **Figure S3:** Contour-enhanced funnel plots and Egger’s tests used for publication bias assessment. |
