# Supplementary material for: Prevalence and antimicrobial resistance of Campylobacter jejuni and Campylobacter coli over time in Thailand under a One Health approach: A systematic review and meta-analysis
Source: One Health. 2025 Jan 10;20:100965. doi: 10.1016/j.onehlt.2025.100965 (PMC11782884; doi:10.1016/j.onehlt.2025.100965)
Supplement: Supplementary Table 4 — Sensitivity analysis reports the results of Campylobacter prevalence of selected studies. [file mmc7.docx]

**Supplementary Table 4**: Sensitivity analysis reports the results of *Campylobacter* prevalence in included studies. Main analysis includes all studies including data collected using assumptions and without assumptions; influential studies: studies with data collected using assumption.

**A. *Campylobacter jejuni***

| **Categories** | **No. prevalence estimates** | ***I^2^* (%)** | **Pooled**  **prevalence**  **(%)** | **95% CI** | ***P-value*** | **Univariable meta-regression analysis** | | |
| --- | --- | --- | --- | --- | --- | --- | --- | --- |
|  |  |  |  |  |  | ***β* (regression coefficient)** | **95% CI** | ***p*-value** |
| **Humans** |  |  |  |  |  |  |  |  |
| ***All human studies*** |  |  |  |  |  |  |  |  |
| Main analysis | 14 | 98.2 | 11.0 | 5.9 - 19.5 | < 0.001 | -0.016 | -0.08-0.05 | 0.593 |
| Influential study removed (n=6) | 8 | 98.9 | 9.7 | 4.1 - 21.4 | < 0.001 | -0.086 | -0.19-0.02 | 0.086 |
| ***Children (diarrhea)*** |  |  |  |  |  |  |  |  |
| Main analysis | 7 | 98.2 | 12.0 | 5.4 - 24.5 | < 0.001 | -0.080 | -0.20-0.04 | 0.136 |
| Influential study removed (n=1) | 6 | 98.3 | 10.7 | 4.2 - 24.8 | < 0.001 | -0.148 | -0.22--0.07 | 0.006* |
| ***General population (diarrhea)*** |  |  |  |  |  |  |  |  |
| Main analysis | 5 | 98.8 | 15.9 | 4.1 - 45.3 | < 0.001 | 0.083 | -0.19-0.36 | 0.405 |
| Influential study removed (n=3) | 2 | 99.7 | 7.1 | 0.0 - 100.0 | < 0.001 | nc | nc | nc |
| ***General population (carriage)*** |  |  |  |  |  |  |  |  |
| Main analysis | 2 | 0.0 | 2.6 | 0.0 - 83.9 | 0.560 | 0.065 | nc | nc |
| Influential study removed (n=2) | 0 | nc | nc | nc | nc | nc | nc | nc |
| **Animals** |  |  |  |  |  |  |  |  |
| ***All animal studies*** |  |  |  |  |  |  |  |  |
| Main analysis | 14 | 98.9 | 22.4 | 8.6-46.9 | <0.001 | 0.034 | -0.18-0.25 | 0.735 |
| Influential study removed (n=4) | 10 | 99.0 | 20.7 | 5.9-52.1 | <0.001 | 0.119 | -0.136-0.373 | 0.313 |
| ***Chicken*** |  |  |  |  |  |  |  |  |
| Main analysis | 8 | 98.6 | 43.6 | 16.3-75.3 | <0.001 | -0.015 | -0.24-0.21 | 0.878 |
| Influential study removed (n=2) | 6 | 99.0 | 38.1 | 8.1-81.1 | <0.001 | 0.036 | -0.32-0.39 | 0.793 |
| ***Duck*** |  |  |  |  |  |  |  |  |
| Main analysis | 4 | 96.8 | 16.7 | 8.4-30.6 | <0.001 | -0.092 | -0.46-0.27 | 0.394 |
| Influential study removed (n=1) | 3 | 80.0 | 13.3 | 7.2-23.3 | 0.007 | -0.029 | -0.80-0.74 | 0.719 |
| ***Ruminant*** |  |  |  |  |  |  |  |  |
| Main analysis | 2 | 40.2 | 1.7 | 0.0-36.9 | 0.196 | 0.270 | nc | nc |
| Influential study removed (n=1) | 1 | nc | nc | nc | nc | nc | nc | nc |
| **Animal products** |  |  |  |  |  |  |  |  |
| ***All animal product studies*** |  |  |  |  |  |  |  |  |
| Main analysis | 15 | 97.4 | 13.3 | 3.8-37.5 | <0.001 | 0.249 | 0.14-0.35 | <0.001* |
| Influential study removed (n=5) | 10 | 97.4 | 27.6 | 12.4-50.8 | <0.001 | 0.141 | 0.05-0.29 | 0.006* |
| ***Chicken products*** |  |  |  |  |  |  |  |  |
| Main analysis | 11 | 98.1 | 31.4 | 14.1-56.0 | <0.001 | 0.167 | 0.06-0.28 | 0.007* |
| Influential study removed (n=3) | 8 | 97.8 | 39.4 | 23.8-57.5 | <0.001 | 0.067 | 0.06-0.19 | 0.045* |
| ***Pork*** |  |  |  |  |  |  |  |  |
| Main analysis | 2 | 0.0 | 0.8 | 0.0-100.0 | 0.999 | 2.22 | nc | nc |
| Influential study removed (n=1) | 1 | nc | nc | nc | nc | nc | nc | nc |
| ***Ruminant products*** |  |  |  |  |  |  |  |  |
| Main analysis | 2 | 0.0 | 0.5 | 0.0-100.0 | 0.999 | 2.16 | nc | nc |
| Influential study removed (n=1) | 1 | nc | nc | nc | nc | nc | nc | nc |
| **Environment** |  |  |  |  |  |  |  |  |
| ***All environment studies*** |  |  |  |  |  |  |  |  |
| Main analysis | 6 | 93.1 | 10.1 | 0.2-36.6 | <0.001 | -0.01 | -0.41-0.39 | 0.953 |
| Influential study removed (n=1) | 5 | 94.4 | 8.8 | 1.2-44.1 | <0.001 | -0.03 | -0.53-0.60 | 0.863 |
| ***Environment samples collected at chicken farm*** |  |  |  |  |  |  |  |  |
| Main analysis | 3 | 97.0 | 6.7 | 0.1-91.9 | <0.001 | 0.07 | -2.80-2.94 | 0.809 |
| Influential study removed (n=0) | 3 | nc | nc | nc | nc | nc | nc | nc |
| ***Environment samples collected at duck farm*** |  |  |  |  |  |  |  |  |
| Main analysis | 3 | 43.2 | 17.1 | 6.4-38.0 | 0.172 | -0.04 | -1.58-1.49 | 0.782 |
| Influential study removed (n=1) | 2 | 71.6 | 15.1 | 0.1-98.3 | 0.061 | nc | nc | nc |

* statistical significance, nc: not calculated

**B. *Campylobacter coli***

|  | **No. prevalence estimates** | ***I^2^* (%)** | **Pooled**  **prevalence**  **(%)** | **95% CI** | ***P-value*** | **Univariable meta-regression analysis** | | |
| --- | --- | --- | --- | --- | --- | --- | --- | --- |
|  |  |  |  |  |  | ***β* (regression coefficient)** | **95% CI** | ***p*-value** |
| **Humans** |  |  |  |  |  |  |  |  |
| ***All human studies*** |  |  |  |  |  |  |  |  |
| Main analysis | 11 | 96.4 | 2.52 | 1.2-5.3 | <0.001 | -0.038 | -0.12-0.04 | 0.288 |
| Influential study removed (n=5) | 6 | 98.1 | 2.54 | 0.9-7.2 | <0.001 | -0.075 | -0.20-0.05 | 0.181 |
| ***Children (diarrhea)*** |  |  |  |  |  |  |  |  |
| Main analysis | 6 | 97.8 | 3.30 | 1.2-9.0 | <0.001 | -0.036 | -0.19-0.12 | 0.558 |
| Influential study removed (n=1) | 5 | 98.2 | 2.94 | 0.8-10.4 | <0.001 | -0.077 | -0.28-0.13 | 0.318 |
| ***General population (diarrhea)*** |  |  |  |  |  |  |  |  |
| Main analysis | 3 | 93.1 | 3.22 | 0.5-18.9 | <0.001 | 0.077 | -0.93-1.09 | 0.509 |
| Influential study removed (n=2) | 1 | nc | nc | nc | nc | nc | nc | nc |
| ***General population (carriage)*** |  |  |  |  |  |  |  |  |
| Main analysis | 2 | 0.0 | 0.00 | 0.0-2.2 | 0.999 | -0.097 | nc | nc |
| Influential study removed (n=1) | 1 | nc | nc | nc | nc | nc | nc | nc |
| **Animals** |  |  |  |  |  |  |  |  |
| ***All animal product studies*** |  |  |  |  |  |  |  |  |
| Main analysis | 11 | 95.6 | 6.24 | 3.1-12.1 | <0.001 | 0.029 | -0.13-0.19 | 0.693 |
| Influential study removed (n=3) | 8 | 91.0 | 5.60 | 2.7-11.2 | <0.001 | 0.043 | -0.11-0.19 | 0.512 |
| ***Chicken*** |  |  |  |  |  |  |  |  |
| Main analysis | 5 | 96.0 | 10.30 | 3.7-25.3 | <0.001 | -0.039 | -0.29-0.21 | 0.660 |
| Influential study removed (n=1) | 4 | 91.3 | 7.86 | 2.8-20.3 | <0.001 | -0.027 | -0.30-0.25 | 0.713 |
| ***Duck*** |  |  |  |  |  |  |  |  |
| Main analysis | 4 | 90.0 | 7.39 | 3.1-16.4 | <0.001 | -0.001 | -0.49-0.49 | 0.992 |
| Influential study removed (n=1) | 3 | 92.1 | 6.21 | 1.5-22.0 | <0.001 | 0.062 | -1.51-1.64 | 0.705 |
| ***Ruminant*** |  |  |  |  |  |  |  |  |
| Main analysis | 2 | 18.3 | 1.4 | 0.0-40.1 | 0.269 | 0.233 | nc | nc |
| Influential study removed (n=1) | 1 | nc | nc | nc | nc | nc | nc | nc |
| **Animal products – Chicken products** |  |  |  |  |  |  |  |  |
| Main analysis | 8 | 97.5 | 10.36 | 3.2-28.9 | <0.001 | 0.031 | -0.24-0.30 | 0.786 |
| Influential study removed (n=2) | 6 | 98.2 | 8,86 | 1.4-39.7 | <0.001 | -0.010 | -0.42-0.40 | 0.949 |
| **Environment** |  |  |  |  |  |  |  |  |
| ***All environment product studies*** |  |  |  |  |  |  |  |  |
| Main analysis | 5 | 96.9 | 5.56 | 0.5-41.7 | <0.001 | -0.369 | -0.85-0.11 | 0.093 |
| Influential study removed (n=1) | 4 | 97.6 | 4.36 | 0.1-67.2 | <0.001 | -0.454 | -1.12-0.21 | 0.100 |
| ***Environment samples collected at chicken farm*** |  |  |  |  |  |  |  |  |
| Main analysis | 2 | 99.2 | 12.57 | 0.0-100.0 | <0.001 | -0.467 | nc | nc |
| Influential study removed (n=0) | 2 | nc | nc | nc | nc | nc | nc | nc |
| ***Environment samples collected at duck farm*** |  |  |  |  |  |  |  |  |
| Main analysis | 3 | 0.0 | 4.29 | 0.2-46.7 | 0.682 | -0.390 | -3.04-2.26 | 0.313 |
| Influential study removed (n=1) | 2 | 0.0 | 2.10 | 0.0-99.9 | 0.999 | nc | nc | nc |

* statistical significance, nc: not calculated
