# Supplementary material for: Prevalence and antimicrobial resistance of Campylobacter jejuni and Campylobacter coli over time in Thailand under a One Health approach: A systematic review and meta-analysis
Source: One Health. 2025 Jan 10;20:100965. doi: 10.1016/j.onehlt.2025.100965 (PMC11782884; doi:10.1016/j.onehlt.2025.100965)
Supplement: Supplementary Table 8 — Sensitivity analysis reports the results of AMR prevalence of Campylobacter spp. in included studies. [file mmc11.docx]

**Supplementary Table 8**. Sensitivity analysis reports the results of AMR prevalence of *Campylobacter* spp. in included studies. Main analysis includes all studies including data collected using assumptions and without assumptions; influential studies: studies with data collected using assumption

**A. Ampicillin**

| **Categories** | **No. prevalence estimates** | ***I^2^* (%)** | **Pooled**  **prevalence**  **(%)** | **95% CI** | ***P-value*** | **Univariable meta-regression analysis** | | |
| --- | --- | --- | --- | --- | --- | --- | --- | --- |
|  |  |  |  |  |  | ***β* (regression coefficient)** | **95% CI** | ***p*-value** |
| ***C. jejuni*** |  |  |  |  |  |  |  |  |
| ***General population (diarrhea)*** |  |  |  |  |  |  |  |  |
| Main analysis | 2 | 0.0 | 31.4 | 5.8-77.4 | 0.645 | 0.165 | nc | nc |
| Influential study removed (n=1) | 1 | nc | nc | nc | nc | nc | nc | nc |
| ***Chicken*** |  |  |  |  |  |  |  |  |
| Main analysis | 4 | 3.4 | 26.5 | 15.8-41.0 | 0.376 | 0.058 | -0.09-0.21 | 0.242 |
| Influential study removed (n=3) | 1 | nc | nc | nc | nc | nc | nc | nc |
| ***Chicken products*** |  |  |  |  |  |  |  |  |
| Main analysis | 2 | 0.0 | 45.0 | 4.5-93.4 | 0.383 | -0.096 | nc | nc |
| Influential study removed (n=1) | 1 | nc | nc | nc | nc | nc | nc | nc |

nc: not calculated

**B. Azithromycin**

| **Categories** | **No. prevalence estimates** | ***I^2^* (%)** | **Pooled**  **prevalence**  **(%)** | **95% CI** | ***P-value*** | **Univariable meta-regression analysis** | | |
| --- | --- | --- | --- | --- | --- | --- | --- | --- |
|  |  |  |  |  |  | ***β* (regression coefficient)** | **95% CI** | ***p*-value** |
| ***C. jejuni*** |  |  |  |  |  |  |  |  |
| ***Children (diarrhea)*** |  |  |  |  |  |  |  |  |
| Main analysis | 3 | 0.0 | 1.7 | 0.6-4.7 | 0.611 | -0.061 | -2.68-2.56 | 0.817 |
| Influential study removed (n=3) | 0 | nc | nc | nc | nc | nc | nc | nc |
| ***General population (diarrhea)*** |  |  |  |  |  |  |  |  |
| Main analysis | 3 | 0.0 | 2.0 | 0.2-15.5 | 0.456 | 0.134 | -1.09-1.35 | 0.396 |
| Influential study removed (n=2) | 1 | nc | nc | nc | nc | nc | nc | nc |
| ***C. coli*** |  |  |  |  |  |  |  |  |
| ***Children (diarrhea)*** |  |  |  |  |  |  |  |  |
| Main analysis | 3 | 65.4 | 18.5 | 7.3-39.6 | 0.056 | 0.233 | -1.57-2.04 | 0.348 |
| Influential study removed (n=3) | 0 | nc | nc | nc | nc | nc | nc | nc |

nc: not calculated

**C. Ciprofloxacin**

| **Categories** | **No. prevalence estimates** | ***I^2^* (%)** | **Pooled**  **prevalence**  **(%)** | **95% CI** | ***P-value*** | **Univariable meta-regression analysis** | | |
| --- | --- | --- | --- | --- | --- | --- | --- | --- |
|  |  |  |  |  |  | ***β* (regression coefficient)** | **95% CI** | ***p*-value** |
| ***C. jejuni*** |  |  |  |  |  |  |  |  |
| ***Children (diarrhea)*** |  |  |  |  |  |  |  |  |
| Main analysis | 4 | 84.0 | 77.9 | 62.2-88.3 | <0.001 | 0.138 | -0.21-0.49 | 0.230 |
| Influential study removed (n=3) | 1 | nc | nc | nc | nc | nc | nc | nc |
| ***General population (diarrhea)*** |  |  |  |  |  |  |  |  |
| Main analysis | 3 | 0.0 | 91.3 | 77.9-96.9 | 0.924 | 0.191 | -2.80-3.18 | 0.566 |
| Influential study removed (n=2) | 1 | nc | nc | nc | nc | nc | nc | nc |
| ***Chicken*** |  |  |  |  |  |  |  |  |
| Main analysis | 6 | 89.1 | 88.7 | 71.3-96.1 | <0.001 | 0.119 | 0.07-0.17 | 0.004* |
| Influential study removed (n=3) | 3 | 94.6 | 89.6 | 35.0-99.3 | <0.001 | 0.112 | -0.14-0.36 | 0.111 |
| ***Chicken products*** |  |  |  |  |  |  |  |  |
| Main analysis | 3 | 78.0 | 71.6 | 40.2-90.4 | 0.011 | 0.078 | -0.25-0.41 | 0.206 |
| Influential study removed (n=1) | 2 | 88.9 | 70.7 | 23.5-99.6 | 0.003 | 0.078 | nc | nc |
| ***C. coli*** |  |  |  |  |  |  |  |  |
| ***Children (diarrhea)*** |  |  |  |  |  |  |  |  |
| Main analysis | 3 | 65.4 | 83.8 | 32.5-98.2 | 0.055 | 0.472 | -3.96-4.90 | 0.405 |
| Influential study removed (n=3) | 0 | nc | nc | nc | nc | nc | nc | nc |

*statistical significance, nc: not calculated

**D. Erythromycin**

| **Categories** | **No. prevalence estimates** | ***I^2^* (%)** | **Pooled**  **prevalence**  **(%)** | **95% CI** | ***P-value*** | **Univariable meta-regression analysis** | | |
| --- | --- | --- | --- | --- | --- | --- | --- | --- |
|  |  |  |  |  |  | ***β* (regression coefficient)** | **95% CI** | ***p*-value** |
| ***C. jejuni*** |  |  |  |  |  |  |  |  |
| ***Children (diarrhea)*** |  |  |  |  |  |  |  |  |
| Main analysis | 4 | 96.7 | 6.8 | 0.5-53.9 | <0.001 | -0.213 | -0.55-0.12 | 0.110 |
| Influential study removed (n=3) | 2 | 94.2 | 22.7 | 0.0-100.0 | <0.001 | -0.154 | nc | nc |
| ***General population (diarrhea)*** |  |  |  |  |  |  |  |  |
| Main analysis | 2 | 36.3 | 2.7 | 0.0-94.5 | 0.210 | 0.116 | nc | nc |
| Influential study removed (n=1) | 1 | 3nc | nc | nc | nc | nc | nc | nc |
| ***Chicken*** |  |  |  |  |  |  |  |  |
| Main analysis | 5 | 84.8 | 5.3 | 0.2-67.7 | <0.001 | -0.147 | -0.56-0.26 | 0.336 |
| Influential study removed (n=2) | 3 | 92.4 | 10.46 | 0.0-97.8 | <0.001 | -0.142 | -1.34-1.06 | 0.374 |
| ***Chicken products*** |  |  |  |  |  |  |  |  |
| Main analysis | 3 | 89.8 | 0.9 | 0.4-73.7 | <0.0001 | -0.200 | 1.36-0.96 | 0.273 |
| Influential study removed (n=3) | 0 | nc | nc | nc | nc | nc | nc | nc |
| ***C. coli*** |  |  |  |  |  |  |  |  |
| ***Children (diarrhea)*** |  |  |  |  |  |  |  |  |
| Main analysis | 3 | 92.9 | 40.9 | 0.6-98.8 | <0.001 | -0.302 | -1.25-0.65 | 0.154 |
| Influential study removed (n=2) | 1 | nc | nc | nc | nc | nc | nc | nc |
| ***Chicken*** |  |  |  |  |  |  |  |  |
| Main analysis | 2 | 0.0 | 2.5 | 0.0-100.0 | 0.999 | 4.679 | nc | nc |
| Influential study removed (n=0) | 2 | nc | nc | nc | nc | nc | nc | nc |

nc: not calculated

**E. Gentamicin**

| **Categories** | **No. prevalence estimates** | ***I^2^* (%)** | **Pooled**  **prevalence**  **(%)** | **95% CI** | ***P-value*** | **Univariable meta-regression analysis** | | |
| --- | --- | --- | --- | --- | --- | --- | --- | --- |
|  |  |  |  |  |  | ***β* (regression coefficient)** | **95% CI** | ***p*-value** |
| ***C. jejuni*** |  |  |  |  |  |  |  |  |
| ***Chicken*** |  |  |  |  |  |  |  |  |
| Main analysis | 2 | 0.0 | 0.0 | 0.0-100.0 | 0.999 | 0.249 | nc | nc |
| Influential study removed (n=1) | 1 | nc | nc | nc | nc | nc | nc | nc |
| ***Chicken products*** |  |  |  |  |  |  |  |  |
| Main analysis | 2 | 89.1 | 5.8 | 0.0-99.9 | 0.003 | -0.183 | nc | nc |
| Influential study removed (n=0) | 2 | nc | nc | nc | nc | nc | nc | nc |

nc: not calculated

**F. Nalidixic acid**

| **Categories** | **No. prevalence estimates** | ***I^2^* (%)** | **Pooled**  **prevalence**  **(%)** | **95% CI** | ***P-value*** | **Univariable meta-regression analysis** | | |
| --- | --- | --- | --- | --- | --- | --- | --- | --- |
|  |  |  |  |  |  | ***β* (regression coefficient)** | **95% CI** | ***p*-value** |
| ***C. jejuni*** |  |  |  |  |  |  |  |  |
| ***Children (diarrhea)*** |  |  |  |  |  |  |  |  |
| Main analysis | 4 | 98.2 | 75.4 | 46.9-91.4 | <0.001 | 0.174 | -0.40-0.75 | 0.323 |
| Influential study removed (n=3) | 1 | nc | nc | nc | nc | nc | nc | nc |
| ***General population (diarrhea)*** |  |  |  |  |  |  |  |  |
| Main analysis | 3 | 0.0 | 94.9 | 82.2-98.7 | 0.943 | 0.183 | -3.58-3.95 | 0.649 |
| Influential study removed (n=2) | 1 | nc | nc | nc | nc | nc | nc | nc |
| ***Chicken*** |  |  |  |  |  |  |  |  |
| Main analysis | 4 | 92.3 | 87.3 | 44.7-98.3 | <0.001 | 0.161 | 0.05-0.28 | 0.027* |
| Influential study removed (n=1) | 3 | 94.8 | 89.9 | 17.6-99.7 | <0.001 | 0.162 | -0.18-0.50 | 0.105 |
| ***Chicken products*** |  |  |  |  |  |  |  |  |
| Main analysis | 2 | 62.0 | 75.6 | 19.3-97.6 | 0.105 | 0.044 | nc | nc |
| Influential study removed (n=0) | 2 | nc | nc | nc | nc | nc | nc | nc |
| ***C. coli*** |  |  |  |  |  |  |  |  |
| ***Children (diarrhea)*** |  |  |  |  |  |  |  |  |
| Main analysis | 3 | 92.5 | 80.9 | 11.7-99.3 | <0.001 | 0.707 | -.084-2.26 | 0.109 |
| Influential study removed (n=3) | 0 | nc | nc | nc | nc | nc | nc | nc |

*statistical significance, nc: not calculated

**G. Trimethoprim-Sulfamethoxazole**

| **Categories** | **No. prevalence estimates** | ***I^2^* (%)** | **Pooled**  **prevalence**  **(%)** | **95% CI** | ***P-value*** | **Univariable meta-regression analysis** | | |
| --- | --- | --- | --- | --- | --- | --- | --- | --- |
|  |  |  |  |  |  | ***β* (regression coefficient)** | **95% CI** | ***p*-value** |
| ***C. jejuni*** |  |  |  |  |  |  |  |  |
| ***General population (diarrhea)*** |  |  |  |  |  |  |  |  |
| Main analysis | 2 | 94.6 | 37.4 | 0.0-99.9 | <0.001 | -1.762 | nc | nc |
| Influential study removed (n=1) | 1 | nc | nc | nc | nc | nc | nc | nc |
| ***Chicken*** |  |  |  |  |  |  |  |  |
| Main analysis | 2 | 78,5 | 51.3 | 0.6-99.5 | 0.031 | -0.089 |  |  |
| Influential study removed (n=0) | 2 | nc | nc | nc | nc | nc | nc | nc |

**H. Tetracycline**

| **Categories** | **No. prevalence estimates** | ***I^2^* (%)** | **Pooled**  **prevalence**  **(%)** | **95% CI** | ***P-value*** | **Univariable meta-regression analysis** | | |
| --- | --- | --- | --- | --- | --- | --- | --- | --- |
|  |  |  |  |  |  | ***β* (regression coefficient)** | **95% CI** | ***p*-value** |
| ***C. jejuni*** |  |  |  |  |  |  |  |  |
| ***Children (diarrhea)*** |  |  |  |  |  |  |  |  |
| Main analysis | 2 | 93.7 | 35.4 | 0.0-99.9 | <0.001 | 0.114 | nc | nc |
| Influential study removed (n=0) | 2 | nc | nc | nc | nc | nc | nc | nc |
| ***General population (diarrhea)*** |  |  |  |  |  |  |  |  |
| Main analysis | 2 | 74.0 | 77.2 | 10.0-99.0 | 0.050 | -0.751 | nc | nc |
| Influential study removed (n=1) | 1 | nc | nc | nc | nc | nc | nc | nc |
| ***Chicken*** |  |  |  |  |  |  |  |  |
| Main analysis | 6 | 90.4 | 52.8 | 25.5-78.5 | <0.001 | 0.081 | -0.05-0.21 | 0.155 |
| Influential study removed (n=3) | 3 | 63.8 | 63.3 | 36.9-83.6 | 0.063 | 0.044 | -0.21-0.30 | 0.275 |
| ***Chicken products*** |  |  |  |  |  |  |  |  |
| Main analysis | 3 | 92.6 | 49.5 | 7.4-92.3 | <0.001 | -0.125 | -0.45-0.21 | 0.131 |
| Influential study removed (n=1) | 2 | 95.9 | 59.4 | 0.0-99.9 | <0.001 | -0.139 | nc | nc |
| ***C. coli*** |  |  |  |  |  |  |  |  |
| ***Chicken*** |  |  |  |  |  |  |  |  |
| Main analysis | 2 | 0.0 | 97.0 | 1.9-100.0 | 0.495 | -0.143 | nc | nc |
| Influential study removed (n=0) | 2 | nc | nc | nc | nc | nc | nc | nc |
